# Designing High-Refractive Index Polymers Using Materials Informatics †

**DOI:** 10.3390/polym10010103

**Published:** 2018-01-22

**Authors:** Vishwesh Venkatraman, Bjørn Kåre Alsberg

**Affiliations:** Department of Chemistry, Norwegian University of Science and Technology (NTNU), 7491 Trondheim, Norway

**Keywords:** computer-aided design, high refractive index polymer, QSPR, machine learning

## Abstract

A machine learning strategy is presented for the rapid discovery of new polymeric materials satisfying multiple desirable properties. Of particular interest is the design of high refractive index polymers. Our in silico approach employs a series of quantitative structure–property relationship models that facilitate rapid virtual screening of polymers based on relevant properties such as the refractive index, glass transition and thermal decomposition temperatures, and solubility in standard solvents. Exploration of the chemical space is carried out using an evolutionary algorithm that assembles synthetically tractable monomers from a database of existing fragments. Selected monomer structures that were further evaluated using density functional theory calculations agree well with model predictions.

## 1. Introduction

Polymeric thin films with high refractive indices and high optical clarity are highly sought after in various applications such as optical data storage [1], lenses [2], anti-reflective coatings [3], immersion lithography [4] and complementary metal-oxide-semiconductor (CMOS) image sensors [5].

The trends and developments in the field have been summarized in recent reviews [6,7] and the references therein. In addition to a high refractive index, other properties of interest are the thermal stability related parameters, such as the glass transition temperatures ($T_{g}>100$ ${}^{\circ }$C [8]) and thermal decomposition temperatures ($T_{d}>200$ ${}^{\circ }$C) that play a critical role in the optical device fabrication. Given the performance requirements that need to be satisfied for different applications, the polymer chemist is faced with the challenge of finding a balance between several complementary properties.

A popular strategy for developing high refractive index polymers (HRIP) is based on using inorganic nanoparticle-filled polymer composites [9]. Although promising, they pose significant processing challenges and suffer from high optical losses. A more effective approach has been to alter the chemical structure of the polymer by incorporating high-molar refraction groups, such as sulfur atoms and aromatic structures [7]. With a view to rapidly explore the vast chemical space and thereby fine tune experimental efforts, there has been increased focus on using computational approaches. Semi-empirical electronic structure methods, for instance, have been used to create a database of porous polymer networks that can be used as methane adsorbents [10]. In other studies, ab initio approaches were used to search for polymer dielectrics [11] and also in the design of polymers for photovoltaic applications [12]. However, the computational costs associated with ab initio methods limit large scale explorations of the chemical space. Inverse Quantitative Structure-Property Relationship (QSPR) approaches such as those based on the signature molecular descriptor [13] have also been employed but require significant computational effort to solve constraint equations.

More computationally expedient alternatives to standard quantum chemistry (QC) based schemes for property prediction include those based on data-driven approaches that make use of machine learning (ML), multivariate statistics and chemometrics. These methods typically rely on establishing quantitative structure–property relationships [14] (QSPR) and can be used for modelling diverse properties including those that at present cannot be directly computed using QC (such as the solar power conversion efficiency [15]). Starting with a representative set of molecules, each structure is encoded as a vector using a wide range of descriptors that capture geometrical and electronic properties. Each vector can be associated with one or more responses or class labels that are to be predicted. The vectors and their responses are stored in matrices that are submitted to ML algorithms [14] that perform regression or classification to establish a mathematical model between the structure descriptor variables and the responses. The purpose of the QSPR is two-fold: to discover interesting relationships between the structure descriptors and the responses and, secondly, as a way to obtain rapid estimations of the responses. The latter is of particular interest in combinatorial [16] and evolutionary [15,17] searches in chemical space as the number of possible structures to investigate would -be intractable for most QC approaches. Thus, using QSPR models to predict relevant responses facilitates searching a much larger chemical space using fewer computational resources than traditional QC approaches.

In this article, we adopt an in silico molecular design approach that is based on principles of Darwinian evolution to drive the search for HRIPs. In the past, approaches based on evolutionary algorithms have been successfully applied to the design of drug-like molecules [18], dyes for solar cells [15] and olefin metathesis catalysts [19]. In recent years, a number of polymer properties relevant for optoelectronic applications such as the refractive index [20,21,22], glass transition temperature [23] and thermal decomposition temperature [24] have been modelled using QSPR methods wherein descriptors calculated from the monomer structures have been correlated with the property of interest. Continuing with this approach, we make use of such models to carry out a multiple criteria-based virtual screening of polymers. As opposed to the largely intuition-driven trial and error approaches, we adopt a systems approach advocated by Bicerano [25]. The computational search strategy used in this study facilitates the accelerated discovery of advanced optical materials in a cost-effective manner. The most promising repeat units are further subjected to rigorous validation based on density functional theory [26] (DFT). Application of this multi-pronged strategy has yielded a number of promising monomers with advantageous properties and may be of considerable value in the development of photonic materials.

## 2. Materials and Methods

**Figure 1:**
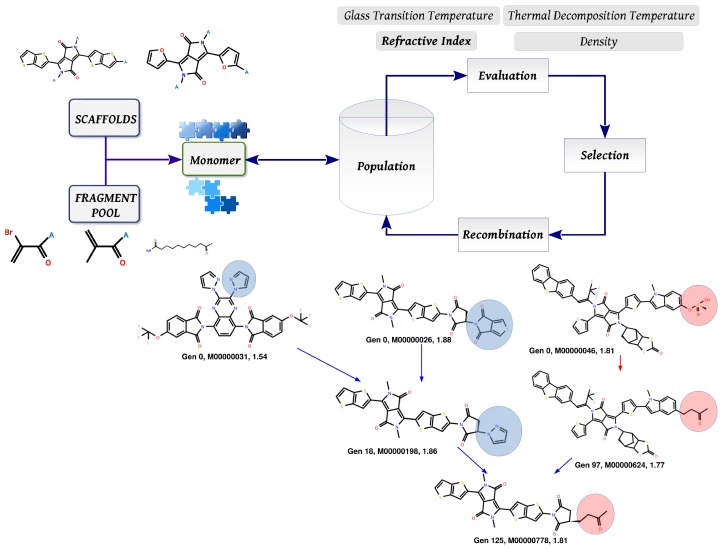
Schematic shows an outline of the design approach. The “A” on the fragments indicate points of attachment. The crossover operations (indicated by blue arrows) involve random selection of fragments (building blocks highlighted by the circles) in the two parent structures and swaps them, thus producing typically two offspring. In a given structure, the mutation operator (red arrow) may either replace or delete a randomly selected fragment.

**Figure 2:**
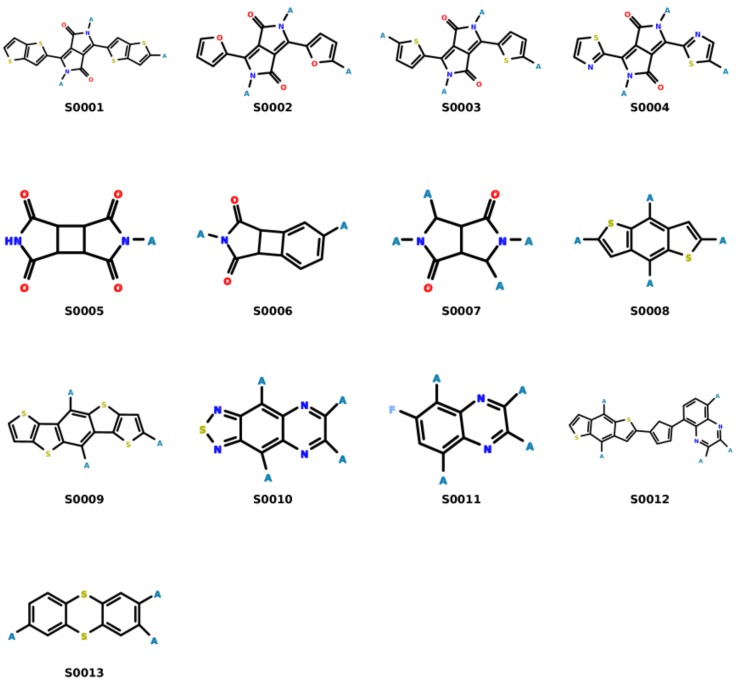
The building block scaffolds used in this study. The attachment points are indicated by the letter “A”. The fragment recombination proceeds according to a set of fragment compatibility rules [29].

An overview of the molecular design approach based on Darwinian evolution is depicted in Figure 1. The process has been discussed in detail in previous articles [15,19,27,28] and only a short description is provided here. Each proposed structure (repeating unit) is defined as a combination of fragments attached to a scaffold that has an available set of attachment points (shown as “A” in the figure). The building blocks are connected with respect to a set of pre-defined rules so as to maximize the probability of synthesis [29,30]. The molecular fragments were generated by applying the Breaking of Retrosynthetically Interesting Chemical Substructures (BRICS) [31] fragmentation algorithm to existing monomer structures taken from various literature sources. Figure 2 lists the various scaffolds used as starting points to create different monomers. Each structure output by the evolutionary protocol is then evaluated in terms of the value of the refractive index (the primary fitness estimate), which is obtained here using an ML model. Over several iterations of the evolutionary algorithm, the population of monomer structures undergoes computational crossover and mutation operations involving fragment exchange or substitution. In addition to the refractive index, other properties such as the $T_{g}$, $T_{d}$ and solubility in standard solvents (*N*-methyl-2-pyrrolidone (NMP), chloroform), etc. are also evaluated using different machine learning models. Finally, selected candidates that pass initial criteria are further analyzed using density functional theory approaches [26].

### 2.1. Polymer Properties

The refractive index (*n*) of a polymer is often expressed in terms of the Lorentz–Lorenz [32,33] equation given by:

(1) $$\frac{n^{2}-1}{n^{2}+2}=\frac{4\pi }{3}\frac{\rho N_{A}}{M_{w}}\alpha ,$$

where α is the linear molecular polarizability, ρ the polymer density, $M_{w}$ the molecular weight of the monomer and $N_{A}$ the Avogadro number [34,35]. The equation thus enables the prediction of the refractive index, using the polarizability of an isolated molecule.

An important parameter in optics and design of lenses is the Abbe number ($v_{d}$) [8], which is a measure of the refractive index dispersion (larger numbers correspond to a lower dispersion) and is given by:

(2) $$v_{d}=\frac{n_{D}-1}{n_{F}-n_{C}},$$

where $n_{D}$, $n_{F}$, and $n_{C}$ are the refractive indices of the material at the wavelengths of the Fraunhofer *D* (589.3 nm), *F* (486.1 nm) and *C* (656.3 nm) spectral lines, respectively.

In addition to the *n* and $v_{d}$, other properties to be considered include birefringence and optical transparency [7]. The birefringence, calculated as $\Delta n=n_{TE}-n_{TM}$ (where $n_{TE}$ and $n_{TM}$ are the in-plane and out-of-plane refractive indices, respectively), is caused by the orientation of polymer molecular chains and is required to be minimal, in order to achieve fine focusing in lenses. Furthermore, for use in optoelectronic materials, a high transparency in the visible range is desirable [36]. Thus, the spectra of new monomer structures should have minimal absorbance in the visible region (400–700 nm).

### 2.2. Machine Learning

Experimental values of the refractive index (*n* at 589 nm), density (ρ at room temperature), glass transition temperatures ($T_{g}$) and thermal decomposition temperatures ($T_{d}$ 10% weight loss temperatures recorded in N${}_{2}$ atmosphere) for a diverse set of polymers were collated from existing literature [20,23,25,37,38]. A complete list of the monomer structures and corresponding experimental values are provided in the Supplementary Materials (see Tables S2–S6). Table 1 summarizes the available data for the properties studied. The chemistry spans several classes including polyimides, polyethylenes, polyphosphazenes, polyacrylates, polyarylene sulfides, phenylquinoxalines, polystyrenes and polycarbonates.

Given the previous success of quantum chemical descriptors in modelling polymer properties [39,40,41] based on the structure of the monomer, we employ these to model the different properties. For each monomer, various geometrical and molecular orbital-based descriptors such as the Highest Occupied Molecular Orbital/Lowest Unoccupied Molecular Orbital (HOMO/LUMO) energies, charges, polarizabilities, superdelocalizabilities, and radial distribution function (RDF) indices were calculated using the software KRAKENX (version 0.1.3, www.krakenminer.com) [42]. These descriptors have earlier been shown to be well suited for predicting diverse properties such as power conversion efficiencies of dyes in solar cells [17,43], densities/viscosities [42], p$K_{a}$ [27] and thermal decomposition temperatures of ionic liquids [44]. A total of 828 descriptors was calculated for each monomer, which was reduced to around 818, after the removal of low variance columns and those containing missing values. Table S1 in the Supplementary Materials provides a description of the variables.

The data fitting was carried out using both linear partial least squares regression [45] (PLSR) and the ensemble tree-based random forests (RF) [46] method. In order to assess the predictive abilities of the ML models, the data was split (50:50) randomly for each model. As part of the preprocessing, a pairwise correlation analysis was performed and only one among the highly correlated pair of variables ($R^{2}>0.95$) was retained. The remaining variables (<400) were then autoscaled to zero mean and unit variance. Multiple metrics: the root mean squared error (RMSE), mean absolute error (MAE), and 10-fold cross-validated correlation coefficient $R_{cv}^{2}$ were used to evaluate the model performances. In addition, variable selection was also carried out to improve the predictive ability and, where possible, reduce model complexity (see previous papers [15,42,44]). The generated models are constrained by the response and chemical structure space within which they are assumed to reliable. To establish reliability estimates for the PLSR model predictions, the distance to the model [47] and the bootstrap variance [15] based on 500 models was computed, while, for the random forests, the conditional quantiles [48] were used. Predictions for which the estimated variability is small can in general be trusted while those with large values need to be treated with caution.

### 2.3. Computational Details

The structures of the monomers were drawn using the MarvinSketch [49] program version 5.9.3 from ChemAxon, https://chemaxon.com/ (or alternatively taken from literature when available) were converted to 3D using OpenBabel (version 2.4.1, http://openbabel.org/docs/current/) [50] (based on the Universal Force Field [51]). The initial geometries were further optimized using the semi-empirical AM1 Hamiltonian in MOPAC (version 16.220L, http://openmopac.net/) [52]. For the refractive index calculations, the MOPAC optimized structures were further subjected to full geometry optimizations at the DFT level (without symmetry constraints) using the B3LYP [53] functional and the 6-311G(d,p) basis set. The wavelength-dependent linear polarizabilities were computed using the range-corrected CAM-B3LYP [54] functional along with the 6-311++G(d,p) [55] basis set (containing both polarisation and diffuse functions). To assess optical transparency, the UV-Vis absorption spectra [56] of the monomers was computed using time-dependent DFT (TDDFT) carried out at the CAM-B3LYP/6-311G(d,p) level of theory. The DFT calculations were performed using the Gaussian 09 [57] software package. The regression models were developed using the statistical software *R* version 3.4.2, https://www.r-project.org/ [58] with the packages *pls* [59] and *randomForest* [48,60].

## 3. Results and Discussion

### 3.1. Analysis of Regression Models

Table 2 summarizes the results for the regression models corresponding to the *n*, $T_{g}$, ρ and $T_{d}$. The complete list of experimental and predicted values is provided in Tables S2–S6 in the Supplementary Materials. The PLSR model applied to the refractive index prediction yields a low-complexity 4 latent variable (LV) model, which performs quite well for both the calibration and independent test sets with $R^{2}∼0.80$. While similar metrics for the $T_{g}$ are achieved, the PLSR model performance for $T_{d}$ ($R^{2}∼0.60$) is considerably poorer. In comparison, the random forests’ (based on 100 trees) regression models perform well on most properties. In general, tree-based models are less useful for extrapolation as they employ boxing in selected regions of the variable space. Given the interest in identifying HRIPs (with *n* > 1.70) for which the models are required to extrapolate beyond the calibration range of the response variable, we make use of PLSR models for driving the search for suitable polymers. Since both the experimental $T_{g}$ and $T_{d}$ values span a desirable temperature range, the RF models act as filters by excluding those that have predicted values below a given threshold. Predictive models for the density were found to be comparatively poorer with the RF model yielding calibration statistics of $R_{cv}^{2}=0.64$ and a test set $R^{2}=0.66$, while PLSR failed to produce models with $R_{cv}^{2}>0.50$. Attempts to improve the performance using other methods such as support vector machines [61], however, did not meet with success.

**Figure 3:**
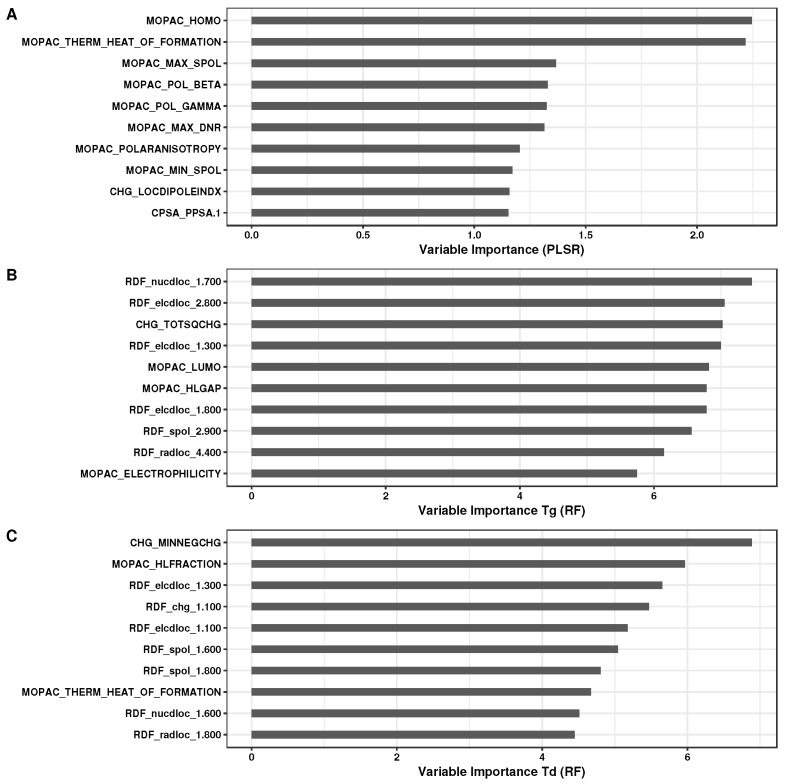
Variable importance plots for the (**A**) Partial Least Squares Regression (PLSR) model for *n*, and Random Forest (RF) models for (**B**) $T_{g}$ and (**C**) $T_{d}$. In all cases, only the top 10 most important variables are shown.

Figure 3 shows the variable importance plots for the regression models and is based on the contribution the predictor variables make to the construction of the models. For PLSR, the ranking is based on the variable importance in projection score [62] (VIP), while, for RF models, the importance is calculated based on the increase in the mean square error of predictions as a result of a given descriptor being randomly permuted [63]. The PLSR model for the refractive index *n* show the most important variables to be the heat of formation (at the AM1 level of theory) that reflects the thermodynamic stability of the polymer, the global softness (the inverse of the HOMO-LUMO energy gap), the nucleophilic (DNR) and electrophilic (DER) delocalizabilities that are dynamic reactivity indices and signify intermolecular interaction, and the static hyperpolarizability that influences the electric susceptibility [64]. A small HOMO-LUMO energy gap may also indicate that the molecule is easily polarized. In addition, a number of charge surface area descriptors that emphasize the charge distribution can be linked to the size related bulk properties of the repeating unit [39]. For both $T_{g}$ and $T_{d}$ models, the prominent features are dominated by variables that emphasize electrophilic and nucleophilic attack along with other values such as the heat of formation, the global electrophilicity index [65] (measures the energy stabilization) and the HOMO-LUMO gap that are seen as standard indicators of stability. The charge based descriptors, on the other hand, may reflect the electrostatic interactions between the polymer chains. Many of the highlighted variables mirror the findings in previous studies [39,40,41].

### 3.2. Molecular Evolution Analysis

**Figure 4:**
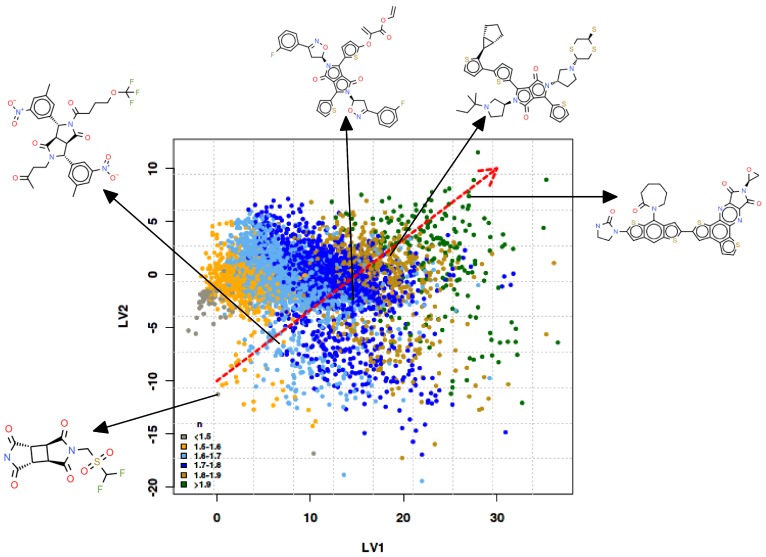
Scores plot for the first two latent variables shows the spread of the predicted refractive indices for the designed monomers. The dashed arrow in the centre of the plot shows the direction of the increasing refractive indices as indicated by the PLSR model. Structures of selected monomers along this line reflect the chemical diversity in the population.

The evolutionary algorithm was configured to run with a population of 100 structures for a maximum of 100 generations with crossover and mutation probabilities set to 0.5. In order to prevent the monomers from becoming too large, a molecular weight restriction was imposed wherein structures above 1000 daltons were discarded. Over 4000 unique structures were produced from five runs of the evolutionary algorithm initialized with different starting seeds. For these monomers, the predicted values of *n* (at λ = 589.3 nm) ranged between 1.40 to 2.30 (see Figure S1 in the Supplementary Materials), with approximately 40% of the structures yielding n>1.68, the maximum *n* value in the calibration data set. In order to examine the trends in the predicted response, an analysis of the PLSR latent variable scores was performed. Figure 4 shows the increasing trend of the refractive indices along the first two latent vectors. While the presence of conjugated ring structures and sulphur content have been shown to increase *n*, other factors such as the higher molecular weight of monomers have also been seen to influence *n* (see Figures S2 and S3 in the Supplementary Materials). Similar trends are seen for the designed structures with nearly one-fourth of the monomers having *n* > 1.70 and 400≤MW≤1000. Although the molecular weight descriptor was removed during the model building (highly correlated with other variables and hence excluded), it is interesting to note that the model nonetheless captures the variations in the refractive index with respect to the chemical composition reasonably well.

**Figure 5:**
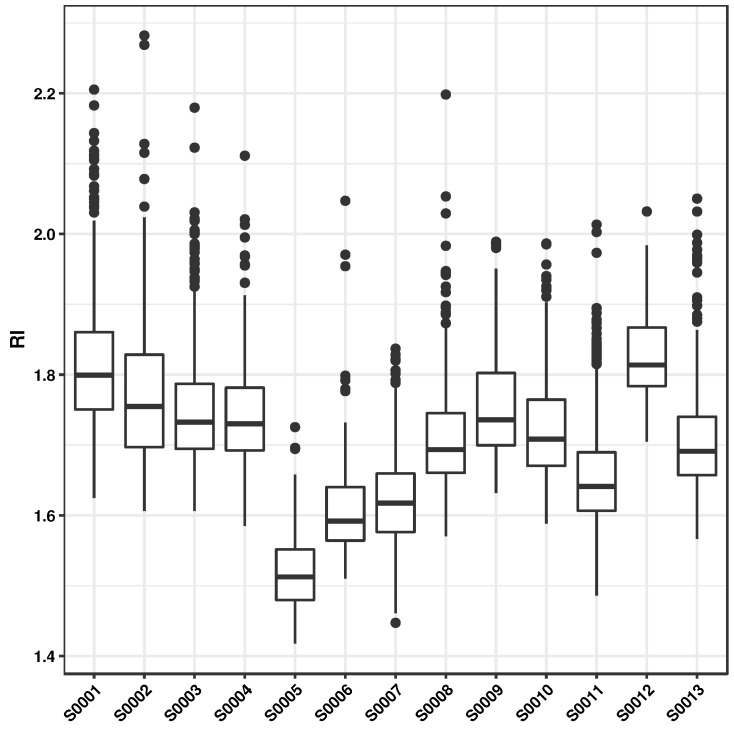
The box plot shows the range of refractive index values with respect to each scaffold (see Figure 2).

Analysis of the monomers based on the different scaffolds (shown in Figure 2) used show that structures based on nitrogen or sulfur-containing substituents generally yielded high refractive indices (*n* > 1.7) [7,66]. The thiazole moiety (comprising of a sulfur atom and a C=N–C bond) not only increases the sulphur content but also leads to low molar volumes, thereby yielding high refractive indices [67,68]. Furthermore, scaffolds based on the diketopyrrolopyrrole (see Figure 5) (linked with thiazole, furan, thiophene and thienothiophene), π-conjugated benzodithiophene and thianthrene moieties are seen to have high refractive indices. Incorporating these units has been seen to improve thermal stability and solubility.

Although the molecular assembly attempts to create structures that are likely to be synthetically tractable, it is interesting to assess it quantitatively with a synthetic accessibility (SA) score. Here, we use the ease of synthesis ranging between 1 (easy to make) to 10 (difficult) as a metric to guage the synthetic accessibility of the proposed monomers [69]. A Python-based implementation [70] that combines fragment contributions and complexity penalties was used to estimate the SA score. For a significant majority of the structures, the score was found to be around 5 or less (see Figure S4 in the Supplementary Materials).

**Figure 6:**
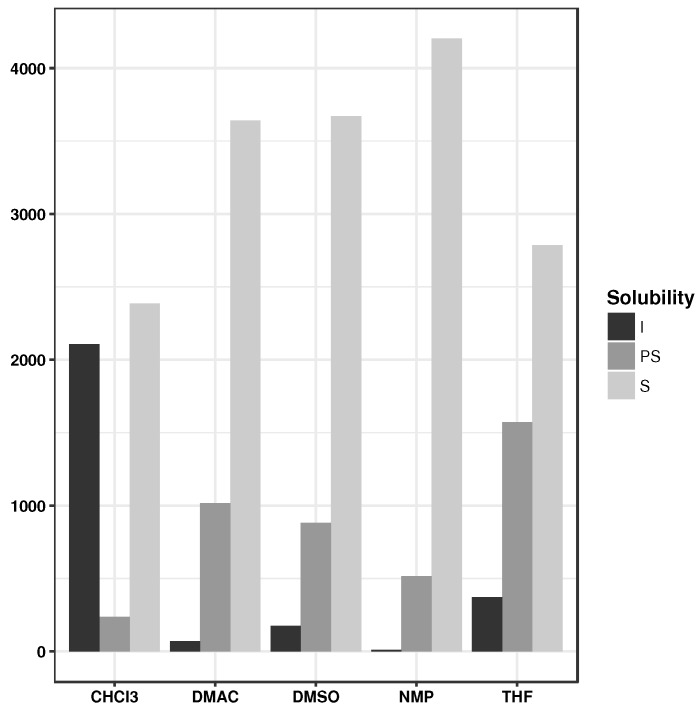
Predicted solubilities for the de novo designed monomers.

Another important criterion to consider is that of the solubility of the polymer in common organic solvents [71]. Poor solubility makes processing difficult and limits their applications. Solubility data for polymers with respect to five commonly used solvents—chloroform (CHCl${}_{3}$), dimethylacetamide (DMAc), *N*-methylpyrrolidine (NMP), tetrahydrofuran (THF) and dimethyl sulfoxide (DMSO)—were collated from the literature. The available data was divided into three classes, which include: S, soluble, PS, partially soluble/swelling/soluble on heating and I, insoluble. For each solvent, the data were equally divided into independent calibration and test sets across the different classes. Random forests classification models were created to predict the solubility class (I/PS/S) using the descriptors as described above. Since the classes are unevenly distributed for all solvents, we make use of Cohen’s Kappa statistic [72], which can be applied to both multi-class and imbalanced class problems. Model performances summarized in Table 3 show that the typical κ values are in the range 0.50–0.60 and fall in the moderate agreement range [72]. Using these models, the solubility classes for the designed monomers were predicted. As can be seen from Figure 6, a majority (>70%) of the proposed structures are potentially soluble in DMAc, DMSO and NMP solvents.

### 3.3. Comparison with DFT

In order to apply Equation (1) to estimate polymer refractive indices, the polarizability and density are required. Since the polarizability is assumed to be additive, the monomer polarizability ($\alpha_{DFT}$) has been used in a number of studies [26,34,73]. Although this does not sufficiently hold true at a theoretical level, for computational ease, all calculations were carried out only for the monomers terminated by hydrogen atoms. The second component of density typically requires molecular dynamics simulations but is computationally demanding. Hence, for computational ease, the density is estimated using a QSPR ($\rho_{QSPR}$) model. To evaluate the efficacy of this approach, polarizability calculations were carried out for a number of polymers for which experimental refractive indices recorded at different wavelengths (589, 633, 1324 nm) were available (see Table S13 in the Supplementary Materials) and spanned a range of 1.34–1.79. The higher values were included in particular to assess the ability of the $\alpha_{DFT}$-$\rho_{QSPR}$ driven approach to estimate the predicted reflective indices that are extrapolated by the model.

**Figure 7:**
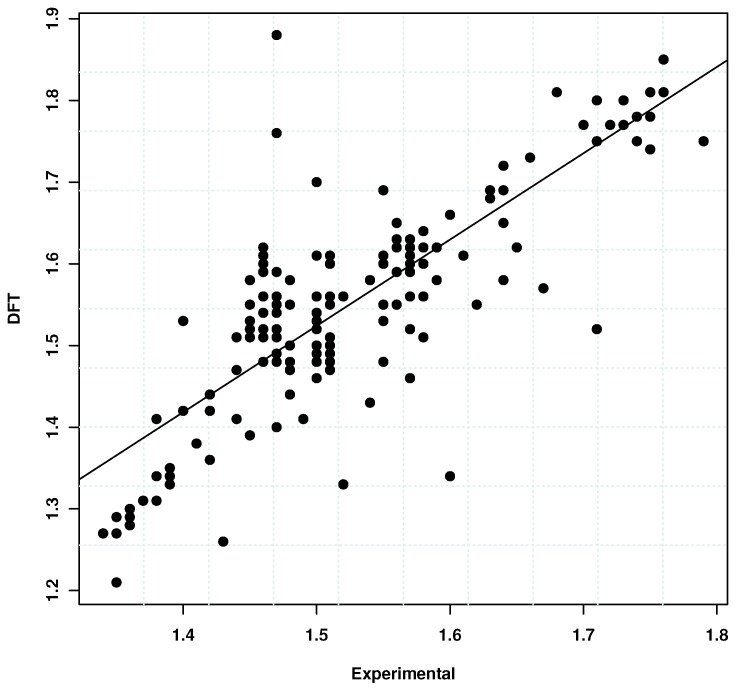
Plot shows the observed vs. the Density Functional Theory (DFT)-predicted refractive indices. The density in Equation (1) is calculated using a QSPR model. An overall correlation of 0.81 was obtained. See Table S13 in the Supplementary Materials for additional details.

Analysis of the results of 160 randomly selected polymers shows that, for most polymers studied, the absolute deviations are less than 0.10. Figure 7 shows the scatter plot of the experimental vs. calculated refractive indices. For this data, an overall correlation of 0.81 was obtained. The $\alpha_{DFT}$-$\rho_{QSPR}$ scheme in particular tends to overestimate *n* for nearly two-thirds of the samples with the average deviation around 0.07. Further examination of polymers with *n* > 1.71 (the maximum value in the calibration) shows that the $\alpha_{DFT}$-$\rho_{QSPR}$ based *n* estimations were again overestimated with errors ranging between 0.01 and 0.09. The deviations could be attributed to the density predictions, since the QSPR model was not found to have a significantly high performance. To investigate this further, only cases for which experimental refractive index and density data were available were considered. For a set of 51 polymers, the mean absolute deviations were around 0.15 with errors in the range −0.80–0.21 (see Table S14 in the Supplementary Materials). Here again, the $\alpha_{DFT}$-$\rho_{EXP}$ estimates were found to be larger than the experimental *n*. These results suggest that the errors in the polarizability estimates for the hydrogen-terminated monomer units could also be contributing to the errors. Given these results, caution must therefore exercised when comparing the QSPR predictions for *n* with the $\alpha_{DFT}$-$\rho_{QSPR}$ based estimates.

### 3.4. Analysis of Selected Monomers

**Figure 8:**
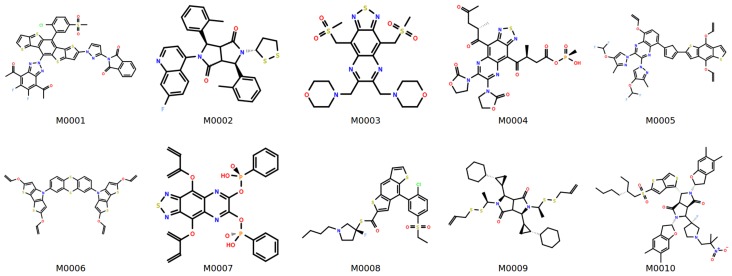
Monomers selected from de novo runs.

Table 4 summarizes the predicted properties for selected monomers shown in Figure 8. The structures are likely to show good thermal stability as seen from the glass transition temperatures that are around 200 ${}^{\circ }$C along with relatively high weight loss temperatures ($T_{d}$ > 350 ${}^{\circ }$C). The inclusion of groups such as cyclohexane have been shown to improve stability [73]. The predicted refractive indices are typically high and can be attributed to the presence of aromatic heterocycles and high sulfur content [74]. Substituents such as thioethers, thiazoles and nitro groups are also seen to increase refractive indices [67]. Analysis of the TDDFT calculations suggests that for a majority of the proposed structures, the absorption wavelengths peak at less than 300 nm (see plots in Figure S5 in the Supplementary Materials), and we expect these to have good optical transparency. Comparison of the QSPR and DFT-calculated refractive index estimates shows that—for the monomers: M0002, M0003, M0006, M0008, M0010—the deviation is not significantly high. The high deviation with respect to M0001 clearly suggests that there are limits to the extent of extrapolation that can be performed using the existing model. Table S15 in the Supplementary Materials list additional cases where there is a significant discrepancy between the QSPR and DFT predictions. Abbe numbers (listed in Table 4) for monomers M0002, M0009 and M0010 are relatively high, which should correspond to low wavelength dispersion. Birefringence depends on a number of factors such as the preferred orientations of the polymer chains, as well as the polarizability and van der Waals volume of the repeating units [75]. While low values are desirable, for the selected monomers, the calculated birefringence (δn) is somewhat high. For a few cases, negative birefringences are observed. We attribute this to largely to the incorrect estimations of the DFT-calculated polarizabilities. Methyl-terminated structures in place of the standard hydrogen have been shown to improve the accuracy [26] and therefore could be used.

## 4. Conclusions

Herein, a series of QSPR models are employed, which, in combination with a Darwinian evolution based search algorithm, facilitates the discovery of novel polymers that are able to satisfy several complementary properties. The designed monomers are generally seen to be synthesizable with polymerization for some candidates requiring specific conditions such as elevated temperatures or the presence of metal catalysts. For some monomers, the calculated birefringences were found to be high or negative. While low values are desirable, for the selected monomers, the calculated birefringence (δn) is somewhat high. Birefringence depends on a number of factors such as the preferred orientations of the polymer chains, as well as the polarizability and van der Waals volume of the repeating units [75]. For a few cases, negative birefringences were observed. We attribute some of this largely to the incorrect estimations of the DFT-calculated polarizabilities. Methyl-terminated structures in place of the standard hydrogen have been shown to improve the accuracy [26] and may help to address the issue. Alternatively, methods such as copolymerization of monomers with different birefringences, or the addition of small birefringent crystals, may also be employed [76].

Although the models are sufficiently predictive, there still exist inherent discrepancies between property estimations and the experimental values. Incorporating information relating to the experimental uncertainties in combination with nonlinear methods such as kernel-based PLS regression [77] may help to address some of these issues. Since experimental data are somewhat limited and model extrapolation is not always reliable, future work will focus on methods such as semi-supervised learning [78] that aim to build better predictive models using unlabelled data as additional data. Performing DFT calculations at a high level theory for the numerous candidates produced is still a computational bottleneck. It is hoped that methods such as deep learning can help approximate such calculations in shorter timeframes [79].

## Figures and Tables

**Table 1:** Summary of the experimental data available for refractive index (*n*), density (ρ), glass transition temperatures ($T_{g}$) and decomposition temperatures ($T_{d}$ for 10% weight loss). $N_{obs}$ is the number of available samples, while $N_{cal}$ and $N_{test}$ are the respective numbers in the calibration and test sets (based on a random 50:50 split of the data).

Property	$N_{\mathrm{obs}}$	Range	$N_{\mathrm{cal}}$	$N_{\mathrm{test}}$
*n*	237	1.34–1.71	120	117
ρ	195	0.84–2.1	99	96
$T_{g}$ (${}^{\circ }$C)	601	−143–399	304	297
$T_{d}$ (${}^{\circ }$C)	175	125–563	90	85

**Table 2:** Summary of the regression model performances for the refractive index (*n*), glass transition temperatures ($T_{g}$) and decomposition temperatures ($T_{d}$). Here, MAE is the mean absolute error, RMSE is the root mean squared error and $R^{2}$ the squared correlation between the observed and predicted values.

Model	Property	Calibration		Testing	
		$R_{\mathrm{cv}}^{2}$	RMSE(MAE)	$R^{2}$	RMSE(MAE)
PLSR	*n*	0.79	0.04 (0.03)	0.79	0.04 (0.03)
	$T_{g}$ (${}^{\circ }$C)	0.81	52 (34)	0.83	49 (38)
	$T_{d}$ (${}^{\circ }$C)	0.61	49 (24)	0.62	51 (41)
RF	*n*	0.83	0.03 (0.01)	0.88	0.03 (0.02)
	$T_{g}$ (${}^{\circ }$C)	0.86	44 (14)	0.88	40 (30)
	$T_{d}$ (${}^{\circ }$C)	0.80	35 (12)	0.72	45 (30)
	ρ	0.64	0.13 (0.04)	0.66	0.14 (0.08)

**Table 3:** Summary of the random forest classification performances for the polymer solubilities in different solvents. See Tables S7–S12 in the Supplementary Materials for performances with respect to each solvent. The 10-fold cross-validated $\kappa_{Cal}$ and $\kappa_{Test}$ values are reported for each solvent. Here, S, soluble, PS, partially soluble/swelling/soluble on heating and I, insoluble.

Solvent	#Samples	I	PS	S	$\kappa_{\mathrm{Cal}}$	$\kappa_{\mathrm{Test}}$
CHCl${}_{3}$	136	53	34	48	0.56	0.50
NMP	145	10	42	93	0.62	0.36
DMAc	105	8	41	56	0.52	0.48
DMSO	154	19	56	79	0.53	0.58
THF	120	15	59	46	0.49	0.62

**Table 4:** Summary of the calculated properties for selected monomers. For the refractive index $n_{pred}$, $\rho_{pred}$, $T_{g}$ and $T_{d}$, the prediction uncertainties are also provided. $n_{DFT}$ is the refractive index calculated according to Equation (1) with polarizabilities obtained from DFT. The Abbe number $v_{d}$ is calculated according to Equation (2) and makes use of the DFT-calculated polarizabilities and QSPR based density estimation. Absorption maxima $\lambda_{max}$ (in chloroform solvent) are calculated using Time-dependent Density Functional Theory (TD-DFT). MW, molecular weight.

Structure	MW	$n_{\mathrm{pred}}$	$T_{g}$	$T_{d}$	$\rho_{\mathrm{pred}}$	$n_{\mathrm{DFT}}$	$v_{d}$	Δn	$\lambda_{\max}$
M0001	927	1.98 ± 0.11	256 ± 27	438 ± 65	1.35 ± 0.23	1.79	7.79	0.09	367
M0002	570	1.75 ± 0.05	226 ± 62	456 ± 56	1.37 ± 0.32	1.72	22.85	0.07	356
M0003	571	1.74 ± 0.15	210 ± 51	398 ± 84	1.29 ± 0.16	1.67	5.85	0.09	420
M0004	663	1.79 ± 0.10	242 ± 50	408 ± 65	1.36 ± 0.31	1.65	7.45	0.38	411
M0005	801	1.80 ± 0.04	222 ± 47	466 ± 50	1.36 ± 0.22	1.98	1.98	0.05	429
M0006	716	1.84 ± 0.06	206 ± 41	396 ± 74	1.27 ± 0.16	1.80	10.88	−0.12	299
M0007	637	1.78 ± 0.14	223 ± 42	439 ± 81	1.37 ± 0.25	1.76	3.49	−0.05	455
M0008	596	1.78 ± 0.09	180 ± 64	370 ± 87	1.33 ± 0.32	1.70	13.13	−0.03	347
M0009	649	1.72 ± 0.04	198 ± 85	387 ± 79	1.63 ± 0.44	1.90	33.36	0.03	257
M0010	935	1.77 ± 0.11	226 ± 38	428 ± 60	1.44 ± 0.35	1.73	24.80	−0.13	305

## Supplementary Materials

The following are available online at http://www.mdpi.com/2073-4360/10/1/103/s1. Table S1: Description of the structure variables used in the models; Tables S2–S12: Experimental (data taken from literature) and model predicted values for *n*, $T_{g}$, $T_{d}$, ρ and solubility; Table S13: Experimental and predicted *n* measured at given wavelengths for different polymers; Table S14: Experimental and predicted *n* using DFT-based polarizability estimates and $\rho_{QSPR}$; Table S15: Cases where large deviations between QSPR and DFT estimates for *n* are observed; Figure S1: Histogram of the predicted *n* for the designed monomers; Figure S2: Scatter plot of the molecular weights vs. the experimental *n*; Figure S3: Scatter plot of the molecular weights vs. the predicted *n* of designed monomers; Figure S4: Histogram of the synthetic accessibility scores for the designed monomers; Figure F5: Calculated UV-Vis spectra for different polymers.

## Abbreviations

The following abbreviations are used in this manuscript:

QC	Quantum Chemistry
QSPR	Quantitative Structure Property Relationship
ML	Machine Learning
DFT	Density Functional Theory
TD-DFT	Time-Dependent Density Functional Theory
