# Supplementary material for: Designing High-Refractive Index Polymers Using Materials Informatics
Source: Polymers (Basel). 2018 Jan 22;10(1):103. doi: 10.3390/polym10010103 (PMC6415069; doi:10.3390/polym10010103)
Supplement: Supplementary file 1 [file polymers-10-00103-s001.pdf]

Supplementary material for

Designing High-refractive Index Polymers using Materials Informatics

Vishwesh Venkatraman and Bjørn Kåre Alsberg

List of Tables

S1 List of molecular descriptors used in this study. 4

S2 Summary of the regression model performances for the refractive index (n), glass transition temperatures (T_g) and decomposition temperatures (T_d). Here, NVAR is the number of variables used used in the model, N_{LV} the number of latent variables in the PLSR model, $mtry$ the number of predictors sampled for splitting at each node in the RF model, MAE is the mean absolute error, $RMSE$ is the root mean squared error and R^2 the squared correlation between the observed and predicted values. . . . 8

S3 The table lists the experimental and predicted (± uncertainty) refractive indices for different polymers. Predictions for both PLSR and RF models are reported. 9

S4 The table lists the experimental and predicted (± uncertainty) glass transition temperatures for different monomers. Predictions for both PLSR and RF models are reported. 21

S5 The table lists the experimental and predicted (± uncertainty) 10% thermal decomposition temperatures for different polymers. Predictions for only the RF model are reported. 49

S6 The table lists the experimental and predicted (± uncertainty) densities for different polymers. Predictions for only the RF model are reported. The experimental values are taken from multiple references[23, 4, 34] 58

S7 Summary of the classification model performances for the polymer solubilities in different solvents (NMP, THF, $CHCl_3$, DMSO and DMAC). The solubility classes include: S - soluble, PS - partially soluble/swelling/soluble on heating and I - insoluble. Here, $mtry$ is the number of predictors sampled for splitting at each node in the RF model, while accuracy and kappa coefficient are used as the performance metrics. 68

S8 The table lists the experimental and predicted solubility classes (S - soluble, PS - partially soluble/swelling/soluble on heating, I - insoluble) for different polymers in $CHCl_3$. Predictions for the RF model are reported. 69

S9 The table lists the experimental and predicted solubility classes (S - soluble, PS - partially soluble/swelling/soluble on heating, I - insoluble) for different polymers in N-methylpyrrolidone (NMP). Predictions for the RF model are reported. 76

S10 The table lists the experimental and predicted solubility classes (S - soluble, PS - partially soluble/swelling/soluble on heating, I - insoluble) for different polymers in THF. Predictions for the RF model are reported. 84

S11 The table lists the experimental and predicted solubility classes (S - soluble, PS - partially soluble/swelling/soluble on heating, I - insoluble) for different polymers in N,N-Dimethylacetamide (DMAc). Predictions for the RF model are reported. 91

S12 The table lists the experimental and predicted solubility classes (S - soluble, PS - partially soluble/swelling/soluble on heating, I - insoluble) for different polymers in DMSO. Predictions for the RF model are reported. 97

S13 The table lists the experimental and predicted refractive indices measured at given wavelengths for different polymers. Refractive indices for polymers at wavelengths other than 589 nm were taken from multiple references [51, 21, 16, 8, 57, 55, 19, 35, 42, 24, 54]. Here, the the refractive indices are predicted using the polarizabilities derived from DFT calculations and densities predicted using a QSPR model. 105
S14 The table lists the experimental (n_{exp}) and predicted refractive indices (n_{pred}) for different polymers. The n were estimated using DFT-based polarizability values at 589 nm and reported experimental densities. 117
S15 Cases where large deviations between QSPR and DFT estimates for n are observed. . 121

List of Figures

S1 Plot shows the histogram of the predicted refractive indices for the different monomers emerging from the de novo runs. 113
S2 Plot shows the scatter plot of the molecular weights vs the experimental refractive indices (taken from literature) of various polymers. 114
S3 Plot shows the scatter plot of the molecular weights vs the predicted refractive indices of different monomers emerging from the de novo runs. 114
S4 Plot shows the histogram of the synthetic accessibility scores for the different monomers emerging from the *de novo* runs. 115
S5 Calculated UV-VIS spectra for different polymers (see main article). 116

Table S1: List of molecular descriptors used in this study.

Name	Description
CPSA_PPSA-1	partial positive surface area
CPSA_PPSA-2	partial positive surface area * total positive charge on the molecule
CPSA_PPSA-3	charge weighted partial positive surface area
CPSA_PNSA-1	partial negative surface area
CPSA_PNSA-2	partial negative surface area * total negative charge on the molecule
CPSA_PNSA-3	charge weighted partial negative surface area
CPSA_DPSA-1	difference of PPSA-1 and PNSA-1
CPSA_DPSA-2	difference of FPSA-2 and PNSA-2
CPSA_DPSA-3	difference of PPSA-3 and PNSA-3
CPSA_FPSA-1	PPSA-1/total molecular surface area
CPSA_FFSA-2	PPSA-2/total molecular surface area
CPSA_FPSA-3	PPSA-3/total molecular surface area
CPSA_FNSA-1	PNSA-1/total molecular surface area
CPSA_FNSA-2	PNSA-2/total molecular surface area
CPSA_FNSA-3	PNSA-3/total molecular surface area
CPSA_WPSA-1	PPSA-1 * total molecular surface area/1000
CPSA_WPSA-2	PPSA-2 * total molecular surface area /1000
CPSA_WPSA-3	PPSA-3 * total molecular surface area/1000
CPSA_WNSA-1	PNSA-1 * total molecular surface area /1000
CPSA_WNSA-2	PNSA-2 * total molecular surface area/1000
CPSA_WNSA-3	PNSA-3 * total molecular surface area/1000
CPSA_RPCG	relative positive charge
CPSA_RNCG	relative negative charge
CPSA_RPCS	relative positive charge surface area
CPSA_RNCS	relative negative charge surface area
CPSA_PPSA-4	additional CPSA descriptors [1]
CPSA_PPSA-5	additional CPSA descriptors
CPSA_PNSA-4	additional CPSA descriptors
CPSA_PNSA-5	additional CPSA descriptors
CPSA_SPMX	additional CPSA descriptors
CPSA_SNMX	additional CPSA descriptors
MOPAC_COSMO_AREA	Area of the solvent accessible surface
MOPAC_COSMO_VOLUME	Volume included in the COSMO surface
MOPAC_HOMO	Highest occupied molecular orbital
MOPAC_LUMO	Lowest unoccupied molecular orbital
MOPAC_HLGAP	Difference between HOMO-LUMO energies
MOPAC_HLFRACTION	ratio of HOMO/LUMO energies
MOPAC_CHARGE_DIPOLE	Dipole moment is calculated from the atomic charges
MOPAC_HYBRID_DIPOLE	hybrid dipole moment
MOPAC_TOTAL_DIPOLE	total dipole moment
MOPAC_HOF	Heat of formation
MOPAC_ABSOLUTE_HARDNESS	Parr and Pople absolute hardness
MOPAC_TOTAL_SOFTNESS	Inverse of the hardness
MOPAC_CORE-CORE_REPULSION	core-core repulsion energy
MOPAC_TOTAL_ENERGY	total energy
MOPAC_ELEC_NUC_ATTR	ELECTRON-NUCLEAR ATTRACTION
MOPAC_ELEC_ELEC_REPL	ELECTRON-ELECTRON REPULSION
MOPAC_ELEC_ENERGY	TOTAL OF ELECTRONIC AND NUCLEAR ENERGIES
MOPAC_RESONANCE_ENERGY	resonance energy
MOPAC_EXCHG_ENERGY	exchange energy
MOPAC_TOTAL_ELEC_INTRN	total electronic interaction
MOPAC_TOTAL_EPHIL_DELOC	total electrophilic delocalizability
MOPAC_TOTAL_NPHIL_DELOC	total neutrophilic delocalizability
MOPAC_TOTAL_SPOL	total self polarizability
MOPAC_ELECTROPHILICITY	electrophilicity
MOPAC_PIMX	Principal moments of inertia
MOPAC_PIMY	Principal moments of inertia
MOPAC_PIMZ	Principal moments of inertia
MOPAC_MW	Molecular Weight
MOPAC_FILLEDLEVELS	Number of filled levels
MOPAC_MIN_DNR	minimum nucleophilic delocalizability

Continued on next page

Table S1 – *Continued from previous page*

Name	Description
MOPAC_MIN_DER	minimum electrophilic delocalizability
MOPAC_MIN_SPOL	minimum self polarizability
MOPAC_MAX_DNR	maximum nucleophilic delocalizability
MOPAC_MAX_DER	maximum electrophilic delocalizability
MOPAC_MAX_SPOL	maximum self polarizability
MOPAC_AVG_POLARIZABILITY	average polarizability
MOPAC_POLARANISOTROPY	polar anisotropy
MOPAC_VIB_ENTHALPY	vibrational ENTHALPY
MOPAC_VIB_HEATCAP	vibrational HEATCAP
MOPAC_VIB_ENTROPY	vibrational ENTROPY
MOPAC_ROT_ENTHALPY	rotational ENTHALPY
MOPAC_ROT_HEATCAP	rotational HEATCAP
MOPAC_ROT_ENTROPY	rotational ENTROPY
MOPAC_TRANS_ENTHALPY	translational ENTHALPY
MOPAC_TRANS_HEATCAP	translational HEATCAP
MOPAC_TRANS_ENTROPY	translational ENTROPY
MOPAC_POL_ALPHA	ALPHA polarizability
MOPAC_POL_BETA	BETA polarizability
MOPAC_POL_GAMMA	GAMMA polarizability
CHG_MAXPOSCHG	maximum positive charge
CHG_MINNEGCHG	maximum negative charge
CHG_TOTABSCHG	Total absolute charge
CHG_TOTNEGCHG	Total negative charge
CHG_TOTPOSCHG	Total positive charge
CHG_TOTSQCHG	total squared charge
CHG_CHGPOL	charge polarization
CHG_LOCDIPOLEINDX	local dipole index
CHG_DP	second-order submolecular polarity Parameter
CHG_SPP	submolecular polarity Parameter
CHG_TEIATOMS	topological electronic index for atoms
CHG_TEIBONDS	topological electronic index for bonds
CHG_ECDCI	Electronic charge density connectivity index
GEOM_Wiener3D	3D Wiener index
GEOM_RadGyration	Radius of gyration
GEOM_InertialSF	inertial shape factor
GEOM_MolEccentricity	Molecular eccentricity
GEOM_Asphericity	Asphericity index
GEOM_Spherosity	Spherosity index
GEOM_Globularity	Globularity index
GEOM_Ovality	Ovality index
GRAPH_GEIGGE_PROPERTY	Graph energy from the weighted (charge,self-polarizability,delocalizability) adjacency matrix
Radial Distribution Function 1	weighted by charge, self-polarizability, delocalizability
Radial Distribution Function 1.1	weighted by charge, self-polarizability, delocalizability
Radial Distribution Function 1.2	weighted by charge, self-polarizability, delocalizability
Radial Distribution Function 1.3	weighted by charge, self-polarizability, delocalizability
Radial Distribution Function 1.4	weighted by charge, self-polarizability, delocalizability
Radial Distribution Function 1.5	weighted by charge, self-polarizability, delocalizability
Radial Distribution Function 1.6	weighted by charge, self-polarizability, delocalizability
Radial Distribution Function 1.7	weighted by charge, self-polarizability, delocalizability
Radial Distribution Function 1.8	weighted by charge, self-polarizability, delocalizability
Radial Distribution Function 1.9	weighted by charge, self-polarizability, delocalizability
Radial Distribution Function 2	weighted by charge, self-polarizability, delocalizability
Radial Distribution Function 2.1	weighted by charge, self-polarizability, delocalizability
Radial Distribution Function 2.2	weighted by charge, self-polarizability, delocalizability
Radial Distribution Function 2.3	weighted by charge, self-polarizability, delocalizability
Radial Distribution Function 2.4	weighted by charge, self-polarizability, delocalizability
Radial Distribution Function 2.5	weighted by charge, self-polarizability, delocalizability
Radial Distribution Function 2.6	weighted by charge, self-polarizability, delocalizability
Radial Distribution Function 2.7	weighted by charge, self-polarizability, delocalizability
Radial Distribution Function 2.8	weighted by charge, self-polarizability, delocalizability
Radial Distribution Function 2.9	weighted by charge, self-polarizability, delocalizability
Radial Distribution Function 3	weighted by charge, self-polarizability, delocalizability
Radial Distribution Function 3.1	weighted by charge, self-polarizability, delocalizability

Continued on next page

Table S1 – *Continued from previous page*

Name	Description
Radial Distribution Function 3.2	weighted by charge, self-polarizability, delocalizability
Radial Distribution Function 3.3	weighted by charge, self-polarizability, delocalizability
Radial Distribution Function 3.4	weighted by charge, self-polarizability, delocalizability
Radial Distribution Function 3.5	weighted by charge, self-polarizability, delocalizability
Radial Distribution Function 3.6	weighted by charge, self-polarizability, delocalizability
Radial Distribution Function 3.7	weighted by charge, self-polarizability, delocalizability
Radial Distribution Function 3.8	weighted by charge, self-polarizability, delocalizability
Radial Distribution Function 3.9	weighted by charge, self-polarizability, delocalizability
Radial Distribution Function 4	weighted by charge, self-polarizability, delocalizability
Radial Distribution Function 4.1	weighted by charge, self-polarizability, delocalizability
Radial Distribution Function 4.2	weighted by charge, self-polarizability, delocalizability
Radial Distribution Function 4.3	weighted by charge, self-polarizability, delocalizability
Radial Distribution Function 4.4	weighted by charge, self-polarizability, delocalizability
Radial Distribution Function 4.5	weighted by charge, self-polarizability, delocalizability
Radial Distribution Function 4.6	weighted by charge, self-polarizability, delocalizability
Radial Distribution Function 4.7	weighted by charge, self-polarizability, delocalizability
Radial Distribution Function 4.8	weighted by charge, self-polarizability, delocalizability
Radial Distribution Function 4.9	weighted by charge, self-polarizability, delocalizability
Radial Distribution Function 5	weighted by charge, self-polarizability, delocalizability
Radial Distribution Function 5.1	weighted by charge, self-polarizability, delocalizability
Radial Distribution Function 5.2	weighted by charge, self-polarizability, delocalizability
Radial Distribution Function 5.3	weighted by charge, self-polarizability, delocalizability
Radial Distribution Function 5.4	weighted by charge, self-polarizability, delocalizability
Radial Distribution Function 5.5	weighted by charge, self-polarizability, delocalizability
Radial Distribution Function 5.6	weighted by charge, self-polarizability, delocalizability
Radial Distribution Function 5.7	weighted by charge, self-polarizability, delocalizability
Radial Distribution Function 5.8	weighted by charge, self-polarizability, delocalizability
Radial Distribution Function 5.9	weighted by charge, self-polarizability, delocalizability
Radial Distribution Function 6	weighted by charge, self-polarizability, delocalizability
Radial Distribution Function 6.1	weighted by charge, self-polarizability, delocalizability
Radial Distribution Function 6.2	weighted by charge, self-polarizability, delocalizability
Radial Distribution Function 6.3	weighted by charge, self-polarizability, delocalizability
Radial Distribution Function 6.4	weighted by charge, self-polarizability, delocalizability
Radial Distribution Function 6.5	weighted by charge, self-polarizability, delocalizability
Radial Distribution Function 6.6	weighted by charge, self-polarizability, delocalizability
Radial Distribution Function 6.7	weighted by charge, self-polarizability, delocalizability
Radial Distribution Function 6.8	weighted by charge, self-polarizability, delocalizability
Radial Distribution Function 6.9	weighted by charge, self-polarizability, delocalizability
Radial Distribution Function 7	weighted by charge, self-polarizability, delocalizability
Radial Distribution Function 7.1	weighted by charge, self-polarizability, delocalizability
Radial Distribution Function 7.2	weighted by charge, self-polarizability, delocalizability
Radial Distribution Function 7.3	weighted by charge, self-polarizability, delocalizability
Radial Distribution Function 7.4	weighted by charge, self-polarizability, delocalizability
Radial Distribution Function 7.5	weighted by charge, self-polarizability, delocalizability
Radial Distribution Function 7.6	weighted by charge, self-polarizability, delocalizability
Radial Distribution Function 7.7	weighted by charge, self-polarizability, delocalizability
Radial Distribution Function 7.8	weighted by charge, self-polarizability, delocalizability
Radial Distribution Function 7.9	weighted by charge, self-polarizability, delocalizability
Radial Distribution Function 8	weighted by charge, self-polarizability, delocalizability
Radial Distribution Function 8.1	weighted by charge, self-polarizability, delocalizability
Radial Distribution Function 8.2	weighted by charge, self-polarizability, delocalizability
Radial Distribution Function 8.3	weighted by charge, self-polarizability, delocalizability
Radial Distribution Function 8.4	weighted by charge, self-polarizability, delocalizability
Radial Distribution Function 8.5	weighted by charge, self-polarizability, delocalizability
Radial Distribution Function 8.6	weighted by charge, self-polarizability, delocalizability
Radial Distribution Function 8.7	weighted by charge, self-polarizability, delocalizability
Radial Distribution Function 8.8	weighted by charge, self-polarizability, delocalizability
Radial Distribution Function 8.9	weighted by charge, self-polarizability, delocalizability
Radial Distribution Function 9	weighted by charge, self-polarizability, delocalizability
Radial Distribution Function 9.1	weighted by charge, self-polarizability, delocalizability
Radial Distribution Function 9.2	weighted by charge, self-polarizability, delocalizability
Radial Distribution Function 9.3	weighted by charge, self-polarizability, delocalizability
Radial Distribution Function 9.4	weighted by charge, self-polarizability, delocalizability

Continued on next page

Table S1 – *Continued from previous page*

Name	Description
Radial Distribution Function 9.5	weighted by charge, self-polarizability, delocalizability
Radial Distribution Function 9.6	weighted by charge, self-polarizability, delocalizability
Radial Distribution Function 9.7	weighted by charge, self-polarizability, delocalizability
Radial Distribution Function 9.8	weighted by charge, self-polarizability, delocalizability
Radial Distribution Function 9.9	weighted by charge, self-polarizability, delocalizability
Radial Distribution Function 10	weighted by charge, self-polarizability, delocalizability
Radial Distribution Function 10.1	weighted by charge, self-polarizability, delocalizability
Radial Distribution Function 10.2	weighted by charge, self-polarizability, delocalizability
Radial Distribution Function 10.3	weighted by charge, self-polarizability, delocalizability
Radial Distribution Function 10.4	weighted by charge, self-polarizability, delocalizability
Radial Distribution Function 10.5	weighted by charge, self-polarizability, delocalizability
Radial Distribution Function 10.6	weighted by charge, self-polarizability, delocalizability
Radial Distribution Function 10.7	weighted by charge, self-polarizability, delocalizability
Radial Distribution Function 10.8	weighted by charge, self-polarizability, delocalizability
Radial Distribution Function 10.9	weighted by charge, self-polarizability, delocalizability
Radial Distribution Function 11	weighted by charge, self-polarizability, delocalizability
Radial Distribution Function 11.1	weighted by charge, self-polarizability, delocalizability
Radial Distribution Function 11.2	weighted by charge, self-polarizability, delocalizability
Radial Distribution Function 11.3	weighted by charge, self-polarizability, delocalizability
Radial Distribution Function 11.4	weighted by charge, self-polarizability, delocalizability
Radial Distribution Function 11.5	weighted by charge, self-polarizability, delocalizability
Radial Distribution Function 11.6	weighted by charge, self-polarizability, delocalizability
Radial Distribution Function 11.7	weighted by charge, self-polarizability, delocalizability
Radial Distribution Function 11.8	weighted by charge, self-polarizability, delocalizability
Radial Distribution Function 11.9	weighted by charge, self-polarizability, delocalizability
Radial Distribution Function 12	weighted by charge, self-polarizability, delocalizability
Radial Distribution Function 12.1	weighted by charge, self-polarizability, delocalizability
Radial Distribution Function 12.2	weighted by charge, self-polarizability, delocalizability
Radial Distribution Function 12.3	weighted by charge, self-polarizability, delocalizability
Radial Distribution Function 12.4	weighted by charge, self-polarizability, delocalizability
Radial Distribution Function 12.5	weighted by charge, self-polarizability, delocalizability
Radial Distribution Function 12.6	weighted by charge, self-polarizability, delocalizability
Radial Distribution Function 12.7	weighted by charge, self-polarizability, delocalizability
Radial Distribution Function 12.8	weighted by charge, self-polarizability, delocalizability
Radial Distribution Function 12.9	weighted by charge, self-polarizability, delocalizability
Radial Distribution Function 13	weighted by charge, self-polarizability, delocalizability
Radial Distribution Function 13.1	weighted by charge, self-polarizability, delocalizability
Radial Distribution Function 13.2	weighted by charge, self-polarizability, delocalizability
Radial Distribution Function 13.3	weighted by charge, self-polarizability, delocalizability
Radial Distribution Function 13.4	weighted by charge, self-polarizability, delocalizability
Radial Distribution Function 13.5	weighted by charge, self-polarizability, delocalizability
Radial Distribution Function 13.6	weighted by charge, self-polarizability, delocalizability
Radial Distribution Function 13.7	weighted by charge, self-polarizability, delocalizability
Radial Distribution Function 13.8	weighted by charge, self-polarizability, delocalizability
Radial Distribution Function 13.9	weighted by charge, self-polarizability, delocalizability
Radial Distribution Function 14	weighted by charge, self-polarizability, delocalizability
Radial Distribution Function 14.1	weighted by charge, self-polarizability, delocalizability
Radial Distribution Function 14.2	weighted by charge, self-polarizability, delocalizability
Radial Distribution Function 14.3	weighted by charge, self-polarizability, delocalizability
Radial Distribution Function 14.4	weighted by charge, self-polarizability, delocalizability
Radial Distribution Function 14.5	weighted by charge, self-polarizability, delocalizability
Radial Distribution Function 14.6	weighted by charge, self-polarizability, delocalizability
Radial Distribution Function 14.7	weighted by charge, self-polarizability, delocalizability
Radial Distribution Function 14.8	weighted by charge, self-polarizability, delocalizability
Radial Distribution Function 14.9	weighted by charge, self-polarizability, delocalizability
Radial Distribution Function 15	weighted by charge, self-polarizability, delocalizability

Table S2: Summary of the regression model performances for the refractive index (n), glass transition temperatures (T_g) and decomposition temperatures (T_d). Here, NVAR is the number of variables used used in the model, N_{LV} the number of latent variables in the PLSR model, mtry the number of predictors sampled for splitting at each node in the RF model, MAE is the mean absolute error, $RMSE$ is the root mean squared error and R^2 the squared correlation between the observed and predicted values.

**Model**	**Property**	**NVAR**	$N_{LV}/mtry$	**Calibration**		**Testing**	
				R^2_{cv}	$RMSE$ (MAE)	R^2	$RMSE$ (MAE)
PLSR	n	61	4	0.79	0.04 (0.03)	0.79	0.04 (0.03)
	T_g (°C)	175	5	0.81	52 (34)	0.83	49 (38)
	T_d (°C)	218	4	0.61	49 (24)	0.62	51 (41)
RF	n	5	3	0.83	0.03 (0.01)	0.88	0.03 (0.02)
	T_g (°C)	16	4	0.86	44 (14)	0.88	40 (30)
	T_d (°C)	11	4	0.80	35 (12)	0.72	45 (30)
	ρ	7	3	0.64	0.13 (0.04)	0.66	0.14 (0.08)

Table S3: The table lists the experimental and predicted (± uncertainty) refractive indices for different polymers. Predictions for both PLSR and RF models are reported.

MONOMER	n^{exp}	n^{pred}_{PLSR}	n^{pred}_{RF}	Ref
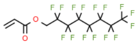	1.34	1.33 ± 0.02	1.36 ± 0.04	[6, 14, 4]
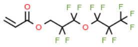	1.35	1.36 ± 0.02	1.37 ± 0.03	[6, 14, 4]
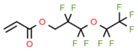	1.35	1.37 ± 0.02	1.36 ± 0.03	[6, 14, 4]
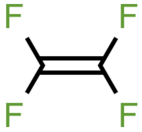	1.35	1.44 ± 0.05	1.4 ± 0.05	[6, 14, 4]
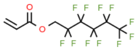	1.36	1.36 ± 0.02	1.36 ± 0.03	[6, 14, 4]
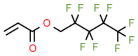	1.36	1.38 ± 0.01	1.37 ± 0.03	[6, 14, 4]
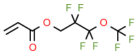	1.36	1.39 ± 0.02	1.37 ± 0.03	[6, 14, 4]
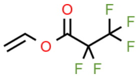	1.36	1.41 ± 0.01	1.39 ± 0.05	[6, 14, 4]
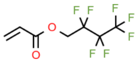	1.37	1.39 ± 0.01	1.37 ± 0.03	[6, 14, 4]
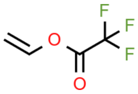	1.38	1.42 ± 0.01	1.39 ± 0.04	[6, 14, 4]
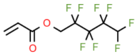	1.38	1.4 ± 0.01	1.38 ± 0.03	[6, 14, 4]
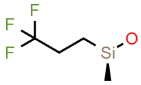	1.38	1.38 ± 0.02	1.39 ± 0.03	[6, 14, 4]
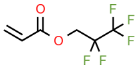	1.39	1.4 ± 0.01	1.38 ± 0.03	[6, 14, 4]
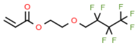	1.39	1.38 ± 0.02	1.37 ± 0.04	[6, 14, 4]
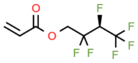	1.39	1.4 ± 0.01	1.4 ± 0.03	[6, 14, 4]
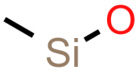	1.4	1.35 ± 0.04	1.43 ± 0.06	[6, 14, 4]
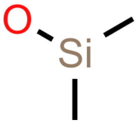	1.4	1.44 ± 0.03	1.42 ± 0.05	[6, 14, 4]
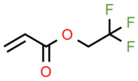	1.41	1.42 ± 0.01	1.41 ± 0.04	[6, 14, 4]
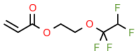	1.41	1.42 ± 0.01	1.41 ± 0.03	[6, 14, 4]
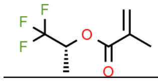	1.42	1.45 ± 0.01	1.42 ± 0.03	[6, 14, 4]

Continued on next page

Table S3 – *Continued from previous page*

MONOMER	n^{exp}	n^{pred}_{PLSR}	n^{pred}_{RF}	Ref
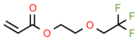	1.42	1.42 ± 0.01	1.41 ± 0.03	[6, 14, 4]
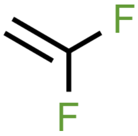	1.42	1.48 ± 0.02	1.46 ± 0.07	[6, 14, 4]
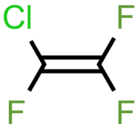	1.43	1.44 ± 0.05	1.46 ± 0.06	[6, 14, 4]
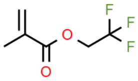	1.44	1.44 ± 0.01	1.42 ± 0.03	[6, 14, 4]
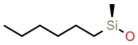	1.44	1.46 ± 0.02	1.45 ± 0.03	[6, 14, 4]
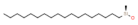	1.44	1.47 ± 0.02	1.46 ± 0.03	[6, 14, 4]
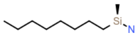	1.45	1.5 ± 0.02	1.48 ± 0.03	[6, 14, 4]
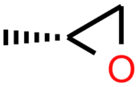	1.45	1.42 ± 0.02	1.46 ± 0.03	[6, 14, 4]
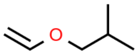	1.45	1.5 ± 0.01	1.46 ± 0.03	[6, 14, 4]
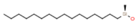	1.45	1.45 ± 0.02	1.46 ± 0.03	[6, 14, 4]
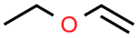	1.45	1.49 ± 0.01	1.46 ± 0.03	[6, 14, 4]
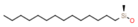	1.46	1.46 ± 0.02	1.46 ± 0.03	[6, 14, 4]
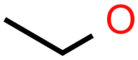	1.46	1.41 ± 0.03	1.46 ± 0.04	[6, 14, 4]
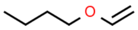	1.46	1.48 ± 0.01	1.46 ± 0.02	[6, 14, 4]
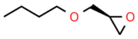	1.46	1.44 ± 0.01	1.47 ± 0.03	[6, 14, 4]
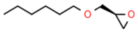	1.46	1.43 ± 0.01	1.47 ± 0.03	[6, 14, 4]
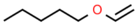	1.46	1.48 ± 0.01	1.46 ± 0.02	[6, 14, 4]
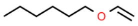	1.46	1.48 ± 0.01	1.46 ± 0.02	[6, 14, 4]
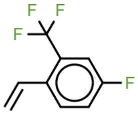	1.46	1.5 ± 0.02	1.52 ± 0.09	[6, 14, 4]
	1.46	1.48 ± 0.01	1.46 ± 0.02	[6, 14, 4]
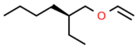	1.46	1.53 ± 0.01	1.46 ± 0.02	[6, 14, 4]
	1.46	1.49 ± 0.01	1.47 ± 0.03	[6, 14, 4]
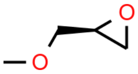	1.46	1.42 ± 0.02	1.47 ± 0.04	[6, 14, 4]
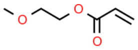	1.46	1.49 ± 0.01	1.48 ± 0.03	[6, 14, 4]
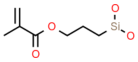	1.46	1.44 ± 0.02	1.45 ± 0.05	[6, 14, 4]
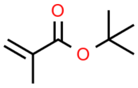	1.46	1.53 ± 0.01	1.51 ± 0.04	[6, 14, 4]
	1.46	1.49 ± 0.01	1.48 ± 0.03	[6, 14, 4]

Continued on next page

Table S3 – *Continued from previous page*

MONOMER	n^{exp}	n^{pred}_{PLSR}	n^{pred}_{RF}	Ref
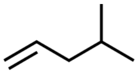	1.47	1.52 ± 0.02	1.48 ± 0.03	[6, 14, 4]
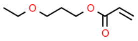	1.47	1.48 ± 0.04	1.48 ± 0.02	[6, 14, 4]
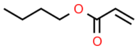	1.47	1.49 ± 0.01	1.47 ± 0.03	[6, 14, 4]
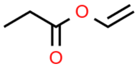	1.47	1.5 ± 0.01	1.47 ± 0.03	[6, 14, 4]
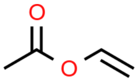	1.47	1.49 ± 0.01	1.47 ± 0.03	[6, 14, 4]
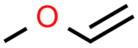	1.47	1.5 ± 0.01	1.47 ± 0.02	[6, 14, 4]
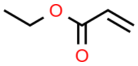	1.47	1.5 ± 0.01	1.48 ± 0.04	[6, 14, 4]
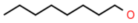	1.47	1.43 ± 0.02	1.46 ± 0.02	[6, 14, 4]
	1.47	1.49 ± 0.02	1.49 ± 0.03	[6, 14, 4]
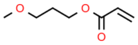	1.47	1.48 ± 0.02	1.47 ± 0.02	[6, 14, 4]
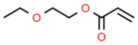	1.47	1.5 ± 0.01	1.47 ± 0.01	[6, 14, 4]
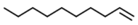	1.47	1.49 ± 0.02	1.47 ± 0.01	[6, 14, 4]
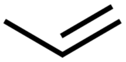	1.47	1.51 ± 0.02	1.49 ± 0.03	[6, 14, 4]
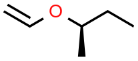	1.47	1.53 ± 0.01	1.49 ± 0.06	[6, 14, 4]
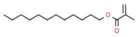	1.47	1.5 ± 0.01	1.48 ± 0.02	[6, 14, 4]
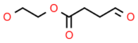	1.47	1.45 ± 0.01	1.44 ± 0.05	[6, 14, 4]
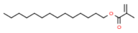	1.47	1.5 ± 0.01	1.48 ± 0.02	[6, 14, 4]
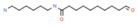	1.48	1.54 ± 0.02	1.49 ± 0.03	[6, 14, 4]
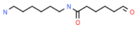	1.48	1.52 ± 0.01	1.51 ± 0.03	[6, 14, 4]
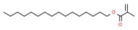	1.48	1.5 ± 0.02	1.47 ± 0.03	[6, 14, 4]
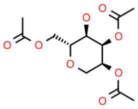	1.48	1.44 ± 0.01	1.47 ± 0.04	[6, 14, 4]
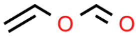	1.48	1.49 ± 0.02	1.47 ± 0.03	[6, 14, 4]
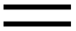	1.48	1.48 ± 0.04	1.51 ± 0.04	[6, 14, 4]
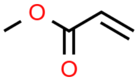	1.48	1.49 ± 0.02	1.48 ± 0.04	[6, 14, 4]
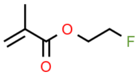	1.48	1.49 ± 0.01	1.46 ± 0.04	[6, 14, 4]
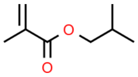	1.48	1.52 ± 0.01	1.5 ± 0.03	[6, 14, 4]
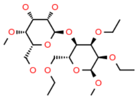	1.48	1.46 ± 0.02	1.47 ± 0.03	[6, 14, 4]
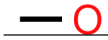	1.48	1.35 ± 0.06	1.46 ± 0.04	[6, 14, 4]

Continued on next page

Table S3 – *Continued from previous page*

MONOMER	n^{exp}	n^{pred}_{PLSR}	n^{pred}_{RF}	Ref
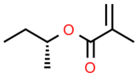	1.48	1.52 ± 0.01	1.49 ± 0.02	[6, 14, 4]
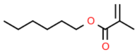	1.48	1.5 ± 0.01	1.49 ± 0.02	[6, 14, 4]
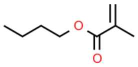	1.48	1.49 ± 0.01	1.5 ± 0.02	[6, 14, 4]
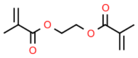	1.48	1.5 ± 0.06	1.49 ± 0.03	[6, 14, 4]
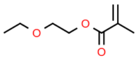	1.48	1.51 ± 0.01	1.48 ± 0.02	[6, 14, 4]
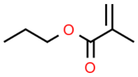	1.48	1.5 ± 0.01	1.49 ± 0.03	[6, 14, 4]
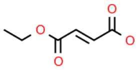	1.48	1.47 ± 0.02	1.48 ± 0.05	[6, 14, 4]
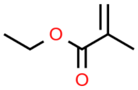	1.49	1.51 ± 0.01	1.5 ± 0.03	[6, 14, 4]
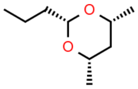	1.49	1.46 ± 0.02	1.48 ± 0.02	[6, 14, 4]
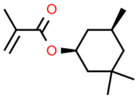	1.49	1.53 ± 0.01	1.5 ± 0.03	[6, 14, 4]
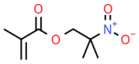	1.49	1.53 ± 0.01	1.5 ± 0.03	[6, 14, 4]
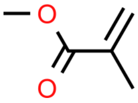	1.49	1.51 ± 0.01	1.5 ± 0.03	[6, 14, 4]
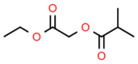	1.49	1.44 ± 0.01	1.45 ± 0.04	[6, 14, 4]
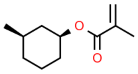	1.49	1.52 ± 0.01	1.5 ± 0.02	[6, 14, 4]
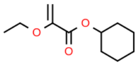	1.5	1.53 ± 0.01	1.49 ± 0.02	[6, 14, 4]
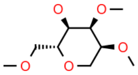	1.5	1.47 ± 0.02	1.45 ± 0.04	[6, 14, 4]
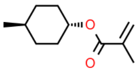	1.5	1.51 ± 0.01	1.5 ± 0.02	[6, 14, 4]
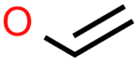	1.5	1.46 ± 0.03	1.49 ± 0.03	[6, 14, 4]
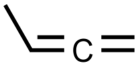	1.5	1.54 ± 0.02	1.5 ± 0.03	[6, 14, 4]
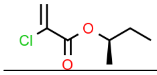	1.5	1.52 ± 0.01	1.52 ± 0.03	[6, 14, 4]

Continued on next page

Table S3 – *Continued from previous page*

MONOMER	n^{exp}	n^{pred}_{PLSR}	n^{pred}_{RF}	Ref
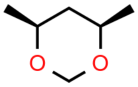	1.5	1.46 ± 0.02	1.48 ± 0.03	[6, 14, 4]
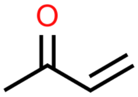	1.5	1.51 ± 0.01	1.5 ± 0.03	[6, 14, 4]
	1.5	1.5 ± 0.02	1.48 ± 0.03	[6, 14, 4]
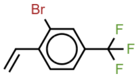	1.5	1.55 ± 0.01	1.52 ± 0.07	[6, 14, 4]
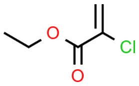	1.5	1.51 ± 0.01	1.51 ± 0.02	[6, 14, 4]
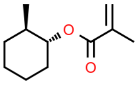	1.5	1.53 ± 0.01	1.49 ± 0.02	[6, 14, 4]
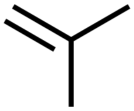	1.5	1.56 ± 0.02	1.5 ± 0.04	[6, 14, 4]
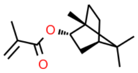	1.51	1.53 ± 0.01	1.51 ± 0.03	[6, 14, 4]
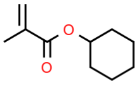	1.51	1.52 ± 0.01	1.51 ± 0.02	[6, 14, 4]
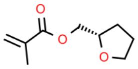	1.51	1.51 ± 0.01	1.49 ± 0.03	[6, 14, 4]
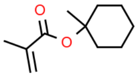	1.51	1.53 ± 0.01	1.52 ± 0.03	[6, 14, 4]
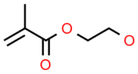	1.51	1.5 ± 0.01	1.48 ± 0.03	[6, 14, 4]
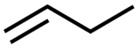	1.51	1.5 ± 0.02	1.5 ± 0.03	[6, 14, 4]
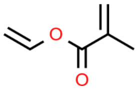	1.51	1.54 ± 0.01	1.54 ± 0.04	[6, 14, 4]
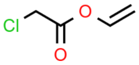	1.51	1.49 ± 0.01	1.52 ± 0.03	[6, 14, 4]
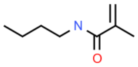	1.51	1.54 ± 0.01	1.52 ± 0.03	[6, 14, 4]
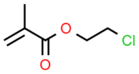	1.52	1.51 ± 0.01	1.5 ± 0.03	[6, 14, 4]
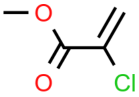	1.52	1.52 ± 0.01	1.51 ± 0.03	[6, 14, 4]
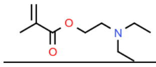	1.52	1.55 ± 0.01	1.57 ± 0.04	[6, 14, 4]

Continued on next page

Table S3 – *Continued from previous page*

MONOMER	n^{exp}	n^{pred}_{PLSR}	n^{pred}_{RF}	Ref
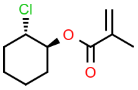	1.52	1.53 ± 0.01	1.48 ± 0.04	[6, 14, 4]
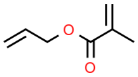	1.52	1.52 ± 0.01	1.52 ± 0.02	[6, 14, 4]
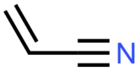	1.52	1.52 ± 0.02	1.51 ± 0.03	[6, 14, 4]
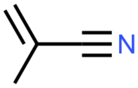	1.52	1.57 ± 0.02	1.52 ± 0.03	[6, 14, 4]
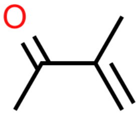	1.52	1.55 ± 0.01	1.52 ± 0.03	[6, 14, 4]
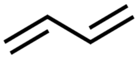	1.52	1.56 ± 0.02	1.57 ± 0.07	[6, 14, 4]
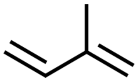	1.52	1.58 ± 0.02	1.55 ± 0.06	[6, 14, 4]
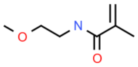	1.52	1.53 ± 0.01	1.53 ± 0.03	[6, 14, 4]
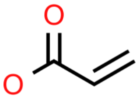	1.53	1.49 ± 0.01	1.5 ± 0.04	[6, 14, 4]
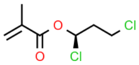	1.53	1.52 ± 0.01	1.51 ± 0.03	[6, 14, 4]
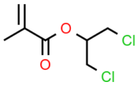	1.53	1.53 ± 0.02	1.51 ± 0.04	[6, 14, 4]
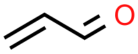	1.53	1.51 ± 0.02	1.52 ± 0.03	[6, 14, 4]
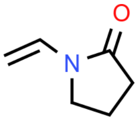	1.53	1.55 ± 0.01	1.55 ± 0.04	[6, 14, 4]
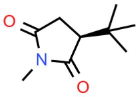	1.53	1.5 ± 0.01	1.48 ± 0.03	[6, 14, 4]
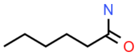	1.53	1.47 ± 0.01	1.51 ± 0.03	[6, 14, 4]
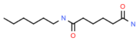	1.53	1.46 ± 0.02	1.49 ± 0.02	[6, 14, 4]
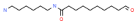	1.53	1.51 ± 0.01	1.51 ± 0.03	[6, 14, 4]
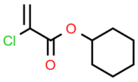	1.53	1.53 ± 0.01	1.52 ± 0.02	[6, 14, 4]
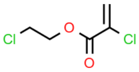	1.53	1.5 ± 0.01	1.52 ± 0.02	[6, 14, 4]
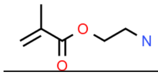	1.54	1.53 ± 0.01	1.53 ± 0.03	[6, 14, 4]

Continued on next page

Table S3 – *Continued from previous page*

MONOMER	n^{exp}	n^{pred}_{PLSR}	n^{pred}_{RF}	Ref
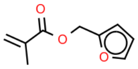	1.54	1.53 ± 0.01	1.57 ± 0.03	[6, 14, 4]
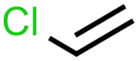	1.54	1.49 ± 0.02	1.52 ± 0.04	[6, 14, 4]
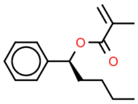	1.54	1.59 ± 0.01	1.57 ± 0.03	[6, 14, 4]
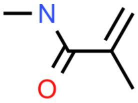	1.54	1.55 ± 0.01	1.53 ± 0.02	[6, 14, 4]
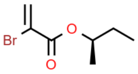	1.54	1.55 ± 0.01	1.52 ± 0.02	[6, 14, 4]
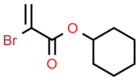	1.54	1.55 ± 0.01	1.53 ± 0.02	[6, 14, 4]
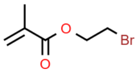	1.54	1.53 ± 0.01	1.51 ± 0.03	[6, 14, 4]
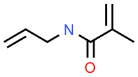	1.55	1.55 ± 0.01	1.54 ± 0.03	[6, 14, 4]
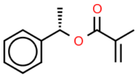	1.55	1.55 ± 0.01	1.56 ± 0.03	[6, 14, 4]
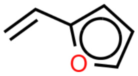	1.55	1.57 ± 0.01	1.57 ± 0.03	[6, 14, 4]
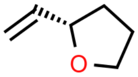	1.55	1.51 ± 0.01	1.5 ± 0.04	[6, 14, 4]
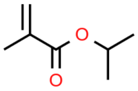	1.55	1.52 ± 0.01	1.53 ± 0.03	[6, 14, 4]
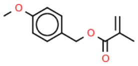	1.55	1.55 ± 0.01	1.57 ± 0.03	[6, 14, 4]
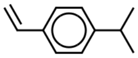	1.55	1.58 ± 0.02	1.57 ± 0.03	[6, 14, 4]
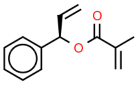	1.56	1.56 ± 0.01	1.57 ± 0.03	[6, 14, 4]
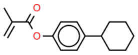	1.56	1.54 ± 0.01	1.57 ± 0.03	[6, 14, 4]
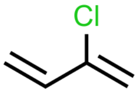	1.56	1.56 ± 0.02	1.57 ± 0.06	[6, 14, 4]
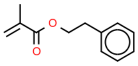	1.56	1.53 ± 0.01	1.57 ± 0.03	[6, 14, 4]
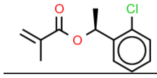	1.56	1.56 ± 0.01	1.58 ± 0.03	[6, 14, 4]

Continued on next page

Table S3 – *Continued from previous page*

MONOMER	n^{exp}	n^{pred}_{PLSR}	n^{pred}_{RF}	Ref
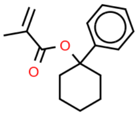	1.56	1.58 ± 0.01	1.56 ± 0.03	[6, 14, 4]
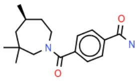	1.57	1.58 ± 0.01	1.57 ± 0.02	[6, 14, 4]
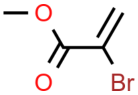	1.57	1.54 ± 0.01	1.52 ± 0.03	[6, 14, 4]
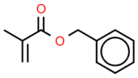	1.57	1.53 ± 0.01	1.57 ± 0.03	[6, 14, 4]
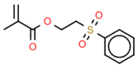	1.57	1.57 ± 0.06	1.55 ± 0.05	[6, 14, 4]
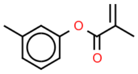	1.57	1.55 ± 0.01	1.57 ± 0.02	[6, 14, 4]
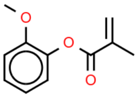	1.57	1.57 ± 0.01	1.57 ± 0.02	[6, 14, 4]
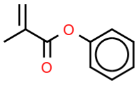	1.57	1.55 ± 0.01	1.57 ± 0.02	[6, 14, 4]
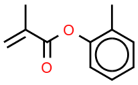	1.57	1.55 ± 0.01	1.57 ± 0.02	[6, 14, 4]
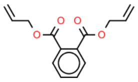	1.57	1.54 ± 0.01	1.57 ± 0.04	[6, 14, 4]
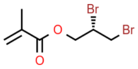	1.57	1.52 ± 0.01	1.51 ± 0.03	[6, 14, 4]
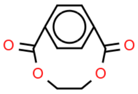	1.57	1.54 ± 0.02	1.56 ± 0.06	[6, 14, 4]
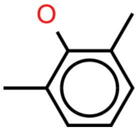	1.57	1.58 ± 0.02	1.58 ± 0.06	[6, 14, 4]
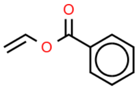	1.58	1.55 ± 0.01	1.58 ± 0.02	[6, 14, 4]
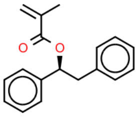	1.58	1.59 ± 0.01	1.58 ± 0.02	[6, 14, 4]
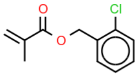	1.58	1.54 ± 0.01	1.58 ± 0.03	[6, 14, 4]
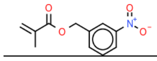	1.58	1.55 ± 0.01	1.56 ± 0.04	[6, 14, 4]

Continued on next page

**MONOMER**	n^{exp}	n^{pred}_{PLSR}	n^{pred}_{RF}	**Ref**
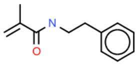	1.59	1.56 ± 0.01	1.58 ± 0.03	[6, 14, 4]
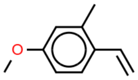	1.59	1.57 ± 0.01	1.6 ± 0.03	[6, 14, 4]
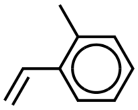	1.59	1.6 ± 0.02	1.59 ± 0.03	[6, 14, 4]
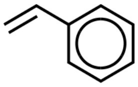	1.59	1.6 ± 0.02	1.6 ± 0.03	[6, 14, 4]
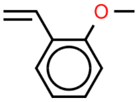	1.59	1.6 ± 0.01	1.59 ± 0.04	[6, 14, 4]
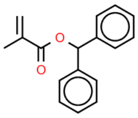	1.59	1.59 ± 0.02	1.58 ± 0.05	[6, 14, 4]
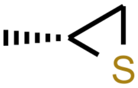	1.6	1.61 ± 0.02	1.57 ± 0.07	[6, 14, 4]
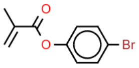	1.6	1.58 ± 0.01	1.57 ± 0.02	[6, 14, 4]
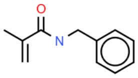	1.6	1.57 ± 0.01	1.57 ± 0.03	[6, 14, 4]
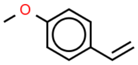	1.6	1.57 ± 0.01	1.6 ± 0.03	[6, 14, 4]
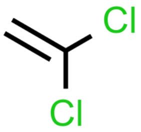	1.6	1.53 ± 0.03	1.57 ± 0.05	[6, 14, 4]
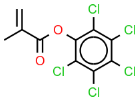	1.61	1.56 ± 0.01	1.59 ± 0.04	[6, 14, 4]
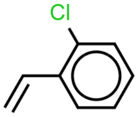	1.61	1.59 ± 0.02	1.6 ± 0.03	[6, 14, 4]
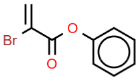	1.61	1.56 ± 0.01	1.59 ± 0.03	[6, 14, 4]
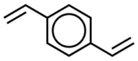	1.61	1.61 ± 0.02	1.62 ± 0.02	[6, 14, 4]
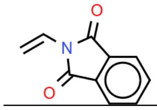	1.62	1.59 ± 0.02	1.62 ± 0.04	[6, 14, 4]

Continued on next page

Table S3 – *Continued from previous page*

MONOMER	n^{exp}	n^{pred}_{PLSR}	n^{pred}_{RF}	Ref
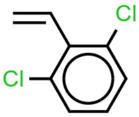	1.62	1.58 ± 0.02	1.61 ± 0.03	[6, 14, 4]
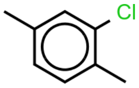	1.63	1.56 ± 0.02	1.59 ± 0.06	[6, 14, 4]
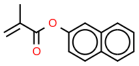	1.63	1.58 ± 0.01	1.62 ± 0.03	[6, 14, 4]
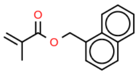	1.63	1.58 ± 0.01	1.62 ± 0.04	[6, 14, 4]
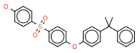	1.63	1.66 ± 0.06	1.62 ± 0.03	[6, 14, 4]
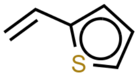	1.64	1.65 ± 0.02	1.62 ± 0.03	[6, 14, 4]
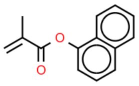	1.64	1.58 ± 0.01	1.63 ± 0.03	[6, 14, 4]
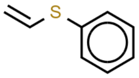	1.66	1.7 ± 0.02	1.65 ± 0.02	[6, 14, 4]
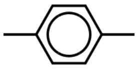	1.67	1.55 ± 0.02	1.6 ± 0.07	[6, 14, 4]
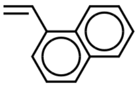	1.68	1.63 ± 0.02	1.67 ± 0.02	[6, 14, 4]
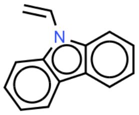	1.68	1.65 ± 0.01	1.67 ± 0.02	[6, 14, 4]
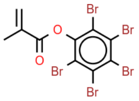	1.71	1.62 ± 0.01	1.6 ± 0.05	[6, 14, 4]
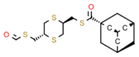	1.61	1.61 ± 0.02	1.63 ± 0.03	[60]
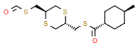	1.63	1.59 ± 0.02	1.61 ± 0.03	[60]
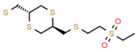	1.65	1.67 ± 0.05	1.65 ± 0.02	[60]
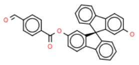	1.68	1.65 ± 0.01	1.68 ± 0.02	[45]
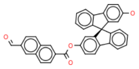	1.7	1.66 ± 0.01	1.68 ± 0.02	[45]
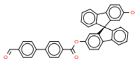	1.68	1.67 ± 0.02	1.68 ± 0.01	[45]
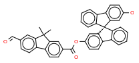	1.68	1.68 ± 0.01	1.68 ± 0.01	[45]
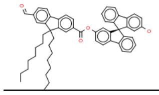	1.68	1.7 ± 0.02	1.67 ± 0.02	[45]

Continued on next page

Table S3 – *Continued from previous page*

MONOMER	n^{exp}	n^{pred}_{PLSR}	n^{pred}_{RF}	Ref
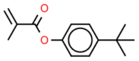	1.49	1.55 ± 0.01	1.56 ± 0.02	[29]
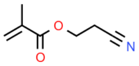	1.51	1.52 ± 0.01	1.51 ± 0.03	[29]
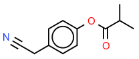	1.51	1.54 ± 0.01	1.57 ± 0.03	[29]
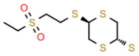	1.69	1.64 ± 0.06	1.64 ± 0.02	[42]
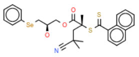	1.6	1.75 ± 0.03	1.67 ± 0.02	[46]
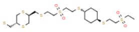	1.65	1.69 ± 0.09	1.64 ± 0.05	[46]
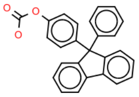	1.64	1.6 ± 0.02	1.63 ± 0.03	[46]
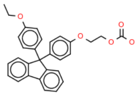	1.64	1.61 ± 0.01	1.63 ± 0.02	[46]
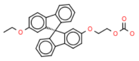	1.66	1.64 ± 0.02	1.66 ± 0.02	[46]
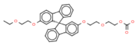	1.64	1.64 ± 0.01	1.65 ± 0.04	[46]
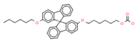	1.65	1.64 ± 0.01	1.65 ± 0.03	[46]
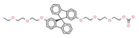	1.62	1.61 ± 0.02	1.65 ± 0.04	[42]
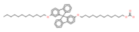	1.65	1.64 ± 0.02	1.65 ± 0.04	[42]
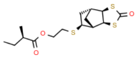	1.62	1.61 ± 0.01	1.62 ± 0.04	[42]
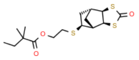	1.61	1.61 ± 0.02	1.61 ± 0.04	[42]
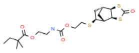	1.59	1.6 ± 0.02	1.61 ± 0.06	[50]
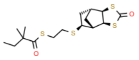	1.64	1.6 ± 0.02	1.63 ± 0.03	[50]
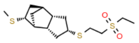	1.61	1.65 ± 0.05	1.64 ± 0.04	[12]
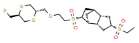	1.62	1.68 ± 0.1	1.65 ± 0.04	[12]
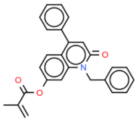	1.69	1.64 ± 0.01	1.66 ± 0.03	[12]
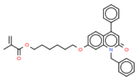	1.62	1.63 ± 0.01	1.62 ± 0.02	[12]
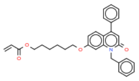	1.62	1.65 ± 0.01	1.62 ± 0.02	[12]
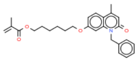	1.61	1.63 ± 0.01	1.61 ± 0.02	[12]
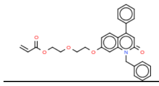	1.63	1.65 ± 0.01	1.62 ± 0.02	[12]

Continued on next page

Table S3 – *Continued from previous page*

**MONOMER**	n^{exp}	n^{pred}_{PLSR}	n^{pred}_{RF}	**Ref**
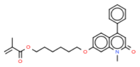	1.61	1.63 ± 0.01	1.63 ± 0.03	[44]
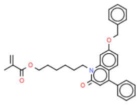	1.62	1.64 ± 0.01	1.63 ± 0.02	[44]
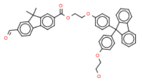	1.65	1.66 ± 0.01	1.65 ± 0.02	[44]
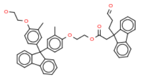	1.66	1.6 ± 0.01	1.63 ± 0.03	[44]
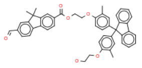	1.64	1.63 ± 0.01	1.65 ± 0.02	[44]
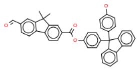	1.66	1.66 ± 0.01	1.67 ± 0.02	[44]
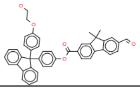	1.66	1.65 ± 0.01	1.66 ± 0.02	[44]

Table S4: The table lists the experimental and predicted (± uncertainty) glass transition temperatures for different monomers. Predictions for both PLSR and RF models are reported.

MONOMER	T_g^{exp}	$\widehat{T_g^{PLSR}}$	$\widehat{T_g^{RF}}$	Ref
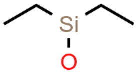	-143	-27± 32	15±76	[4]
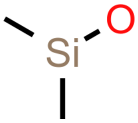	-121	-87± 30	1±79	[4]
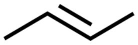	-102	-11± 10	-71±55	[4]
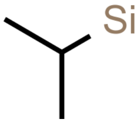	-100	-27± 16	-65±46	[4]
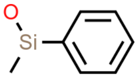	-86	-14± 21	-25±75	[4]
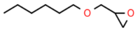	-85	-61± 14	-71±37	[4]
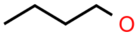	-83	-97± 15	-75±23	[4]
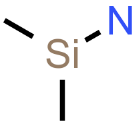	-82	-53± 31	-14±79	[4]
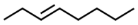	-81	-15± 12	-51±50	[4]
	-79	-31± 12	-46±36	[4]
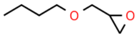	-79	-62± 13	-66±47	[4]
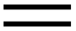	-78	-16± 26	-16±59	[4]
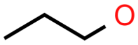	-78	-98± 17	-77±20	[4]
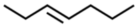	-77	-10± 11	-49±57	[4]
	-76	-31± 12	-40±41	[4]
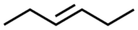	-76	-7± 10	-44±58	[4]
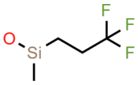	-74	-137± 35	13±81	[4]
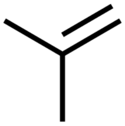	-74	9± 16	-59±65	[4]
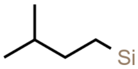	-70	-42± 13	-58±36	[4]
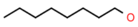	-70	-92± 16	-72±33	[4]
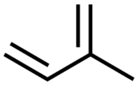	-70	57± 10	-40±61	[4]
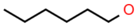	-69	-97± 15	-72±31	[4]
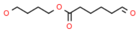	-68	-10± 14	-47±59	[4]
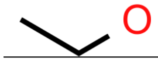	-67	-103± 27	-69±14	[4]

Continued on next page

**MONOMER**	T_g^{exp}	$\widehat{T_g^{PLSR}}$	$\widehat{T_g^{RF}}$	**Ref**
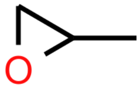	-67	-62± 19	-69±29	[4]
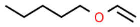	-66	-29± 12	-52±30	[4]
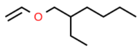	-66	20± 13	-33±60	[4]
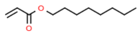	-65	-22± 10	-53±22	[4]
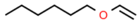	-64	-30± 11	-55±27	[4]
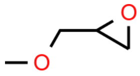	-62	-60± 12	-68±45	[4]
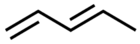	-60	59± 11	-14±69	[4]
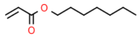	-60	-19± 10	-55±15	[4]
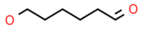	-60	-23± 11	-12±54	[4]
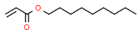	-58	-24± 11	-49±28	[4]
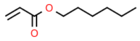	-57	-16± 9	-50±33	[4]
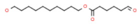	-56	-9± 19	-35±57	[4]
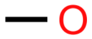	-55	-140± 49	-68±30	[4]
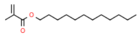	-55	-31± 15	-23±53	[4]
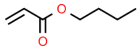	-54	-8± 7	-48±41	[4]
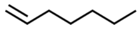	-53	-20± 11	-18±47	[4]
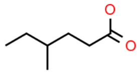	-53	-19± 9	-25±48	[4]
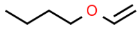	-52	-28± 13	-44±35	[4]
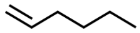	-50	-21± 10	-18±47	[4]
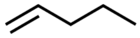	-50	-19± 12	-28±47	[4]
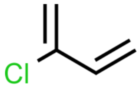	-48	80± 13	-38±62	[4]
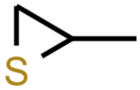	-47	19± 15	-35±66	[4]
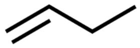	-45	-16± 15	-27±43	[4]
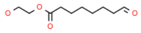	-45	-11± 15	-43±57	[4]
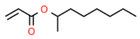	-45	-8± 11	-20±51	[4]
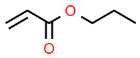	-44	-3± 7	-42±48	[4]
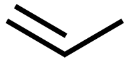	-7	-12± 20	-17±51	[4]
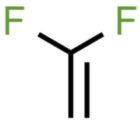	-40	33± 27	-28±58	[4]
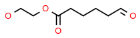	-40	-14± 13	-47±57	[4]
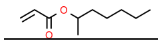	-38	2± 10	-26±47	[4]

Continued on next page

Table S4 – *Continued from previous page*

**MONOMER**	T_g^{exp}	$\widehat{T_g^{PLSR}}$	$\widehat{T_g^{RF}}$	**Ref**
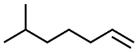	-34	-9± 10	-20±53	[4]
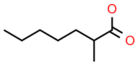	-33	-23± 9	-26±47	[4]
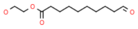	-30	-9± 16	-29±59	[4]
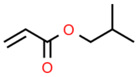	-24	8± 9	-36±38	[4]
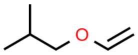	-22	-11± 9	-17±43	[4]
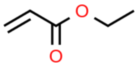	-22	10± 9	-25±62	[4]
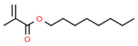	-20	-22± 11	-11±42	[4]
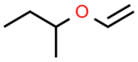	-20	6± 10	-15±52	[4]
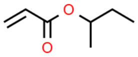	-20	14± 11	-18±48	[4]
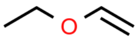	-19	-18± 12	-30±38	[4]
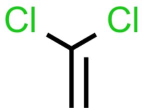	-17	-6± 33	-5±77	[4]
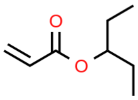	-16	35± 13	-6±58	[4]
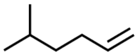	-14	-12± 11	2±65	[4]
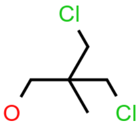	-8	-5± 14	30±75	[4]
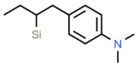	-6	67± 9	23±56	[4]
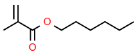	-5	-15± 9	-3±40	[4]
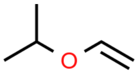	-3	-5± 10	-27±45	[4]
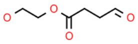	-1	-6± 13	-44±60	[4]
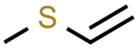	-1	32± 17	-2±72	[4]
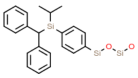	0	87± 42	120±64	[4]
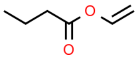	5	-12± 7	38±52	[4]
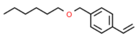	5	54± 9	40±34	[4]
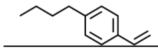	6	81± 8	35±45	[4]

Continued on next page

Table S4 – *Continued from previous page*

**MONOMER**	T_g^{exp}	$\widehat{T_g^{PLSR}}$	$\widehat{T_g^{RF}}$	**Ref**
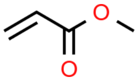	8	20± 10	2±64	[4]
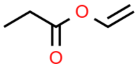	10	4± 8	22±46	[4]
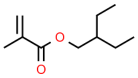	11	7± 10	26±42	[4]
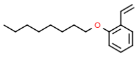	13	55± 9	32±32	[4]
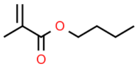	20	-10± 7	10±45	[4]
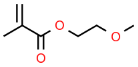	20	-6± 8	11±67	[4]
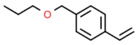	22	68± 8	34±31	[4]
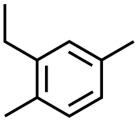	25	75± 10	68±45	[4]
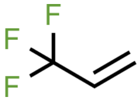	27	20± 13	-8±63	[4]
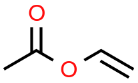	28	0± 11	24±55	[4]
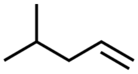	29	-2± 10	6±65	[4]
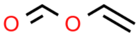	31	20± 18	32±69	[4]
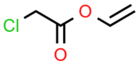	31	25± 9	30±50	[4]
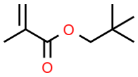	33	10± 9	42±51	[4]
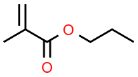	35	-5± 7	11±55	[4]
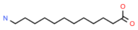	37	-18± 14	36±35	[4]
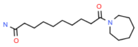	40	-18± 15	35±60	[4]
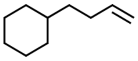	40	0± 12	46±75	[4]
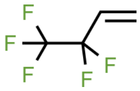	41	29± 14	27±61	[4]
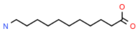	42	-17± 13	36±36	[4]
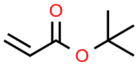	42	27± 11	24±60	[4]
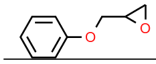	42	59± 7	53±42	[4]

Continued on next page

Table S4 – *Continued from previous page*

MONOMER	T_g^{exp}	$\widehat{T_g^{PLSR}}$	$\widehat{T_g^{RF}}$	Ref
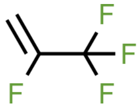	42	35± 13	43±41	[4]
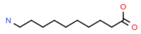	43	-14± 12	33±40	[4]
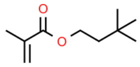	45	1± 9	34±51	[4]
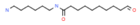	46	20± 16	34±44	[4]
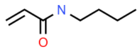	46	-2± 8	47±42	[4]
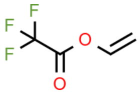	46	45± 14	54±56	[4]
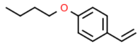	47	68± 8	54±33	[4]
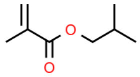	48	2± 8	33±41	[4]
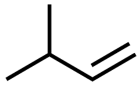	50	6± 13	23±63	[4]
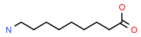	51	-18± 11	33±39	[4]
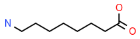	51	-16± 11	34±41	[4]
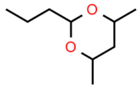	51	-37± 16	29±76	[4]
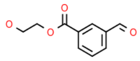	51	96± 9	74±77	[4]
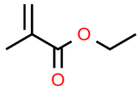	51	5± 7	27±54	[4]
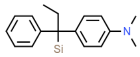	52	139± 14	69±53	[4]
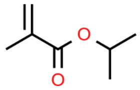	54	6± 9	44±35	[4]
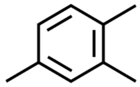	55	70± 10	75±48	[4]
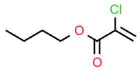	57	19± 9	61±41	[4]
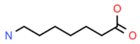	57	-14± 11	39±41	[4]
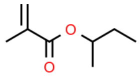	57	12± 10	53±43	[4]
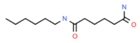	57	-10± 11	28±48	[4]
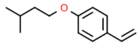	57	70± 8	54±28	[4]
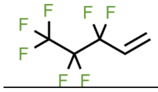	58	40± 18	13±59	[4]

Continued on next page

**MONOMER**	T_g^{exp}	$\widehat{T_g^{PLSR}}$	$\widehat{T_g^{RF}}$	**Ref**
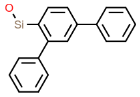	58	137± 13	89±42	[4]
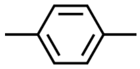	60	59± 10	64±50	[4]
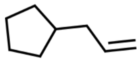	60	0± 10	31±63	[4]
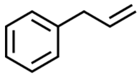	60	63± 7	64±36	[4]
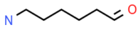	62	-13± 11	34±58	[4]
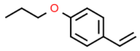	70	73± 8	60±27	[4]
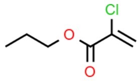	71	21± 8	64±38	[4]
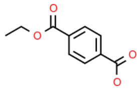	72	90± 10	112±80	[4]
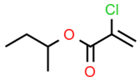	74	33± 9	60±45	[4]
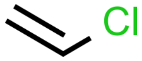	75	-3± 22	33±63	[4]
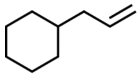	75	9± 11	50±75	[4]
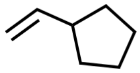	75	7± 12	4±67	[4]
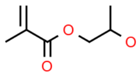	76	8± 7	47±58	[4]
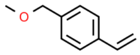	77	76± 7	66±48	[4]
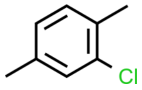	80	77± 9	62±62	[4]
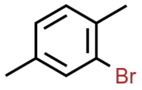	80	73± 10	76±60	[4]
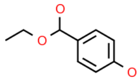	82	40± 7	50±57	[4]
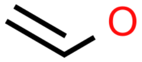	85	-18± 35	-30±64	[4]
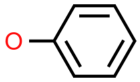	85	59± 13	53±66	[4]
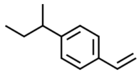	86	92± 9	61±51	[4]
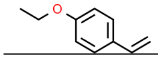	86	78± 8	70±36	[4]

Continued on next page

Table S4 – *Continued from previous page*

**MONOMER**	T_g^{exp}	$\widehat{T_g^{PLSR}}$	$\widehat{T_g^{RF}}$	**Ref**
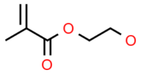	86	21± 8	47±59	[4]
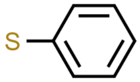	87	69± 9	74±69	[4]
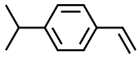	87	95± 8	80±44	[4]
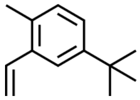	87	114± 13	69±43	[4]
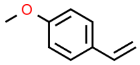	89	94± 8	90±28	[4]
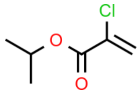	90	27± 9	54±48	[4]
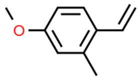	90	93± 7	85±43	[4]
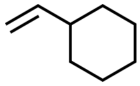	137	11± 14	80±71	[4]
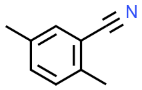	90	101± 19	102±46	[4]
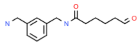	90	73± 11	82±46	[4]
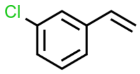	90	118± 10	114±32	[4]
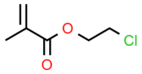	92	-1± 10	65±55	[4]
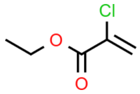	93	26± 9	54±53	[4]
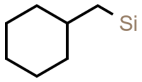	93	-21± 13	52±73	[4]
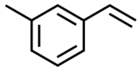	97	100± 11	107±36	[4]
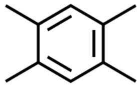	100	78± 11	90±43	[4]
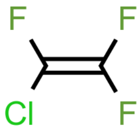	100	77± 12	76±54	[4]
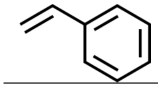	100	106± 11	104±40	[4]

Continued on next page

Table S4 – *Continued from previous page*

**MONOMER**	T_g^{exp}	$\widehat{T_g^{PLSR}}$	$\widehat{T_g^{RF}}$	**Ref**
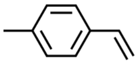	101	99± 9	100±33	[4]
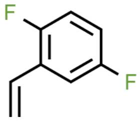	101	131± 10	126±41	[4]
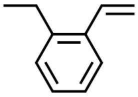	103	106± 12	107±38	[4]
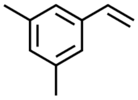	104	106± 11	105±41	[4]
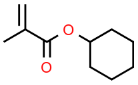	104	12± 10	89±48	[4]
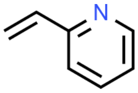	104	113± 11	104±26	[4]
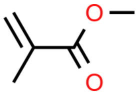	105	20± 8	70±64	[4]
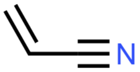	105	44± 24	14±62	[4]
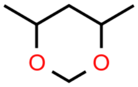	105	-43± 16	48±77	[4]
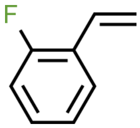	105	123± 11	124±41	[4]
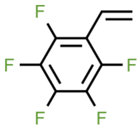	105	168± 14	192±77	[4]
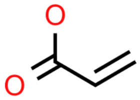	106	34± 14	56±69	[4]
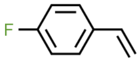	106	109± 9	112±32	[4]
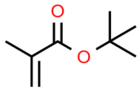	107	24± 11	86±44	[4]
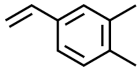	111	101± 9	101±34	[4]
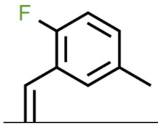	111	118± 10	113±38	[4]

Continued on next page

Table S4 – *Continued from previous page*

MONOMER	T_g^{exp}	$\widehat{T_g^{PLSR}}$	$\widehat{T_g^{RF}}$	Ref
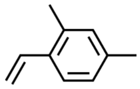	112	97± 10	105±41	[4]
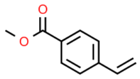	113	97± 8	90±40	[4]
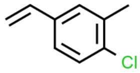	114	106± 8	120±51	[4]
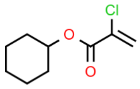	114	43± 11	61±46	[4]
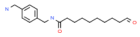	115	67± 12	95±38	[4]
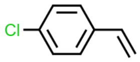	116	111± 11	123±32	[4]
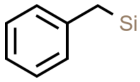	117	52± 10	25±67	[4]
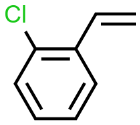	119	124± 11	122±31	[4]
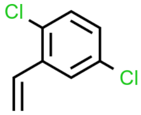	120	121± 10	124±30	[4]
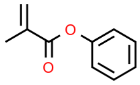	120	56± 8	104±36	[4]
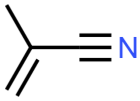	120	44± 20	25±66	[4]
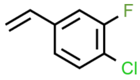	122	116± 9	124±31	[4]
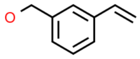	125	99± 11	109±41	[4]
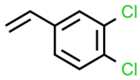	128	115± 10	120±31	[4]
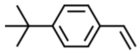	129	98± 9	76±47	[4]
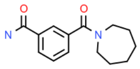	130	106± 12	113±41	[4]
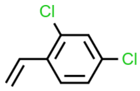	133	124± 11	128±33	[4]
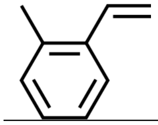	136	101± 11	126±34	[4]

Continued on next page

Table S4 – *Continued from previous page*

**MONOMER**	T_g^{exp}	$\widehat{T_g^{PLSR}}$	$\widehat{T_g^{RF}}$	**Ref**
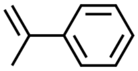	136	93± 9	112±38	[4]
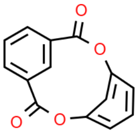	138	181± 18	139±66	[4]
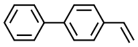	138	150± 11	135±42	[4]
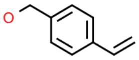	140	90± 8	100±45	[4]
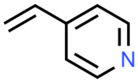	142	106± 11	126±37	[4]
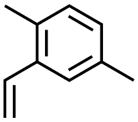	143	103± 11	118±40	[4]
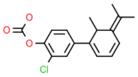	138	151± 12	115±54	[4]
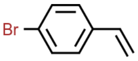	144	112± 10	125±33	[4]
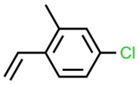	145	101± 9	123±48	[4]
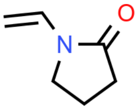	145	51± 13	33±62	[4]
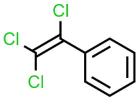	145	124± 12	141±35	[4]
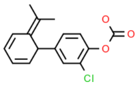	146	126± 13	111±58	[4]
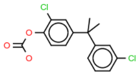	146	107± 9	116±39	[4]
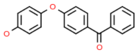	146	132± 8	157±61	[4]
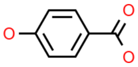	147	95± 11	124±53	[4]
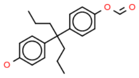	148	143± 15	134±51	[4]
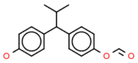	149	109± 10	117±63	[4]
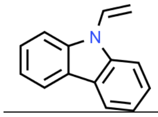	150	180± 14	141±45	[4]

Continued on next page

Table S4 – *Continued from previous page*

**MONOMER**	T_g^{exp}	$\widehat{T_g^{PLSR}}$	$\widehat{T_g^{RF}}$	**Ref**
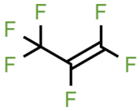	152	70± 18	126±62	[4]
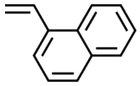	159	182± 13	144±30	[4]
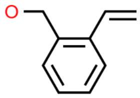	160	105± 12	116±35	[4]
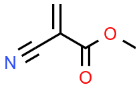	160	71± 21	104±69	[4]
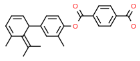	171	191± 14	257±99	[4]
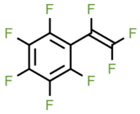	194	190± 19	214±96	[4]
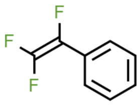	202	136± 12	169±53	[4]
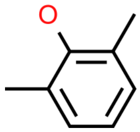	209	79± 11	89±56	[4]
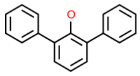	220	141± 9	178±74	[4]
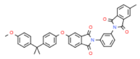	220	232± 13	248±69	[4]
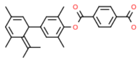	225	193± 13	257±94	[4]
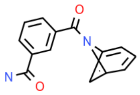	272	197± 20	197±98	[4]
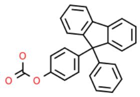	275	197± 20	190±89	[4]
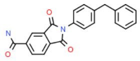	277	189± 10	296±58	[4]
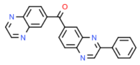	318	238± 17	299±62	[4]
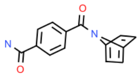	327	260± 59	192±106	[4]
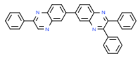	372	295± 21	332±68	[4]
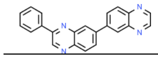	376	243± 17	306±82	[4]

Continued on next page

MONOMER	T_g^{exp}	$\widehat{T_g^{PLSR}}$	$\widehat{T_g^{RF}}$	Ref
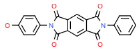	399	285± 13	360±61	[4]
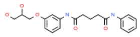	111	103± 10	147±92	[4]
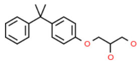	100	84± 9	89±36	[4]
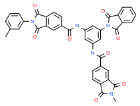	327	284± 23	333±25	[3]
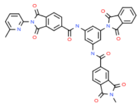	332	282± 22	332±23	[3]
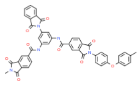	356	264± 37	332±35	[3]
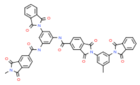	371	299± 32	334±29	[3]
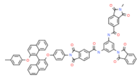	333	290± 37	331±19	[3]
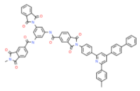	362	304± 42	336±32	[3]
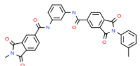	301	226± 25	317±40	[3]
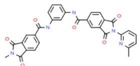	303	216± 25	315±44	[3]
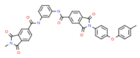	319	241± 30	318±34	[3]
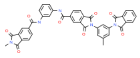	349	261± 28	339±34	[3]
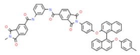	304	281± 33	323±33	[3]
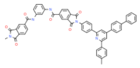	336	295± 41	328±35	[3]
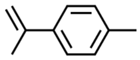	121	88± 8	97±44	[4]
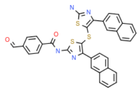	229	230± 17	226±22	[16]
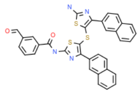	212	220± 18	225±26	[16]
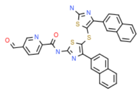	228	235± 20	230±28	[16]
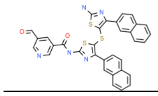	203	226± 20	213±27	[16]

Continued on next page

Table S4 – *Continued from previous page*

MONOMER	T_g^{exp}	$\widehat{T_g^{PLSR}}$	$\widehat{T_g^{RF}}$	Ref
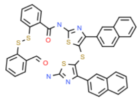	194	254± 46	238±38	[16]
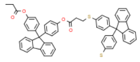	194	205± 33	193±34	[38]
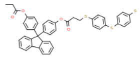	125	141± 19	165±48	[38]
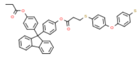	120	148± 17	181±53	[38]
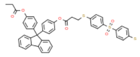	174	176± 19	214±33	[38]
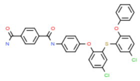	220	198± 11	216±30	[15]
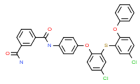	208	182± 12	211±40	[15]
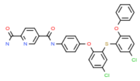	217	207± 11	216±38	[15]
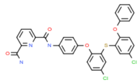	205	190± 12	217±34	[15]
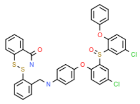	181	268± 28	196±25	[15]
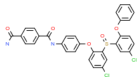	239	208± 18	206±26	[15]
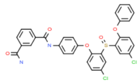	228	217± 14	205±29	[15]
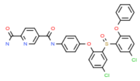	235	218± 12	216±29	[15]
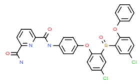	226	192± 16	218±24	[15]
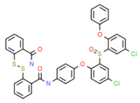	197	273± 29	200±28	[15]
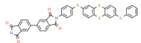	252	261± 11	243±21	[25]
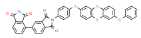	229	250± 10	233±22	[25]
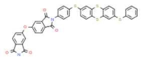	240	245± 11	232±23	[25]
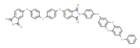	201	168± 17	211±31	[25]
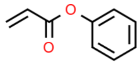	57	58± 9	75±63	[62]
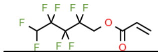	-35	45± 27	16±69	[62]

Continued on next page

Table S4 – *Continued from previous page*

**MONOMER**	T_g^{exp}	$\widehat{T_g^{PLSR}}$	$\widehat{T_g^{RF}}$	**Ref**
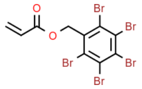	180	87± 25	138±93	[62]
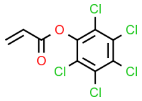	147	75± 15	129±55	[62]
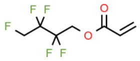	-26	48± 15	-9±47	[62]
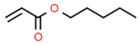	-57	-15± 8	-48±37	[62]
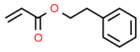	-3	32± 9	24±38	[62]
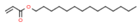	24	-37± 17	5±43	[62]
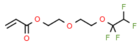	-40	-24± 24	-15±58	[62]
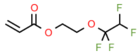	-22	-4± 23	5±55	[62]
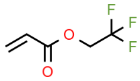	-10	23± 17	6±48	[62]
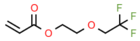	-38	-11± 21	8±53	[62]
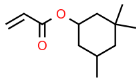	15	42± 13	24±44	[62]
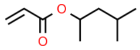	-15	19± 12	-25±39	[62]
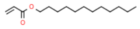	-3	-31± 14	-16±39	[62]
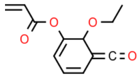	30	109± 18	86±64	[62]
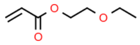	-50	-9± 8	-18±51	[62]
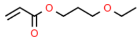	-55	-12± 9	-22±50	[62]
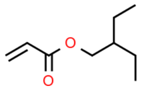	-50	10± 10	-36±46	[62]
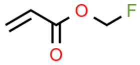	15	26± 11	18±55	[62]
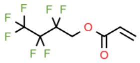	-30	43± 24	-8±52	[62]
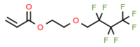	-45	6± 30	13±57	[62]
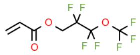	-55	33± 39	21±58	[62]
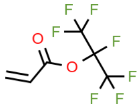	10	63± 33	11±53	[62]
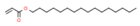	35	-42± 19	-18±47	[62]
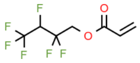	-22	24± 22	-19±50	[62]
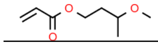	-56	1± 7	-38±43	[62]

Continued on next page

Table S4 – *Continued from previous page*

**MONOMER**	T_g^{exp}	$\widehat{T_g^{PLSR}}$	$\widehat{T_g^{RF}}$	**Ref**
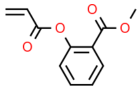	46	107± 12	86±52	[62]
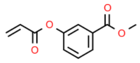	38	67± 10	68±51	[62]
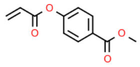	67	69± 10	73±50	[62]
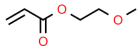	-50	-1± 8	-33±45	[62]
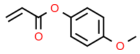	51	64± 9	86±54	[62]
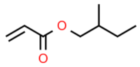	-32	7± 9	-28±27	[62]
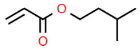	-45	-6± 8	-43±34	[62]
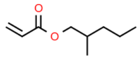	-38	6± 9	-34±40	[62]
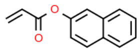	85	116± 9	96±48	[62]
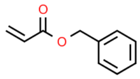	6	38± 7	28±44	[62]
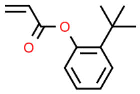	72	98± 14	89±36	[62]
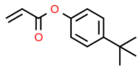	71	61± 7	75±29	[62]
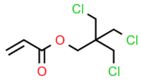	46	31± 16	23±60	[62]
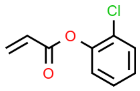	53	77± 9	66±34	[62]
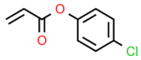	58	56± 12	78±48	[62]
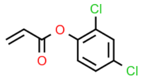	60	74± 8	72±43	[62]
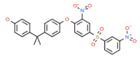	138	186± 26	234±26	[11]
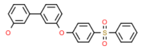	142	165± 13	201±43	[11]
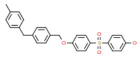	146	143± 12	189±47	[11]
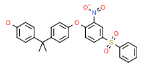	150	228± 17	208±28	[11]
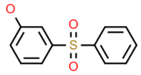	165	191± 21	100±64	[11]
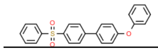	168	128± 25	194±43	[11]

Continued on next page

Table S4 – *Continued from previous page*

MONOMER	T_g^{exp}	$\widehat{T_g^{PLSR}}$	$\widehat{T_g^{RF}}$	**Ref**
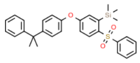	171	198± 31	178±34	[11]
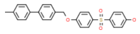	176	179± 12	184±56	[11]
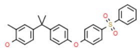	178	211± 13	186±50	[11]
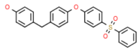	180	153± 15	196±45	[11]
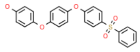	180	170± 11	202±46	[11]
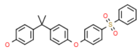	186	179± 15	195±42	[11]
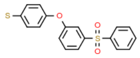	188	218± 28	186±51	[11]
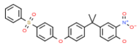	190	242± 15	210±33	[11]
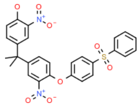	195	230± 24	226±25	[11]
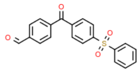	195	232± 18	207±37	[11]
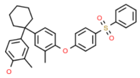	197	220± 16	185±36	[11]
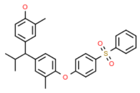	200	194± 13	194±28	[11]
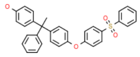	200	225± 14	200±30	[11]
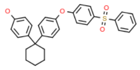	205	242± 14	201±34	[11]
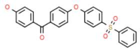	205	235± 18	204±29	[11]
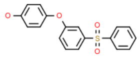	210	150± 18	181±56	[11]
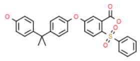	210	180± 12	200±40	[11]
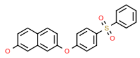	215	185± 10	200±51	[11]
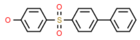	216	159± 15	180±65	[11]
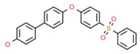	220	188± 11	194±44	[11]
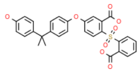	220	205± 16	213±21	[11]
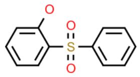	221	250± 41	112±65	[11]
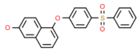	221	182± 10	200±47	[11]
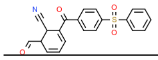	222	282± 39	223±29	[11]

Continued on next page

Table S4 – *Continued from previous page*

MONOMER	T_g^{exp}	$\widehat{T_g^{PLSR}}$	$\widehat{T_g^{RF}}$	Ref
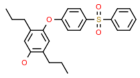	225	134± 15	194±45	[11]
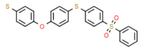	225	142± 21	205±38	[11]
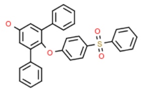	227	191± 19	210±36	[11]
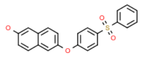	227	168± 12	200±50	[11]
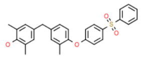	228	180± 12	187±48	[11]
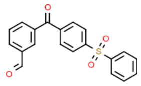	228	226± 15	207±41	[11]
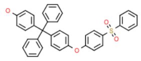	230	266± 21	208±33	[11]
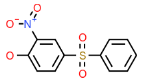	234	117± 26	206±64	[11]
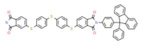	263	242± 19	244±27	[53]
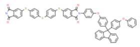	223	238± 25	234±27	[53]
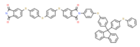	223	207± 21	230±37	[53]
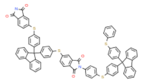	272	277± 31	251±31	[53]
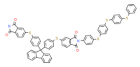	229	214± 21	234±34	[53]
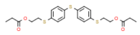	94	61± 21	117±40	[59]
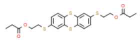	143	91± 27	134±30	[59]
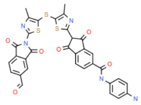	269	230± 16	250±22	[48]
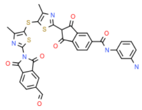	250	233± 14	252±13	[48]
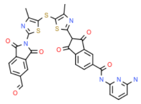	255	232± 15	251±18	[48]
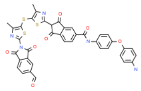	233	220± 17	241±17	[48]
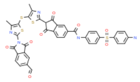	261	261± 17	253±19	[48]
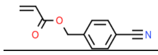	44	58± 16	81±52	[28]

Continued on next page

Table S4 – *Continued from previous page*

MONOMER	T_g^{exp}	$\widehat{T_g^{PLSR}}$	$\widehat{T_g^{RF}}$	Ref
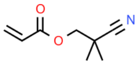	51	29± 17	42±53	[28]
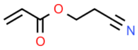	4	10± 16	15±62	[28]
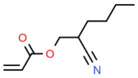	85	9± 17	79±57	[28]
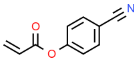	90	74± 19	78±52	[28]
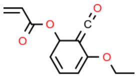	24	73± 18	51±66	[28]
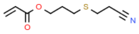	-58	-4± 20	15±73	[28]
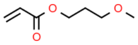	-75	-8± 8	-36±41	[28]
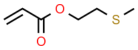	-60	12± 12	-30±58	[28]
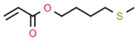	-70	-1± 12	-16±58	[28]
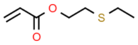	-71	-2± 12	-14±67	[28]
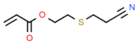	-50	4± 20	29±74	[28]
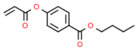	13	46± 11	80±55	[28]
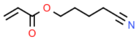	-40	-3± 16	-22±59	[28]
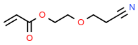	-23	-6± 17	-11±55	[28]
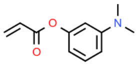	47	83± 10	90±55	[28]
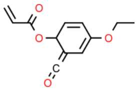	37	74± 16	52±49	[28]
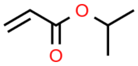	-3	11± 9	-24±47	[28]
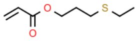	-76	-4± 13	-13±68	[28]
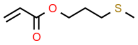	-65	4± 12	-25±52	[28]
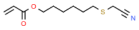	-59	-17± 20	14±72	[28]
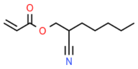	116	-1± 17	82±56	[28]
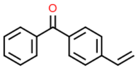	98	135± 8	100±36	[28]
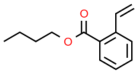	66	85± 6	86±29	[28]
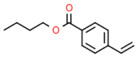	76	84± 7	81±36	[28]
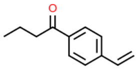	74	96± 7	73±35	[28]
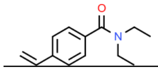	102	103± 9	96±28	[28]

Continued on next page

Table S4 – *Continued from previous page*

MONOMER	T_g^{exp}	$\widehat{T_g^{PLSR}}$	$\widehat{T_g^{RF}}$	Ref
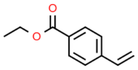	94	90± 8	84±38	[28]
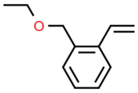	74	87± 9	74±29	[28]
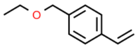	86	72± 7	49±43	[28]
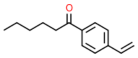	66	80± 8	69±28	[28]
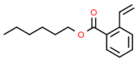	45	69± 7	79±28	[28]
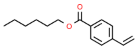	66	74± 8	77±33	[28]
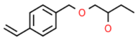	46	75± 8	54±39	[28]
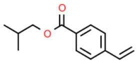	90	93± 6	91±43	[28]
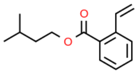	68	81± 6	81±28	[28]
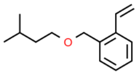	78	72± 7	70±32	[28]
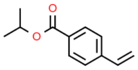	95	94± 7	78±35	[28]
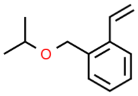	88	89± 9	80±25	[28]
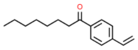	50	68± 10	72±22	[28]
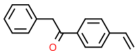	78	117± 8	108±54	[28]
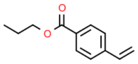	92	92± 7	85±20	[28]
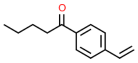	70	84± 7	72±36	[28]
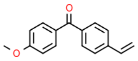	103	131± 8	109±41	[28]
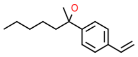	91	94± 13	91±34	[28]
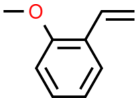	75	107± 10	94±42	[28]
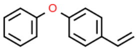	100	124± 9	115±52	[28]
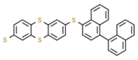	235	202± 17	208±51	[41]
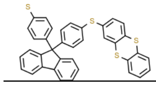	237	217± 23	185±56	[41]

Continued on next page

Table S4 – *Continued from previous page*

MONOMER	T_g^{exp}	$\widehat{T_g^{PLSR}}$	$\widehat{T_g^{RF}}$	Ref
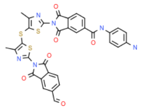	269	267± 15	261±17	[48]
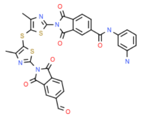	250	262± 14	254±15	[48]
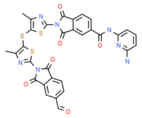	255	255± 16	252±14	[48]
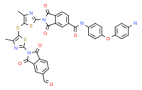	233	251± 15	244±25	[48]
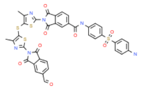	261	273± 17	260±21	[48]
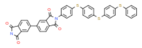	227	225± 13	234±17	[26]
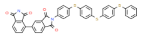	227	221± 11	233±24	[26]
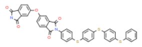	196	230± 16	234±23	[26]
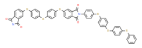	179	168± 18	211±31	[26]
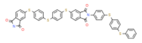	192	167± 15	210±35	[26]
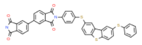	252	211± 13	249±16	[27]
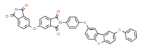	251	235± 10	246±16	[27]
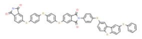	201	147± 21	204±27	[27]
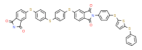	189	217± 20	189±23	[58]
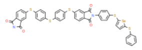	181	203± 18	190±27	[58]
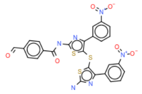	244	233± 25	245±23	[18]
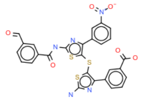	229	222± 22	231±23	[18]
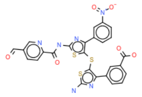	242	217± 24	243±20	[18]
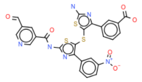	219	198± 24	227±20	[18]
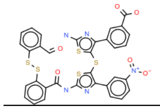	210	233± 31	229±28	[18]

Continued on next page

**MONOMER**	T_g^{exp}	$\widehat{T_g^{PLSR}}$	$\widehat{T_g^{RF}}$	**Ref**
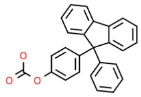	253	196± 20	188±91	[46]
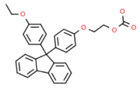	150	160± 17	169±92	[46]
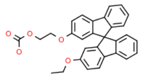	148	148± 26	120±81	[46]
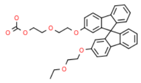	89	126± 26	124±73	[46]
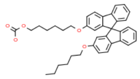	60	116± 26	91±52	[46]
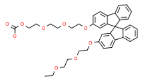	48	89± 29	125±60	[46]
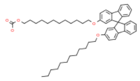	16	81± 39	106±56	[46]
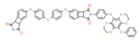	212	237± 78	200±40	[61]
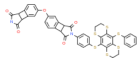	247	268± 79	215±41	[61]
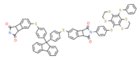	266	292± 82	224±41	[61]
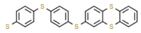	143	148± 20	144±46	[51]
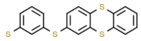	142	151± 17	127±44	[51]
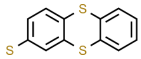	145	178± 18	133±30	[51]
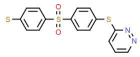	193	148± 18	187±29	[63]
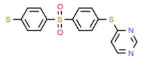	202	144± 20	197±32	[63]
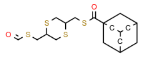	110	98± 31	107±53	[60]
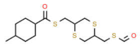	65	5± 35	60±47	[60]
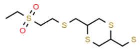	47	10± 56	58±48	[60]
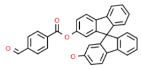	331	218± 23	300±74	[45]
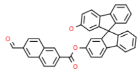	346	232± 23	325±52	[45]
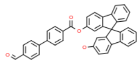	352	234± 25	288±72	[45]
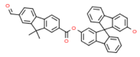	349	227± 23	322±64	[45]
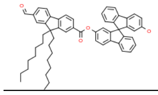	177	192± 36	203±46	[45]

Continued on next page

**MONOMER**	T_g^{exp}	$\widehat{T_g^{PLSR}}$	$\widehat{T_g^{RF}}$	**Ref**
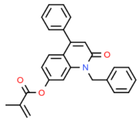	90	158± 16	125±84	[12]
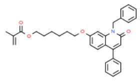	52	139± 15	171±93	[12]
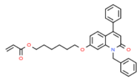	58	126± 16	151±88	[12]
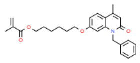	64	96± 17	89±52	[12]
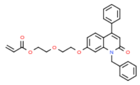	73	132± 14	180±107	[12]
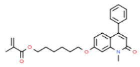	58	93± 15	124±91	[12]
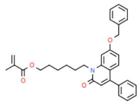	59	104± 16	168±92	[12]
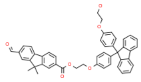	193	226± 21	200±51	[44]
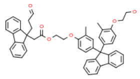	109	217± 30	187±47	[44]
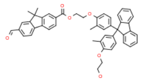	161	243± 23	184±43	[44]
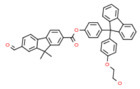	217	238± 19	215±69	[44]
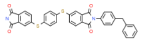	227	206± 10	234±19	[13]
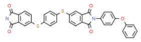	230	211± 11	232±20	[13]
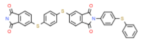	231	226± 11	230±27	[13]
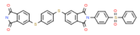	266	228± 21	235±22	[13]
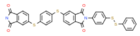	205	209± 16	236±26	[13]
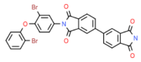	275	295± 11	287±31	[5]
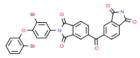	296	305± 13	283±30	[5]
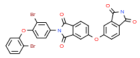	251	273± 12	282±40	[5]
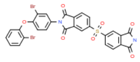	281	322± 16	271±41	[5]
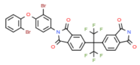	299	283± 24	283±43	[5]
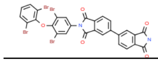	287	315± 15	286±30	[5]

Continued on next page

Table S4 – *Continued from previous page*

MONOMER	T_g^{exp}	$\widehat{T_g^{PLSR}}$	$\widehat{T_g^{RF}}$	Ref
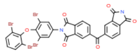	323	324± 17	287±38	[5]
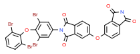	283	294± 14	278±37	[5]
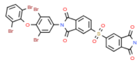	328	320± 21	289±47	[5]
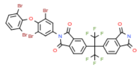	303	289± 23	285±40	[5]
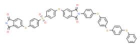	208	189± 20	214±20	[49]
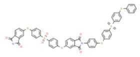	238	259± 86	249±38	[49]
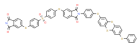	235	228± 19	224±24	[49]
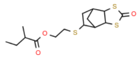	85	32± 28	89±37	[42]
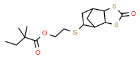	105	42± 28	100±48	[42]
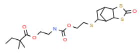	44	41± 28	99±70	[42]
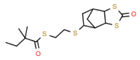	109	28± 35	93±28	[42]
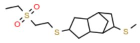	74	50± 46	82±55	[50]
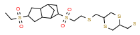	113	20± 85	109±69	[50]
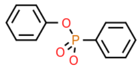	105	58± 23	95±29	[30]
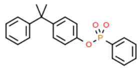	98	140± 14	109±30	[30]
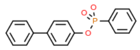	140	87± 27	126±37	[30]
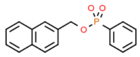	99	118± 22	110±30	[30]
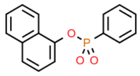	124	106± 24	121±38	[30]
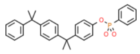	112	152± 22	139±59	[30]
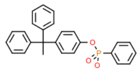	123	187± 12	166±48	[30]
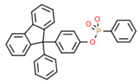	214	166± 34	199±42	[30]
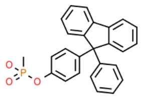	208	158± 39	164±55	[30]
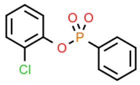	59	75± 20	75±42	[30]
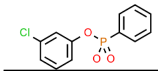	85	64± 21	88±48	[30]

Continued on next page

Table S4 – *Continued from previous page*

MONOMER	T_g^{exp}	$\widehat{T_g^{PLSR}}$	$\widehat{T_g^{RF}}$	Ref
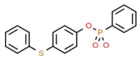	81	79± 24	130±50	[30]
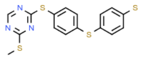	127	94± 17	121±61	[40]
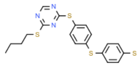	77	96± 17	98±52	[40]
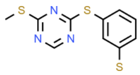	129	121± 13	123±36	[40]
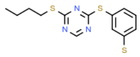	75	122± 16	98±42	[40]
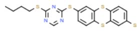	93	147± 19	105±56	[40]
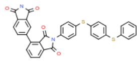	247	228± 11	238±20	[8]
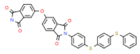	215	214± 10	224±19	[8]
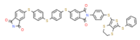	153	162± 18	176±32	[57]
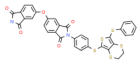	188	223± 15	230±33	[57]
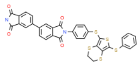	230	246± 15	242±29	[57]
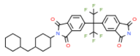	259	230± 24	247±35	[55]
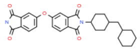	292	200± 18	272±53	[55]
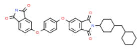	238	211± 19	246±28	[55]
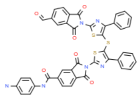	273	280± 20	256±19	[19]
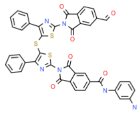	255	288± 17	256±18	[19]
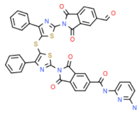	261	281± 18	258±14	[19]
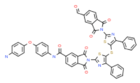	237	232± 34	263±32	[19]
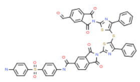	264	295± 25	263±22	[19]
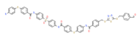	206	202± 24	245±36	[35]
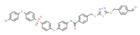	233	203± 21	233±26	[35]
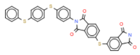	221	239± 12	233±37	[24]
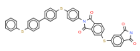	248	253± 12	248±30	[24]
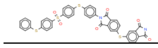	250	219± 14	243±20	[24]

Continued on next page

Table S4 – *Continued from previous page*

MONOMER	T_g^{exp}	$\widehat{T_g^{PLSR}}$	$\widehat{T_g^{RF}}$	Ref
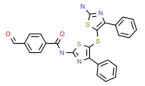	221	218± 17	228±46	[17]
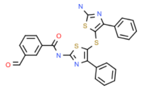	248	215± 18	215±45	[17]
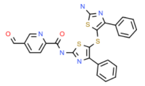	250	225± 18	245±36	[17]
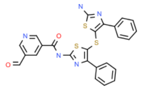	265	219± 19	245±39	[17]
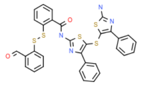	243	276± 29	228±45	[17]
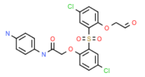	235	253± 25	207±33	[20]
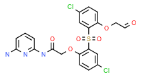	199	229± 17	203±33	[20]
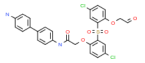	231	241± 15	210±28	[20]
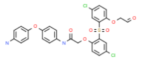	172	242± 19	194±28	[20]
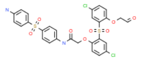	211	279± 23	213±23	[20]
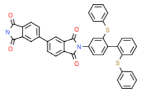	245	262± 12	247±14	[52]
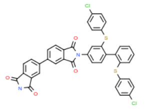	244	291± 19	245±25	[52]
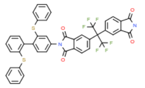	220	279± 20	240±25	[52]
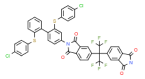	239	270± 28	248±34	[52]
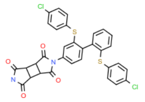	257	248± 28	230±38	[52]
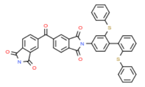	278	272± 14	269±27	[52]
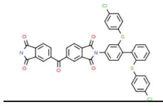	306	295± 19	259±32	[52]

Continued on next page

Table S4 – *Continued from previous page*

MONOMER	T_g^{exp}	$\widehat{T_g^{PLSR}}$	$\widehat{T_g^{RF}}$	Ref
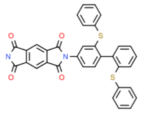	227	265± 15	235±17	[52]
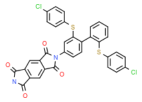	243	290± 21	245±39	[52]
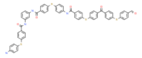	226	197± 26	237±21	[64]
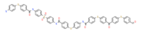	248	217± 34	253±19	[64]
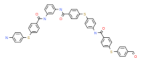	245	178± 26	241±22	[64]
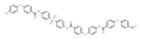	278	211± 21	261±28	[64]
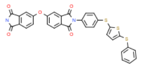	220	256± 13	224±19	[7]
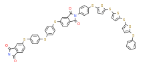	177	176± 29	189±25	[7]
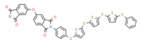	157	205± 25	223±35	[7]
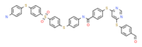	229	190± 14	223±16	[65]
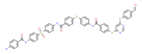	261	226± 19	254±28	[65]
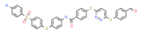	227	193± 15	228±16	[65]
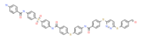	262	214± 20	259±30	[65]
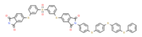	178	192± 15	192±32	[9]
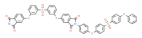	207	203± 30	242±33	[9]
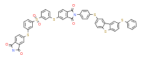	203	210± 19	218±35	[9]
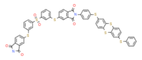	205	202± 20	207±20	[9]
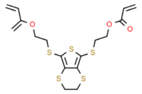	134	87± 28	119±58	[21]
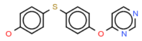	132	102± 12	122±39	[37]
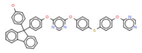	201	241± 24	193±46	[37]
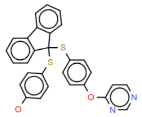	143	180± 24	192±63	[37]
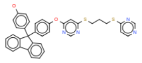	165	204± 22	177±43	[37]
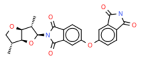	264	173± 14	244±56	[56]
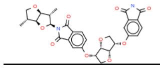	228	175± 24	221±29	[56]

Continued on next page

Table S4 – *Continued from previous page*

MONOMER	T_g^{exp}	$\widehat{T_g^{PLSR}}$	$\widehat{T_g^{RF}}$	**Ref**
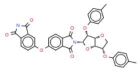	255	225± 17	250±34	[56]
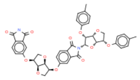	227	223± 27	228±22	[56]
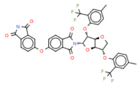	246	204± 27	245±35	[56]
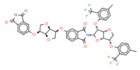	229	208± 26	234±32	[56]
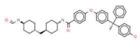	217	197± 20	204±38	[36]
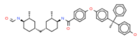	237	229± 23	199±45	[36]
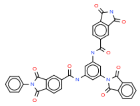	327	342± 21	327±23	[3]
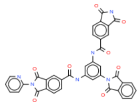	332	338± 22	334±28	[3]
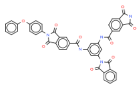	356	352± 21	343±27	[3]
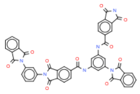	371	374± 25	351±25	[3]
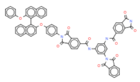	333	354± 52	337±28	[3]
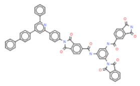	362	350± 48	350±26	[3]
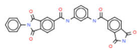	301	284± 14	314±35	[3]
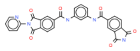	303	283± 14	311±39	[3]
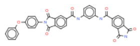	319	289± 17	310±32	[3]
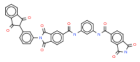	349	306± 29	313±39	[3]
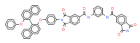	304	318± 32	313±32	[3]
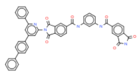	336	307± 33	316±32	[3]
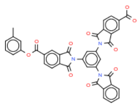	264	296± 17	314±27	[2]
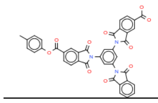	259	281± 17	318±28	[2]

Continued on next page

Table S4 – *Continued from previous page*

MONOMER	T_g^{exp}	$\widehat{T_g^{PLSR}}$	$\widehat{T_g^{RF}}$	Ref
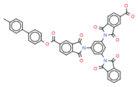	301	322± 19	310±24	[2]
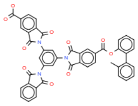	305	348± 17	324±31	[2]
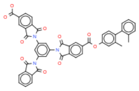	317	350± 16	319±32	[2]
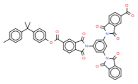	306	306± 20	307±28	[2]
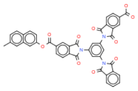	328	311± 18	320±26	[2]
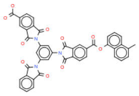	331	335± 18	325±27	[2]
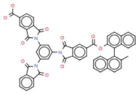	353	383± 21	344±22	[2]
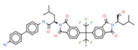	291	296± 15	255±30	[47]
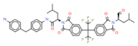	284	287± 17	256±29	[47]
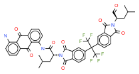	245	333± 23	261±34	[47]
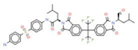	255	293± 23	258±38	[47]
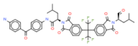	263	286± 19	268±34	[47]
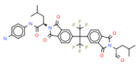	268	290± 21	256±32	[47]
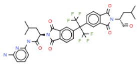	251	296± 21	256±36	[47]
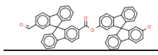	334	276± 45	263±88	[45]

Table S5: The table lists the experimental and predicted (± uncertainty) 10% thermal decomposition temperatures for different polymers. Predictions for only the RF model are reported.

MONOMER	T_D^{exp}	T_D^{pred}	Ref
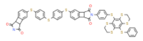	443	415 ± 68	[61]
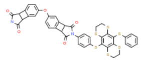	391	401 ± 60	[61]
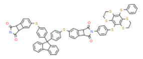	424	410 ± 69	[61]
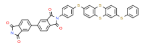	563	546 ± 32	[25]
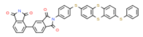	546	536 ± 30	[25]
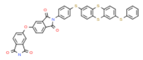	527	531 ± 21	[25]
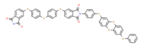	508	489 ± 36	[25]
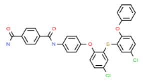	442	453 ± 25	[15]
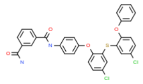	435	451 ± 26	[15]
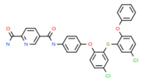	436	468 ± 47	[15]
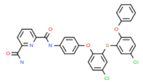	430	460 ± 34	[15]
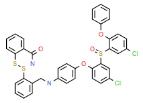	415	477 ± 45	[15]
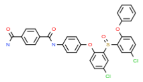	450	453 ± 15	[15]
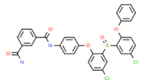	447	448 ± 17	[15]
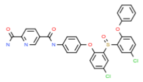	448	457 ± 29	[15]
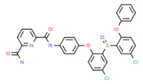	445	448 ± 34	[15]
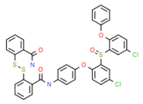	426	458 ± 44	[15]
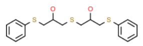	125	300 ± 75	[22]
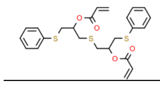	235	273 ± 66	[22]

Continued on next page

Table S5 – *Continued from previous page*

MONOMER	T_D^{exp}	T_D^{pred}	Ref
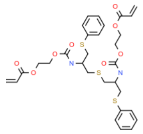	237	284 ± 68	[22]
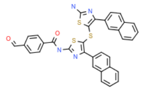	513	499 ± 36	[16]
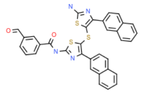	501	489 ± 40	[16]
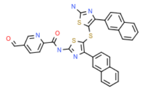	507	495 ± 36	[16]
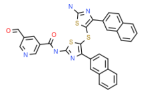	496	493 ± 40	[16]
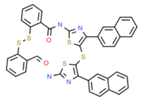	478	475 ± 47	[16]
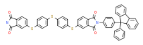	497	499 ± 19	[53]
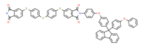	494	487 ± 49	[53]
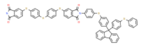	496	501 ± 39	[53]
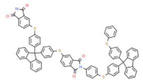	532	516 ± 34	[53]
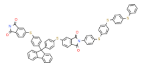	513	491 ± 58	[53]
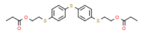	365	380 ± 58	[59]
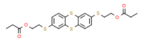	365	355 ± 65	[59]
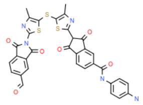	510	506 ± 20	[48]
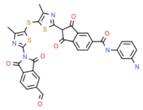	496	499 ± 25	[48]
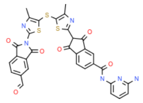	503	502 ± 30	[48]
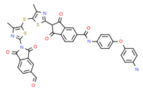	482	491 ± 36	[48]
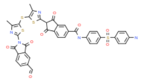	507	441 ± 65	[48]
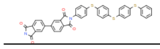	529	528 ± 19	[26]

Continued on next page

Table S5 – *Continued from previous page*

MONOMER	T_D^{exp}	T_D^{pred}	Ref
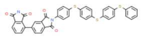	536	521 ± 27	[26]
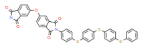	532	534 ± 23	[26]
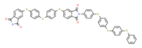	500	497 ± 35	[26]
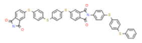	504	510 ± 32	[26]
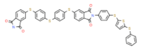	456.2	504 ± 31	10.1002/pola.23497
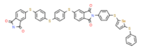	453.3	474 ± 61	10.1002/pola.23497
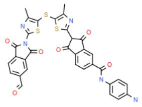	510	506 ± 20	[48]
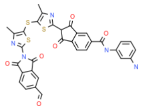	496	499 ± 25	[48]
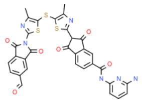	503	502 ± 30	[48]
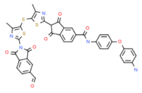	482	491 ± 36	[48]
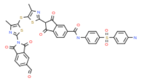	507	441 ± 65	[48]
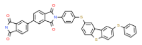	557.6	528 ± 34	[27]
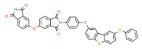	549.7	539 ± 35	[27]
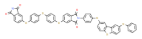	506.3	497 ± 30	[27]
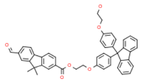	429	436 ± 40	[44]
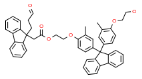	407	411 ± 30	[44]
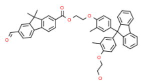	408	418 ± 31	[44]
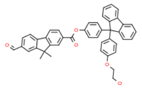	486	442 ± 41	[44]
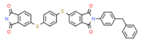	533	520 ± 31	[13]
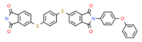	543	519 ± 35	[13]
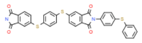	540	527 ± 39	[13]
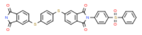	508	491 ± 63	[13]
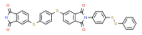	503	518 ± 54	[13]
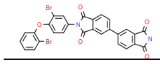	552	540 ± 29	[5]

Continued on next page

Table S5 – *Continued from previous page*

MONOMER	T_D^{exp}	T_D^{pred}	Ref
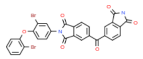	500	507 ± 22	[5]
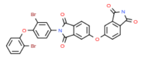	554	539 ± 33	[5]
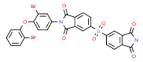	523	513 ± 38	[5]
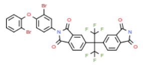	517	505 ± 51	[5]
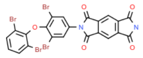	539	512 ± 56	[5]
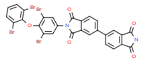	563	550 ± 38	[5]
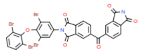	484	500 ± 36	[5]
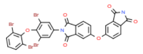	537	536 ± 35	[5]
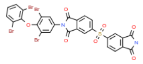	531	510 ± 46	[5]
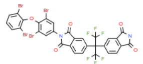	549	523 ± 37	[5]
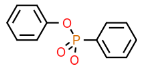	357	421 ± 65	[30]
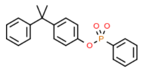	433	430 ± 45	[30]
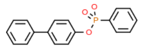	327	423 ± 63	[30]
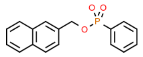	242	391 ± 92	[30]
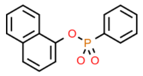	241	422 ± 63	[30]
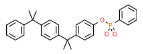	430	430 ± 51	[30]
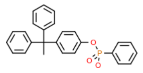	471	405 ± 88	[30]
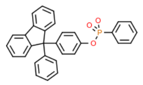	484	465 ± 39	[30]
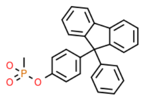	414	428 ± 51	[30]
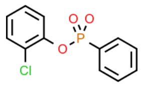	405	422 ± 62	[30]
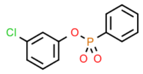	433	425 ± 60	[30]
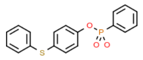	443	422 ± 62	[30]
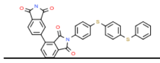	540	527 ± 34	[8]

Continued on next page

Table S5 – *Continued from previous page*

MONOMER	T_D^{exp}	T_D^{pred}	Ref
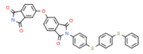	529	524 ± 21	[8]
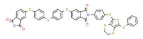	478	448 ± 58	[57]
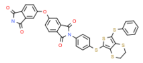	409	477 ± 51	[57]
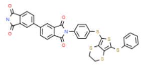	521	480 ± 50	[57]
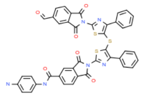	522	502 ± 21	[19]
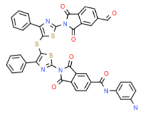	507	501 ± 24	[19]
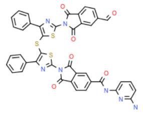	510	503 ± 24	[19]
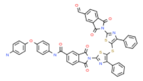	490	489 ± 52	[19]
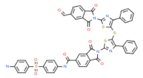	518	495 ± 28	[19]
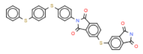	542	540 ± 30	[24]
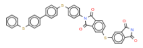	545	516 ± 33	[24]
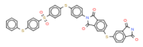	521	512 ± 38	[24]
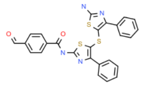	542	479 ± 46	[17]
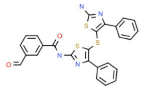	545	478 ± 44	[17]
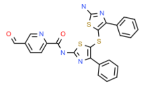	521	488 ± 53	[17]
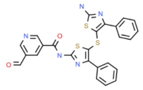	556	481 ± 49	[17]
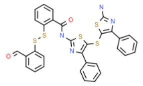	536	507 ± 36	[17]
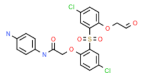	423	445 ± 34	[20]
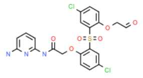	404	417 ± 80	[20]
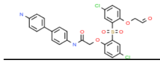	421	450 ± 36	[20]

Continued on next page

Table S5 – *Continued from previous page*

MONOMER	T_D^{exp}	T_D^{pred}	Ref
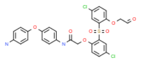	401	428 ± 76	[20]
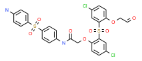	410	465 ± 44	[20]
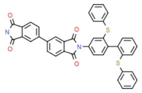	498	506 ± 22	[52]
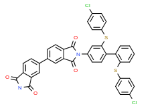	507	519 ± 34	[52]
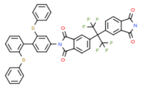	495	497 ± 44	[52]
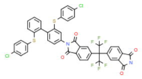	506	503 ± 34	[52]
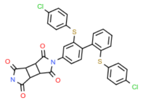	467	459 ± 49	[52]
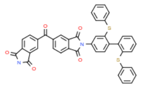	509	513 ± 18	[52]
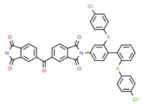	512	513 ± 32	[52]
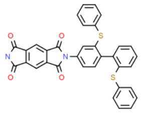	501	510 ± 40	[52]
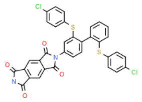	505	516 ± 32	[52]
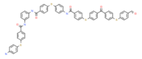	427	440 ± 37	[64]
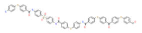	439	477 ± 48	[64]
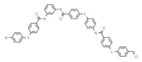	431	438 ± 35	[64]
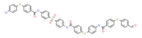	439	473 ± 44	[64]
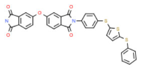	443	469 ± 45	[7]
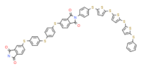	374	432 ± 74	[7]
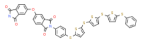	354	419 ± 66	[7]
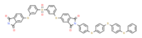	511	473 ± 63	[9]
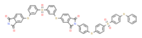	494	500 ± 34	[9]
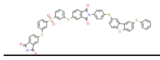	506	494 ± 38	[9]

Continued on next page

Table S5 – *Continued from previous page*

**MONOMER**	T_D^{exp}	T_D^{pred}	**Ref**
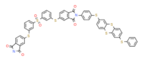	498	459 ± 64	[9]
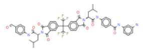	363.9	372 ± 31	[33]
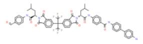	369	372 ± 30	[33]
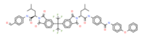	363.2	380 ± 30	[33]
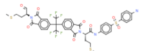	357	406 ± 86	[31]
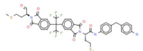	350	362 ± 55	[31]
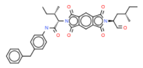	350	376 ± 49	[32]
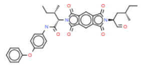	340	385 ± 63	[32]
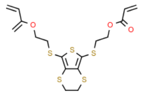	312.5	365 ± 85	[21]
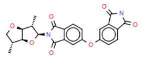	460	442 ± 52	[56]
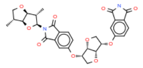	445	436 ± 42	[56]
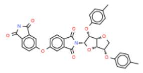	439	466 ± 44	[56]
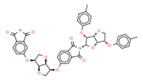	433	440 ± 27	[56]
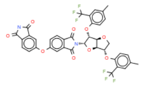	450	461 ± 35	[56]
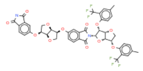	440	446 ± 22	[56]
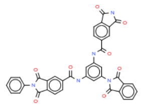	516	512 ± 21	[3]
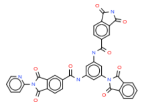	520	508 ± 23	[3]
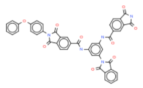	528	510 ± 25	[3]
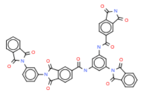	529	501 ± 30	[3]
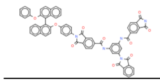	543	524 ± 32	[3]

Continued on next page

Table S5 – *Continued from previous page*

MONOMER	T_D^{exp}	T_D^{pred}	Ref
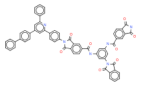	539	514 ± 30	[3]
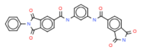	506	506 ± 34	[3]
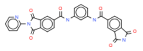	508	509 ± 24	[3]
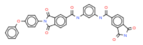	513	503 ± 36	[3]
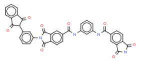	516	502 ± 27	[3]
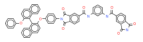	522	514 ± 36	[3]
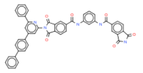	518	515 ± 25	[3]
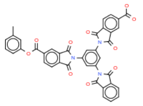	455	466 ± 25	[2]
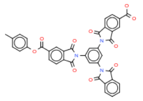	461	465 ± 25	[2]
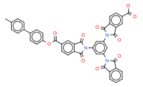	473	466 ± 29	[2]
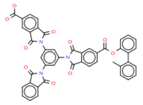	477	464 ± 26	[2]
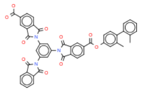	458	464 ± 24	[2]
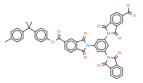	451	454 ± 29	[2]
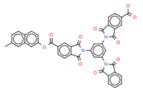	460	464 ± 27	[2]
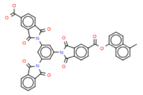	468	465 ± 24	[2]
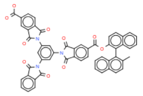	482	474 ± 29	[2]
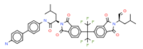	396	415 ± 41	[47]
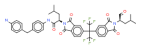	440	423 ± 30	[47]
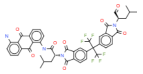	383	406 ± 38	[47]
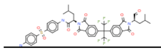	412	425 ± 45	[47]

Continued on next page

Table S5 – *Continued from previous page*

MONOMER	T_D^{exp}	T_D^{pred}	Ref
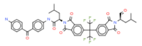	425	408 ± 38	[47]
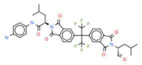	389	398 ± 41	[47]
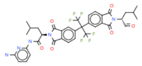	375	390 ± 49	[47]
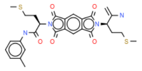	250	270 ± 50	[10]
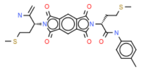	255	268 ± 46	[10]
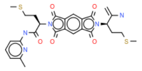	300	290 ± 56	[10]
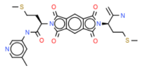	318	298 ± 57	[10]
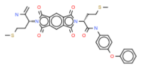	300	299 ± 53	[10]
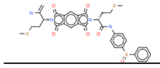	280	311 ± 68	[10]

Table S6: The table lists the experimental and predicted (± uncertainty) densities for different polymers. Predictions for only the RF model are reported. The experimental values are taken from multiple references[23, 4, 34]

MONOMER	ρ_{exp}	ρ_{pred}
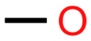	1.42	1.22 ± 0.18
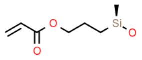	1.11	1.09 ± 0.17
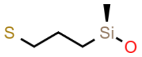	1.06	1.14 ± 0.27
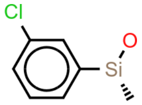	1.10	1.17 ± 0.20
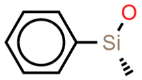	1.11	1.09 ± 0.09
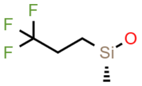	1.30	1.30 ± 0.25
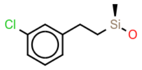	1.09	1.15 ± 0.20
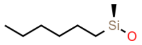	0.91	0.93 ± 0.08
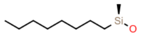	0.91	0.98 ± 0.16
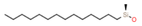	0.89	0.92 ± 0.10
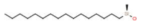	0.88	0.93 ± 0.13
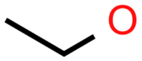	1.12	1.14 ± 0.17
	0.89	0.98 ± 0.13
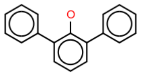	1.14	1.18 ± 0.12
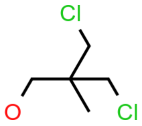	1.39	1.38 ± 0.16
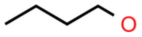	0.98	1.03 ± 0.17
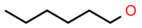	0.93	1.01 ± 0.14
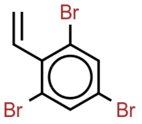	2.10	1.93 ± 0.33
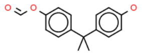	1.20	1.18 ± 0.10
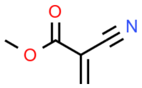	1.30	1.25 ± 0.09
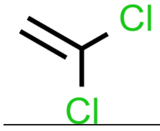	1.66	1.48 ± 0.23

Continued on next page

Table S6 – *Continued from previous page*

MONOMER	ρ_{exp}	ρ_{pred}
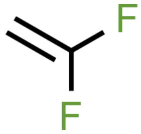	1.60	1.46 ± 0.20
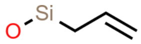	0.98	1.01 ± 0.09
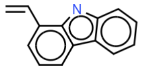	1.20	1.17 ± 0.11
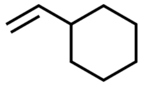	0.95	0.95 ± 0.14
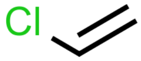	1.38	1.40 ± 0.14
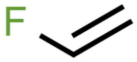	1.38	1.39 ± 0.15
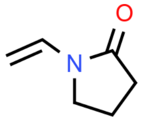	1.25	1.18 ± 0.09
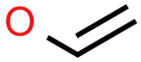	1.26	1.02 ± 0.15
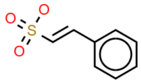	1.10	1.17 ± 0.13
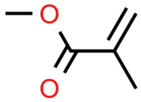	1.17	1.13 ± 0.05
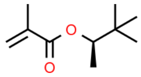	0.99	1.01 ± 0.04
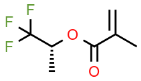	1.34	1.37 ± 0.20
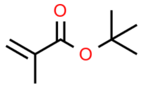	1.02	1.03 ± 0.11
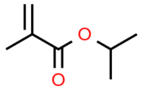	1.03	1.05 ± 0.09
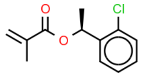	1.27	1.33 ± 0.17
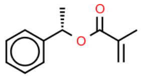	1.13	1.12 ± 0.08
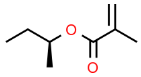	1.05	1.05 ± 0.06
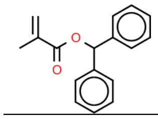	1.17	1.19 ± 0.08

Continued on next page

MONOMER	ρ_{exp}	ρ_{pred}
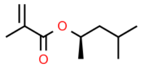	1.00	1.02 ± 0.05
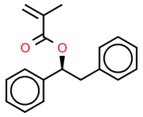	1.15	1.16 ± 0.07
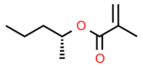	1.03	1.04 ± 0.06
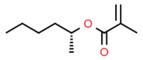	1.01	1.02 ± 0.05
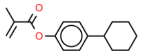	1.12	1.14 ± 0.07
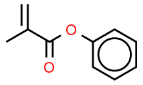	1.21	1.17 ± 0.09
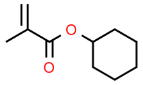	1.10	1.03 ± 0.07
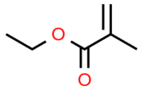	1.11	1.10 ± 0.06
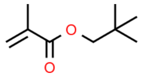	0.99	1.04 ± 0.07
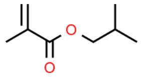	1.04	1.05 ± 0.05
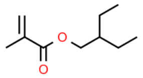	1.04	1.03 ± 0.06
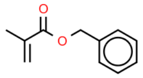	1.18	1.10 ± 0.09
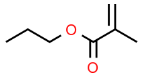	1.08	1.06 ± 0.09
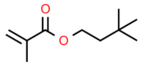	1.00	1.02 ± 0.05
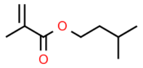	1.03	1.04 ± 0.05
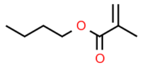	1.05	1.03 ± 0.04
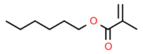	1.01	1.03 ± 0.04
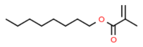	0.97	1.01 ± 0.06
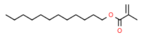	0.93	1.02 ± 0.05
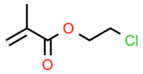	1.32	1.30 ± 0.18
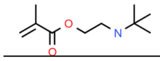	1.15	1.11 ± 0.06

Continued on next page

**MONOMER**	ρ_{exp}	ρ_{pred}
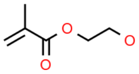	1.15	1.15 ± 0.14
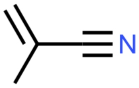	1.10	1.06 ± 0.13
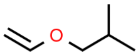	0.93	0.99 ± 0.10
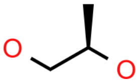	1.00	1.10 ± 0.17
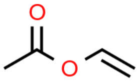	1.19	1.12 ± 0.09
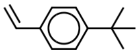	0.95	1.05 ± 0.10
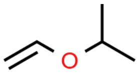	0.92	0.98 ± 0.12
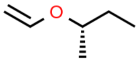	0.92	0.96 ± 0.10
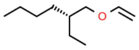	0.90	0.96 ± 0.10
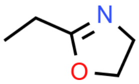	1.14	1.11 ± 0.10
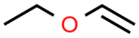	0.95	0.99 ± 0.12
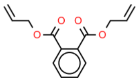	1.27	1.26 ± 0.09
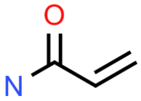	1.30	1.25 ± 0.14
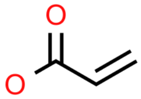	1.41	1.20 ± 0.21
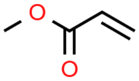	1.22	1.18 ± 0.12
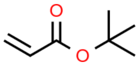	1.00	1.07 ± 0.11
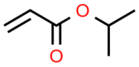	1.08	1.09 ± 0.06
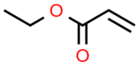	1.12	1.10 ± 0.08
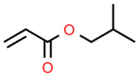	1.06	1.09 ± 0.05
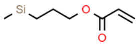	1.11	1.04 ± 0.12
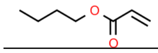	1.09	1.09 ± 0.06

Continued on next page

Table S6 – *Continued from previous page*

MONOMER	ρ_{exp}	ρ_{pred}
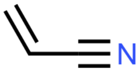	1.18	1.17 ± 0.10
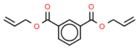	1.26	1.25 ± 0.09
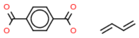	1.31	1.26 ± 0.21
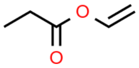	1.02	1.06 ± 0.08
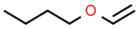	0.93	0.96 ± 0.11
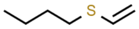	0.98	1.09 ± 0.14
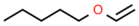	1.04	0.98 ± 0.14
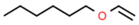	0.92	0.94 ± 0.13
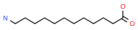	0.99	1.05 ± 0.11
	0.91	0.95 ± 0.17
	0.88	0.98 ± 0.12
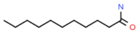	1.04	1.08 ± 0.11
	0.89	0.95 ± 0.14
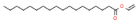	1.00	1.12 ± 0.10
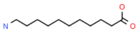	1.01	1.04 ± 0.12
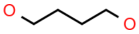	0.97	1.03 ± 0.14
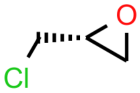	1.36	1.33 ± 0.15
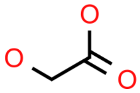	1.60	1.22 ± 0.15
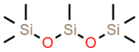	0.99	1.12 ± 0.21
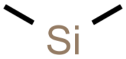	0.91	0.92 ± 0.07
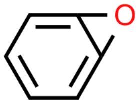	1.06	1.16 ± 0.12
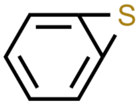	1.36	1.22 ± 0.12
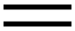	0.85	0.91 ± 0.10
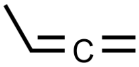	0.91	0.94 ± 0.10
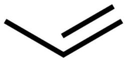	0.85	0.88 ± 0.10
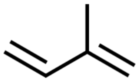	0.91	0.95 ± 0.11
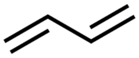	0.89	0.98 ± 0.12
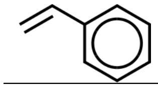	1.05	1.02 ± 0.10

Continued on next page

Table S6 – *Continued from previous page*

MONOMER	ρ_{exp}	ρ_{pred}
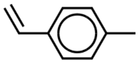	1.04	1.03 ± 0.10
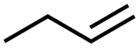	0.86	0.90 ± 0.08
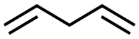	0.89	0.92 ± 0.10
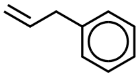	1.05	1.04 ± 0.10
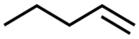	0.85	0.90 ± 0.10
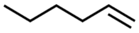	0.86	0.90 ± 0.07
	0.86	0.93 ± 0.10
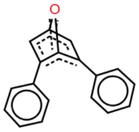	1.14	1.25 ± 0.22
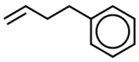	1.04	1.06 ± 0.11
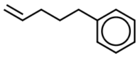	1.05	1.05 ± 0.11
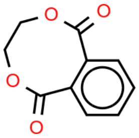	1.34	1.26 ± 0.22
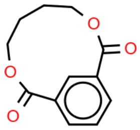	1.27	1.23 ± 0.13
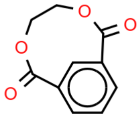	1.34	1.25 ± 0.22
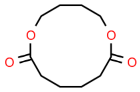	1.02	1.03 ± 0.07
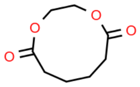	1.18	1.13 ± 0.09
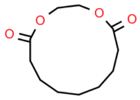	1.18	1.07 ± 0.10
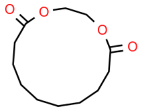	1.08	1.07 ± 0.10
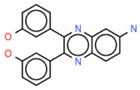	1.07	1.21 ± 0.09
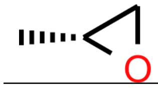	1.00	1.23 ± 0.12

Continued on next page

MONOMER	ρ_{exp}	ρ_{pred}
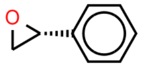	1.15	1.20 ± 0.10
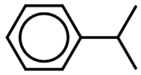	1.06	1.04 ± 0.11
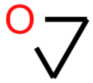	1.21	1.21 ± 0.11
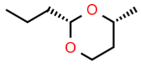	1.08	1.13 ± 0.16
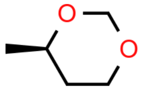	1.23	1.11 ± 0.18
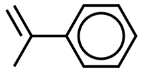	1.07	1.03 ± 0.10
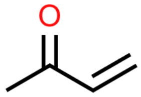	1.12	1.13 ± 0.11
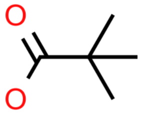	1.10	1.04 ± 0.12
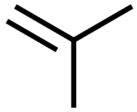	0.84	0.89 ± 0.07
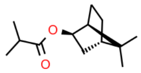	1.06	1.03 ± 0.12
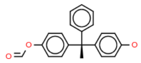	1.20	1.20 ± 0.09
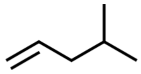	0.84	0.91 ± 0.07
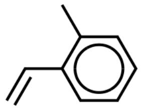	1.03	1.02 ± 0.10
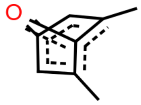	1.06	1.10 ± 0.18
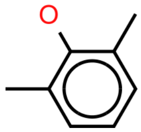	1.07	1.08 ± 0.15
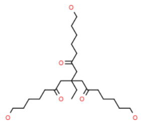	1.07	1.14 ± 0.12
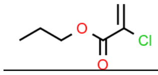	1.30	1.26 ± 0.09

Continued on next page

Table S6 – *Continued from previous page*

MONOMER	ρ_{exp}	ρ_{pred}
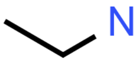	1.07	1.11 ± 0.15
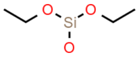	1.06	1.07 ± 0.08
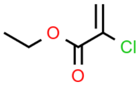	1.39	1.35 ± 0.12
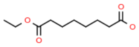	1.15	1.07 ± 0.12
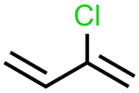	1.24	1.43 ± 0.16
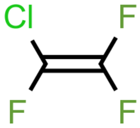	1.92	1.37 ± 0.18
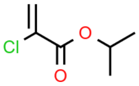	1.27	1.28 ± 0.09
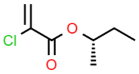	1.24	1.28 ± 0.09
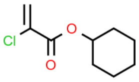	1.25	1.29 ± 0.10
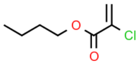	1.24	1.29 ± 0.13
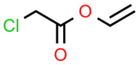	1.45	1.40 ± 0.18
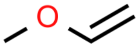	1.05	1.03 ± 0.16
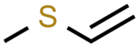	1.18	1.17 ± 0.14
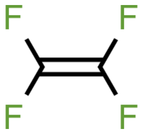	2.00	1.38 ± 0.20
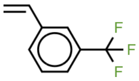	1.32	1.37 ± 0.21
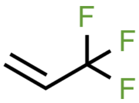	1.58	1.44 ± 0.21
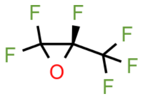	1.91	1.38 ± 0.20
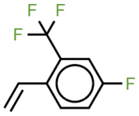	1.43	1.38 ± 0.20
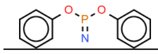	1.20	1.27 ± 0.22

Continued on next page

MONOMER	ρ_{exp}	ρ_{pred}
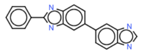	1.30	1.27 ± 0.18
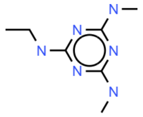	1.48	1.14 ± 0.12
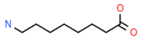	1.04	1.09 ± 0.13
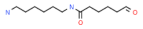	1.14	1.14 ± 0.10
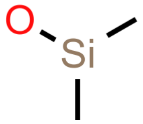	0.98	0.96 ± 0.09
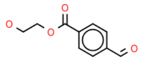	1.46	1.17 ± 0.11
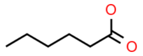	1.14	1.10 ± 0.10
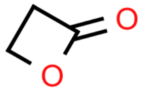	1.36	1.37 ± 0.28
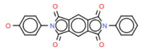	1.42	1.21 ± 0.10
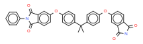	1.27	1.23 ± 0.09
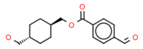	1.20	1.18 ± 0.12
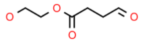	1.18	1.13 ± 0.07
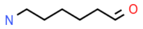	1.12	1.14 ± 0.11
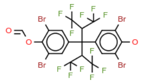	1.99	1.96 ± 0.26
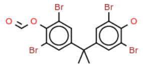	1.95	1.88 ± 0.34
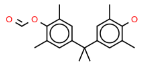	1.08	1.12 ± 0.10
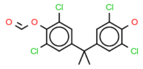	1.42	1.35 ± 0.12
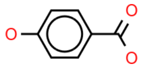	1.44	1.34 ± 0.13
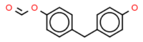	1.24	1.21 ± 0.09
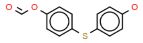	1.36	1.32 ± 0.09
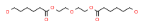	1.07	1.20 ± 0.13
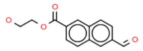	1.33	1.21 ± 0.10
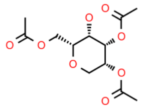	1.31	1.15 ± 0.11
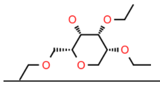	1.14	1.20 ± 0.14

Continued on next page

Table S6 – *Continued from previous page*

MONOMER	ρ_{exp}	ρ_{pred}
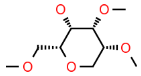	1.39	1.27 ± 0.15
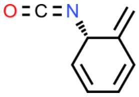	1.24	1.20 ± 0.09
	1.19	1.17 ± 0.11
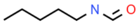	1.23	1.16 ± 0.12
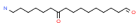	1.16	1.16 ± 0.10
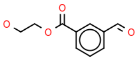	1.36	1.22 ± 0.10
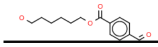	1.13	1.18 ± 0.11

Table S7: Summary of the classification model performances for the polymer solubilities in different solvents (NMP, THF, $CHCl_3$, DMSO and DMAC). The solubility classes include: S - soluble, PS - partially soluble/swelling/soluble on heating and I - insoluble. Here, mtry is the number of predictors sampled for splitting at each node in the RF model, while accuracy and kappa coefficient are used as the performance metrics.

Solvent	I/PS/S	ACCURACY	KAPPA	*mtry*	I/PS/S	ACCURACY	KAPPA
$CHCl_3$	27/17/24	0.71	0.56	8	26/17/24	0.75	0.61
DMAC	4/21/28	0.74	0.52	15	4/20/28	0.73	0.48
DMSO	10/28/40	0.73	0.53	8	9/28/39	0.75	0.57
NMP	5/21/47	0.83	0.62	42	5/21/46	0.72	0.36
THF	8/30/23	0.72	0.49	51	7/29/23	0.78	0.62

Table S8: The table lists the experimental and predicted solubility classes (S - soluble, PS - partially soluble/swelling/soluble on heating, I - insoluble) for different polymers in $CHCl_3$. Predictions for the RF model are reported.

MONOMER	*Experimental*	*Predicted*	**Ref**
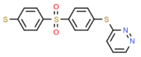	PS	I	[63]
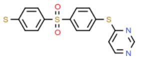	I	I	[63]
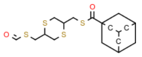	S	S	[60]
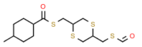	S	S	[60]
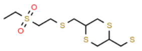	S	S	[60]
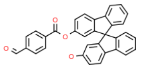	S	S	[45]
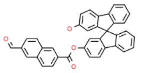	S	S	[45]
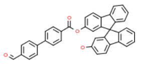	S	S	[45]
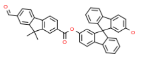	S	S	[45]
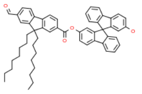	S	S	[45]
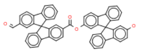	S	S	[45]
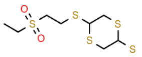	S	I	[43]
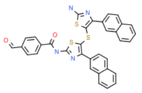	S	S	[16]
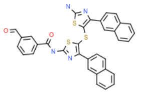	PS	PS	[16]
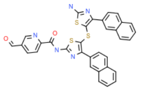	PS	PS	[16]
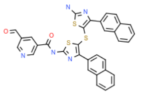	PS	PS	[16]
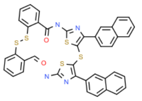	PS	PS	[16]
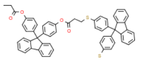	S	S	[38]
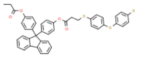	S	S	[38]
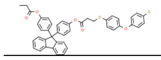	S	S	[38]

Continued on next page

Table S8 – *Continued from previous page*

MONOMER	*Experimental*	*Predicted*	Ref
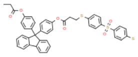	S	I	[38]
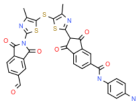	I	I	[48]
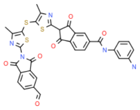	I	I	[48]
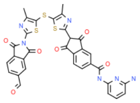	I	I	[48]
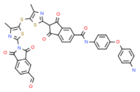	PS	PS	[48]
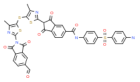	I	I	[48]
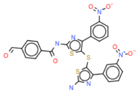	I	PS	[18]
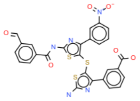	PS	PS	[18]
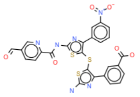	S	S	[18]
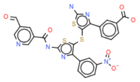	PS	PS	[18]
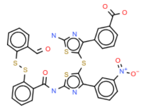	PS	PS	[18]
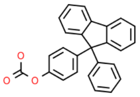	S	S	[46]
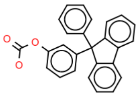	S	S	[46]
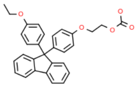	S	S	[46]
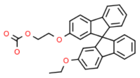	S	S	[46]
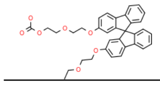	S	S	[46]

Continued on next page

**MONOMER**	*Experimental*	*Predicted*	**Ref**
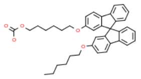	S	S	[46]
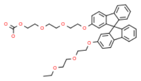	S	S	[46]
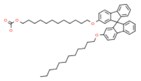	S	S	[46]
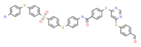	I	I	[65]
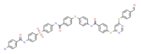	I	I	[65]
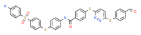	I	I	[65]
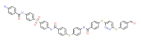	I	I	[65]
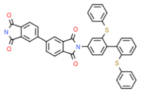	PS	I	[52]
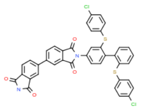	PS	PS	[52]
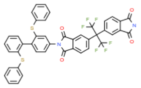	I	I	[52]
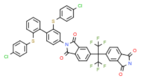	I	I	[52]
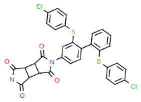	I	S	[52]
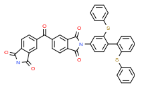	I	I	[52]
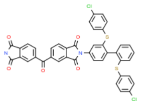	I	I	[52]
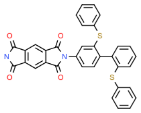	I	PS	[52]
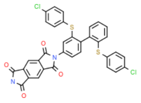	I	PS	[52]
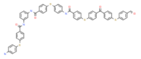	I	I	[64]
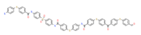	I	I	[64]
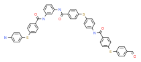	I	I	[64]
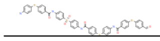	I	I	[64]

Continued on next page

Table S8 – *Continued from previous page*

MONOMER	*Experimental*	*Predicted*	**Ref**
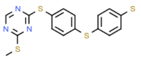	S	S	[40]
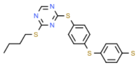	S	S	[40]
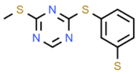	S	S	[40]
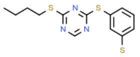	S	S	[40]
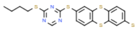	S	S	[40]
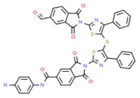	I	I	[19]
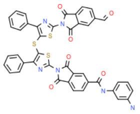	S	S	[19]
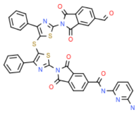	PS	PS	[19]
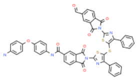	PS	I	[19]
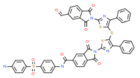	I	I	[19]
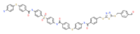	I	I	[35]
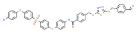	I	I	[35]
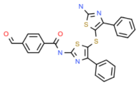	I	I	[17]
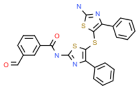	PS	I	[17]
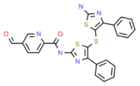	I	I	[17]
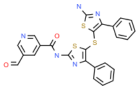	PS	PS	[17]
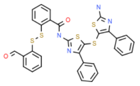	PS	PS	[17]
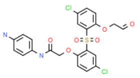	I	I	[20]
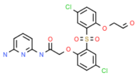	PS	PS	[20]
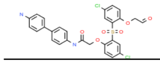	I	I	[20]

Continued on next page

Table S8 – *Continued from previous page*

MONOMER	*Experimental*	*Predicted*	Ref
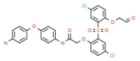	PS	I	[20]
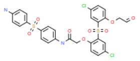	I	I	[20]
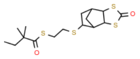	S	S	[42]
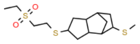	I	I	[50]
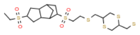	I	I	[50]
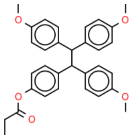	S	S	[54]
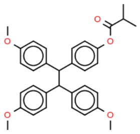	S	S	[54]
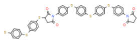	S	S	[39]
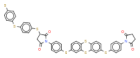	S	S	[39]
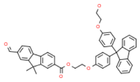	S	S	[44]
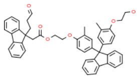	S	S	[44]
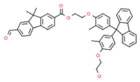	S	S	[44]
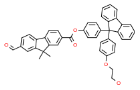	S	S	[44]
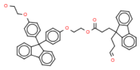	S	S	[44]
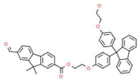	S	S	[44]
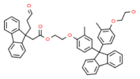	S	S	[44]
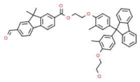	S	S	[44]
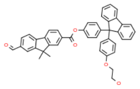	S	S	[44]
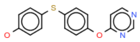	S	S	[37]
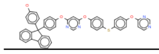	S	S	[37]

Continued on next page

Table S8 – *Continued from previous page*

MONOMER	*Experimental*	*Predicted*	**Ref**
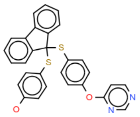	S	S	[37]
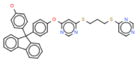	S	S	[37]
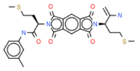	I	I	[10]
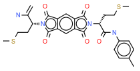	I	I	[10]
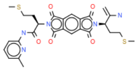	I	I	[10]
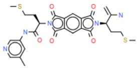	I	I	[10]
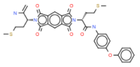	I	I	[10]
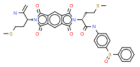	I	I	[10]
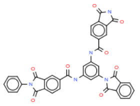	PS	PS	[3]
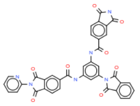	PS	PS	[3]
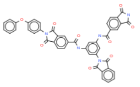	I	I	[3]
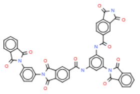	PS	PS	[3]
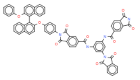	PS	PS	[3]
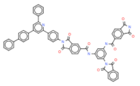	PS	PS	[3]
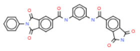	PS	I	[3]
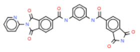	I	I	[3]
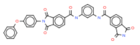	I	I	[3]
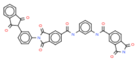	PS	PS	[3]
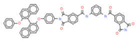	PS	I	[3]
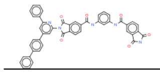	PS	PS	[3]

Continued on next page

Table S8 – *Continued from previous page*

MONOMER	*Experimental*	*Predicted*	**Ref**
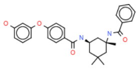	I	S	[66]
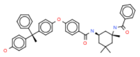	I	S	[66]
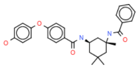	I	I	[66]
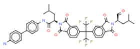	I	I	[47]
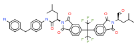	I	I	[47]
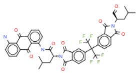	I	I	[47]
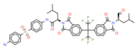	I	I	[47]
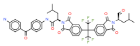	I	I	[47]
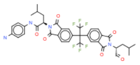	I	I	[47]
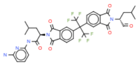	I	I	[47]
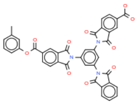	PS	PS	[2]
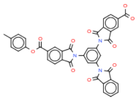	I	PS	[2]
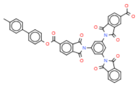	I	PS	[2]
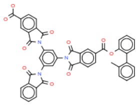	PS	PS	[2]
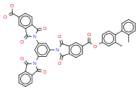	PS	PS	[2]
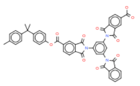	PS	PS	[2]
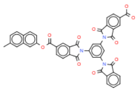	PS	PS	[2]
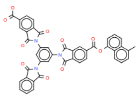	PS	PS	[2]
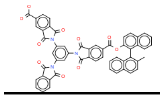	PS	PS	[2]

Table S9: The table lists the experimental and predicted solubility classes (S - soluble, PS - partially soluble/swelling/soluble on heating, I - insoluble) for different polymers in N-methylpyrrolidone (NMP). Predictions for the RF model are reported.

MONOMER	*Experimental*	*Predicted*	Ref
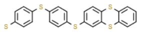	I	S	[51]
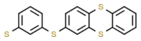	I	S	[51]
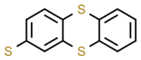	I	S	[51]
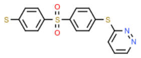	S	S	[63]
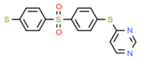	S	S	[63]
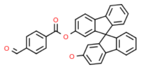	PS	PS	[45]
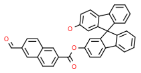	PS	PS	[45]
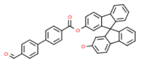	PS	PS	[45]
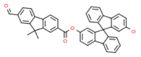	S	S	[45]
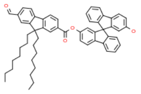	S	S	[45]
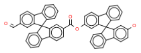	PS	PS	[45]
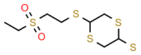	S	S	[43]
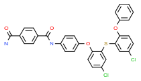	PS	S	[15]
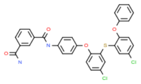	S	S	[15]
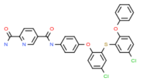	S	S	[15]
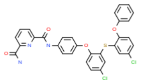	S	S	[15]
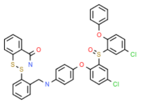	S	S	[15]
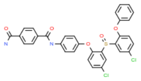	PS	S	[15]
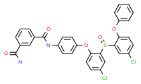	S	S	[15]
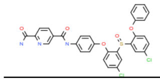	S	S	[15]

Continued on next page

Table S9 – *Continued from previous page*

MONOMER	*Experimental*	*Predicted*	Ref
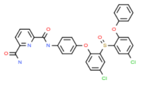	S	S	[15]
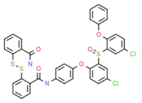	S	S	[15]
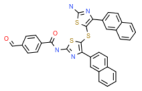	S	S	[16]
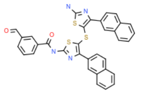	S	S	[16]
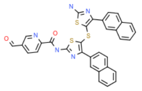	S	S	[16]
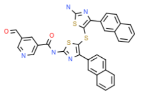	S	S	[16]
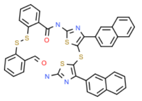	S	S	[16]
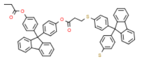	S	S	[38]
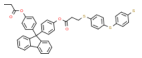	S	S	[38]
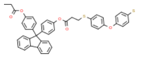	S	S	[38]
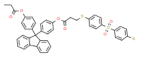	S	S	[38]
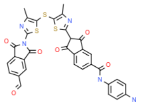	PS	S	[48]
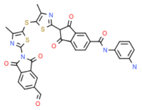	S	S	[48]
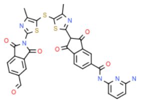	S	S	[48]
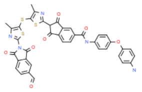	S	S	[48]
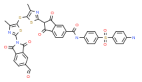	S	S	[48]
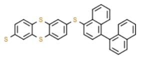	S	S	[41]
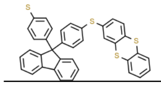	S	S	[41]

Continued on next page

Table S9 – *Continued from previous page*

MONOMER	*Experimental*	*Predicted*	**Ref**
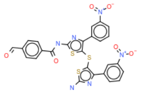	S	S	[18]
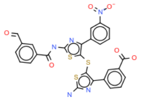	S	S	[18]
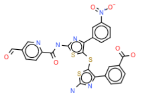	S	S	[18]
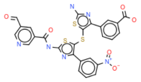	S	S	[18]
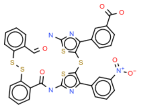	S	S	[18]
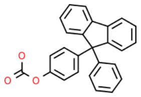	S	S	[46]
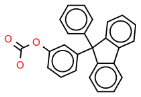	S	S	[46]
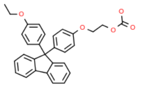	PS	S	[46]
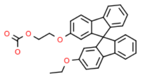	S	S	[46]
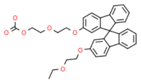	PS	S	[46]
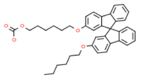	PS	PS	[46]
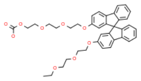	PS	PS	[46]
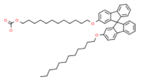	PS	PS	[46]
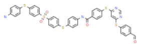	S	S	[65]
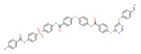	S	S	[65]
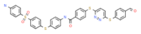	S	S	[65]
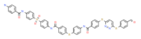	S	S	[65]
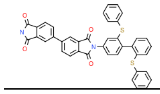	PS	PS	[52]

Continued on next page

Table S9 – *Continued from previous page*

MONOMER	*Experimental*	*Predicted*	Ref
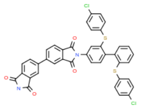	PS	I	[52]
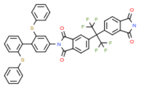	I	I	[52]
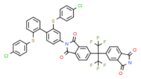	I	I	[52]
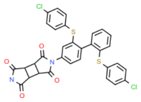	I	I	[52]
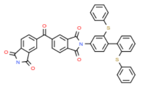	I	I	[52]
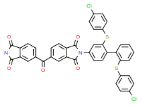	I	I	[52]
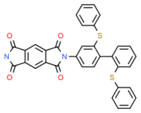	I	I	[52]
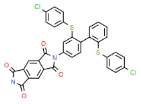	I	I	[52]
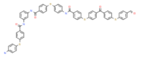	PS	S	[64]
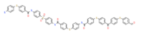	PS	PS	[64]
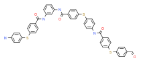	PS	S	[64]
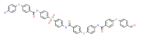	PS	PS	[64]
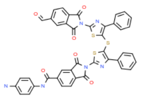	S	S	[19]
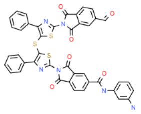	S	S	[19]
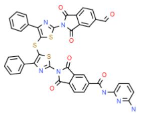	S	S	[19]
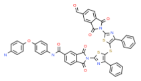	S	S	[19]
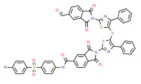	S	S	[19]
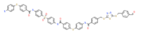	S	PS	[35]
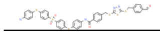	S	S	[35]

Continued on next page

Table S9 – *Continued from previous page*

**MONOMER**	*Experimental*	*Predicted*	**Ref**
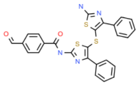	S	S	[17]
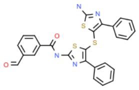	S	S	[17]
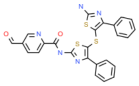	S	S	[17]
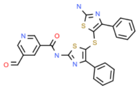	S	S	[17]
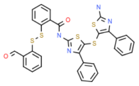	S	S	[17]
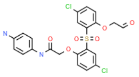	S	S	[20]
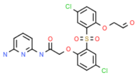	S	S	[20]
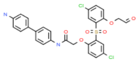	S	S	[20]
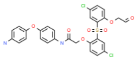	S	S	[20]
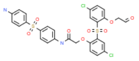	S	S	[20]
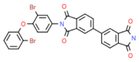	PS	S	[5]
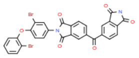	S	S	[5]
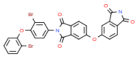	S	S	[5]
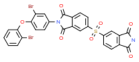	S	S	[5]
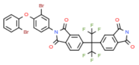	S	S	[5]
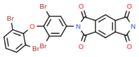	S	S	[5]
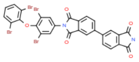	S	S	[5]
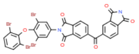	S	S	[5]
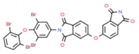	S	S	[5]
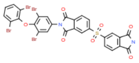	S	S	[5]
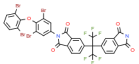	S	S	[5]
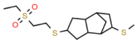	S	S	[50]
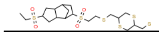	S	S	[50]

Continued on next page

Table S9 – *Continued from previous page*

**MONOMER**	*Experimental*	*Predicted*	**Ref**
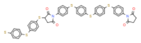	S	S	[39]
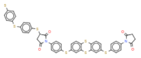	S	S	[39]
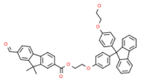	S	S	[44]
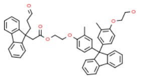	S	S	[44]
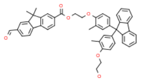	S	S	[44]
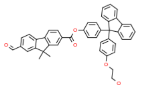	S	S	[44]
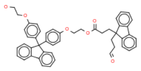	S	S	[44]
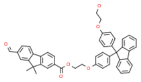	S	S	[44]
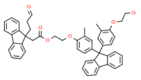	S	S	[44]
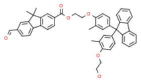	S	S	[44]
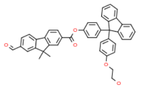	S	S	[44]
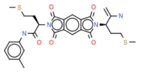	PS	PS	[10]
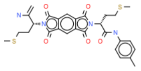	PS	PS	[10]
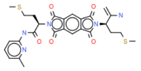	PS	PS	[10]
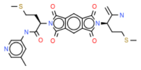	PS	PS	[10]
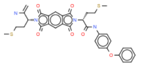	PS	PS	[10]
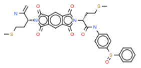	PS	S	[10]
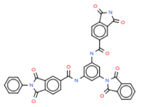	S	S	[3]
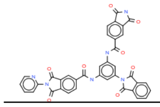	S	S	[3]

Continued on next page

Table S9 – *Continued from previous page*

MONOMER	*Experimental*	*Predicted*	**Ref**
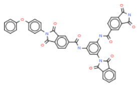	PS	S	[3]
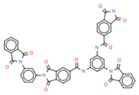	S	S	[3]
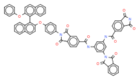	S	S	[3]
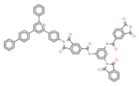	S	S	[3]
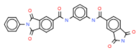	PS	S	[3]
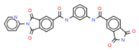	S	S	[3]
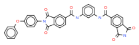	PS	PS	[3]
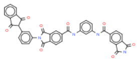	S	S	[3]
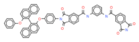	PS	S	[3]
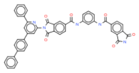	S	S	[3]
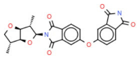	S	S	[56]
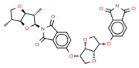	PS	PS	[56]
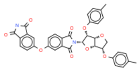	S	PS	[56]
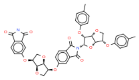	S	S	[56]
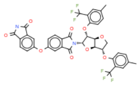	S	S	[56]
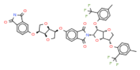	S	S	[56]
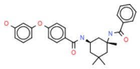	PS	PS	[66]
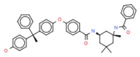	PS	S	[66]
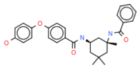	PS	PS	[66]
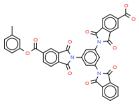	PS	PS	[2]
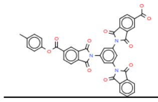	PS	PS	[2]

Continued on next page

Table S9 – *Continued from previous page*

**MONOMER**	*Experimental*	*Predicted*	**Ref**
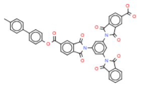	PS	PS	[2]
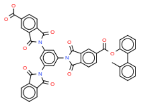	PS	PS	[2]
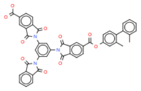	PS	PS	[2]
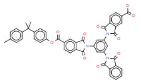	PS	PS	[2]
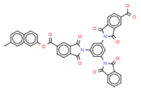	PS	PS	[2]
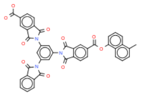	PS	PS	[2]
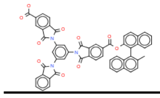	PS	PS	[2]

Table S10: The table lists the experimental and predicted solubility classes (S - soluble, PS - partially soluble/swelling/soluble on heating, I - insoluble) for different polymers in THF. Predictions for the RF model are reported.

MONOMER	*Experimental*	*Predicted*	Ref
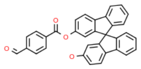	S	S	[45]
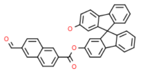	S	S	[45]
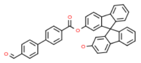	S	S	[45]
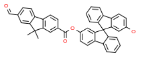	S	S	[45]
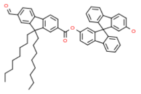	S	S	[45]
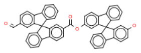	S	S	[45]
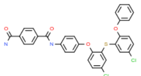	I	PS	[15]
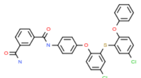	PS	PS	[15]
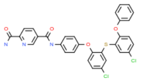	PS	PS	[15]
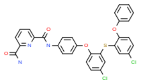	PS	PS	[15]
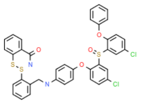	S	S	[15]
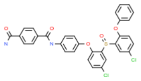	PS	PS	[15]
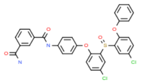	PS	PS	[15]
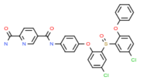	PS	PS	[15]
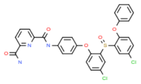	PS	PS	[15]
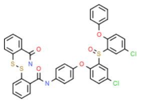	S	S	[15]
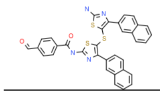	PS	PS	[16]

Continued on next page

Table S10 – *Continued from previous page*

MONOMER	*Experimental*	*Predicted*	**Ref**
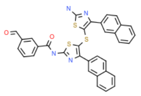	PS	PS	[16]
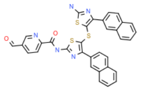	PS	PS	[16]
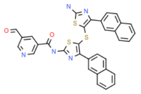	S	S	[16]
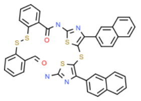	S	S	[16]
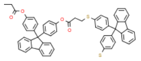	S	S	[38]
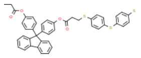	S	S	[38]
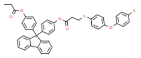	S	S	[38]
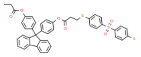	S	S	[38]
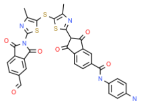	PS	PS	[48]
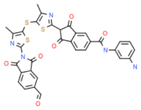	PS	PS	[48]
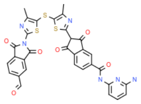	PS	PS	[48]
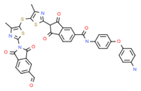	PS	PS	[48]
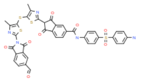	PS	PS	[48]
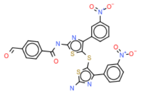	PS	PS	[18]
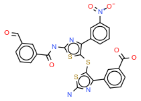	PS	PS	[18]
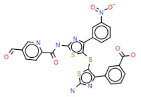	PS	PS	[18]
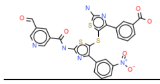	S	PS	[18]

Continued on next page

MONOMER	*Experimental*	*Predicted*	Ref
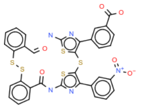	S	S	[18]
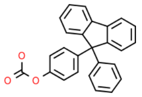	S	S	[46]
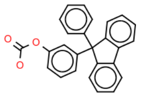	S	S	[46]
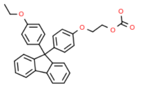	S	S	[46]
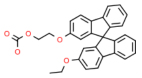	S	S	[46]
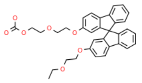	PS	S	[46]
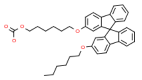	S	S	[46]
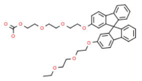	S	S	[46]
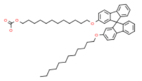	S	S	[46]
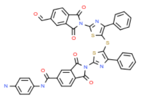	PS	PS	[19]
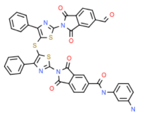	PS	PS	[19]
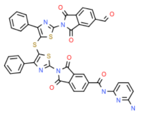	PS	PS	[19]
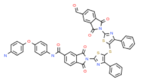	S	S	[19]
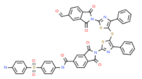	PS	PS	[19]
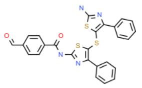	PS	PS	[17]
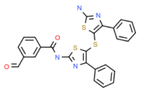	PS	PS	[17]
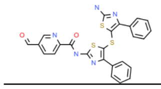	PS	S	[17]

Continued on next page

Table S10 – *Continued from previous page*

MONOMER	*Experimental*	*Predicted*	Ref
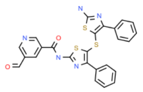	PS	PS	[17]
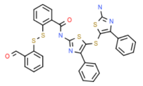	S	S	[17]
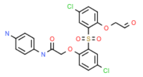	PS	PS	[20]
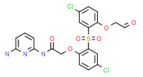	PS	PS	[20]
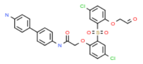	PS	PS	[20]
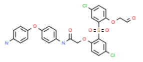	S	S	[20]
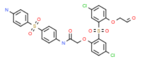	PS	PS	[20]
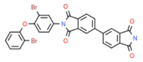	I	PS	[5]
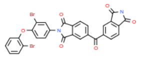	PS	PS	[5]
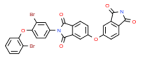	PS	PS	[5]
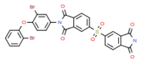	PS	PS	[5]
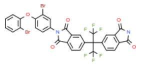	S	PS	[5]
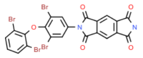	S	S	[5]
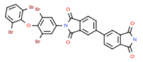	PS	S	[5]
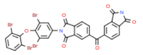	S	S	[5]
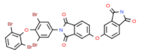	S	S	[5]
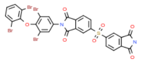	S	S	[5]
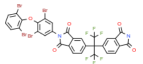	S	S	[5]
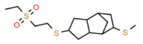	I	I	[50]
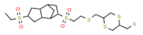	I	I	[50]
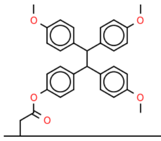	S	S	[54]

Continued on next page

Table S10 – *Continued from previous page*

**MONOMER**	*Experimental*	*Predicted*	**Ref**
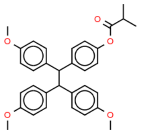	S	S	[54]
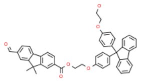	S	S	[44]
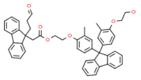	S	S	[44]
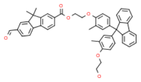	S	S	[44]
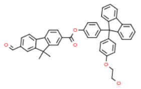	S	S	[44]
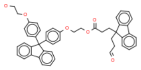	S	S	[44]
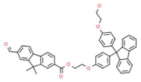	S	S	[44]
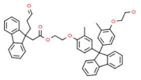	S	S	[44]
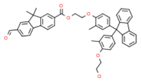	S	S	[44]
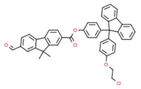	S	S	[44]
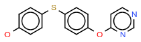	I	I	[37]
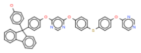	S	S	[37]
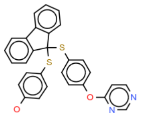	S	S	[37]
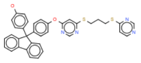	S	S	[37]
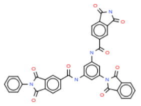	PS	PS	[3]
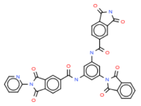	PS	PS	[3]
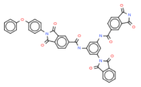	I	I	[3]
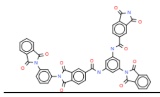	PS	PS	[3]

Continued on next page

Table S10 – *Continued from previous page*

MONOMER	*Experimental*	*Predicted*	**Ref**
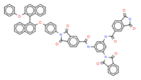	PS	PS	[3]
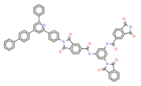	PS	PS	[3]
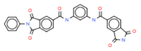	PS	I	[3]
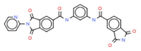	PS	PS	[3]
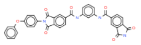	I	PS	[3]
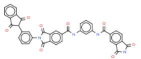	PS	PS	[3]
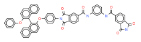	PS	PS	[3]
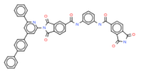	PS	PS	[3]
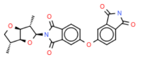	PS	PS	[56]
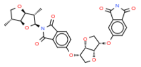	I	PS	[56]
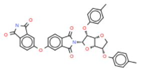	I	I	[56]
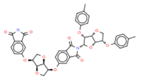	I	I	[56]
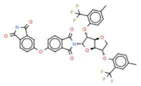	PS	PS	[56]
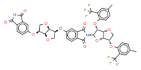	PS	PS	[56]
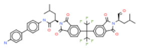	PS	PS	[47]
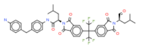	PS	PS	[47]
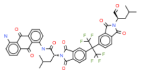	PS	PS	[47]
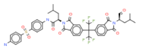	PS	PS	[47]
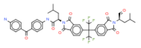	PS	PS	[47]
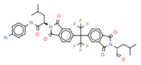	PS	PS	[47]
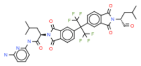	PS	PS	[47]
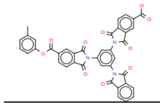	PS	I	[2]

Continued on next page

Table S10 – *Continued from previous page*

MONOMER	*Experimental*	*Predicted*	**Ref**
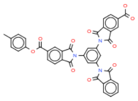	I	PS	[2]
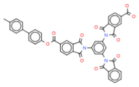	I	I	[2]
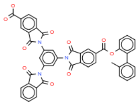	I	I	[2]
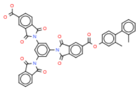	PS	I	[2]
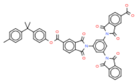	PS	PS	[2]
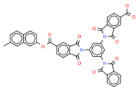	I	I	[2]
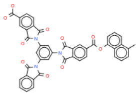	I	I	[2]
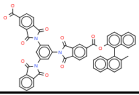	PS	PS	[2]

Table S11: The table lists the experimental and predicted solubility classes (S - soluble, PS - partially soluble/swelling/soluble on heating, I - insoluble) for different polymers in N,N-Dimethylacetamide (DMAc). Predictions for the RF model are reported.

**MONOMER**	*Experimental*	*Predicted*	**Ref**
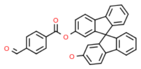	PS	PS	[45]
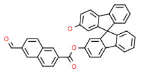	PS	PS	[45]
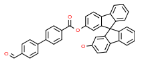	PS	PS	[45]
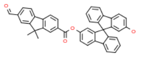	PS	PS	[45]
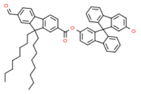	S	S	[45]
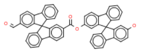	PS	S	[45]
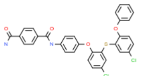	S	S	[15]
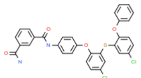	S	S	[15]
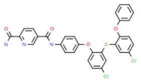	S	S	[15]
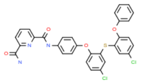	S	S	[15]
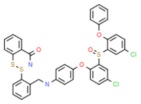	S	S	[15]
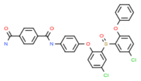	S	S	[15]
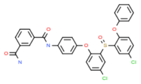	S	S	[15]
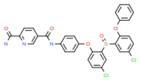	S	S	[15]
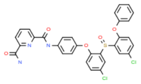	S	S	[15]
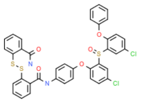	S	S	[15]
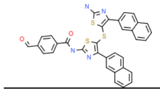	S	S	[16]

Continued on next page

Table S11 – *Continued from previous page*

MONOMER	*Experimental*	*Predicted*	Ref
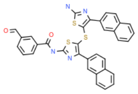	S	S	[16]
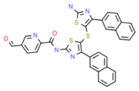	S	S	[16]
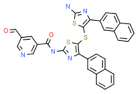	S	S	[16]
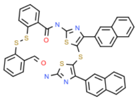	S	S	[16]
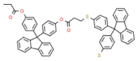	S	S	[38]
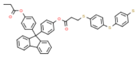	S	S	[38]
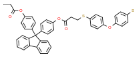	S	S	[38]
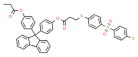	S	S	[38]
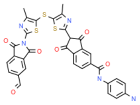	PS	PS	[48]
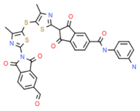	PS	S	[48]
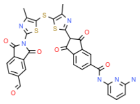	PS	S	[48]
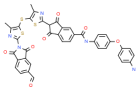	S	S	[48]
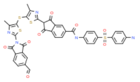	PS	PS	[48]
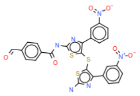	S	S	[18]
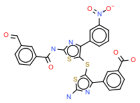	S	S	[18]
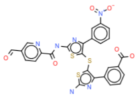	S	S	[18]
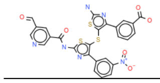	S	S	[18]

Continued on next page

Table S11 – *Continued from previous page*

MONOMER	*Experimental*	*Predicted*	Ref
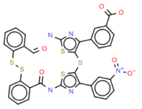	S	S	[18]
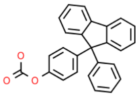	S	S	[46]
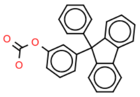	S	S	[46]
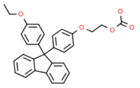	S	S	[46]
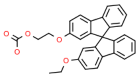	S	S	[46]
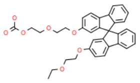	S	PS	[46]
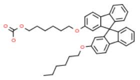	PS	PS	[46]
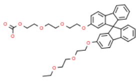	PS	S	[46]
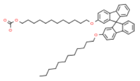	PS	PS	[46]
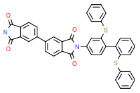	PS	PS	[52]
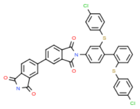	PS	I	[52]
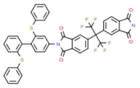	I	PS	[52]
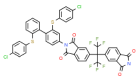	I	I	[52]
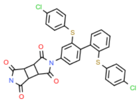	I	I	[52]
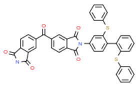	I	PS	[52]
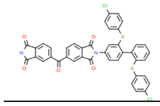	I	PS	[52]

Continued on next page

MONOMER	*Experimental*	*Predicted*	**Ref**
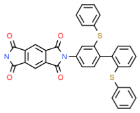	I	S	[52]
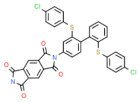	I	I	[52]
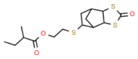	S	PS	[42]
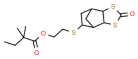	S	S	[42]
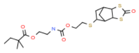	S	S	[42]
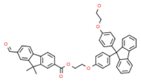	S	S	[44]
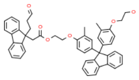	S	S	[44]
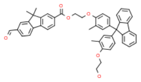	S	S	[44]
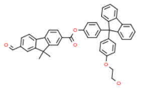	S	S	[44]
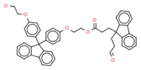	S	S	[44]
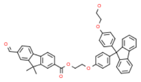	S	S	[44]
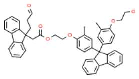	S	S	[44]
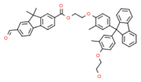	S	S	[44]
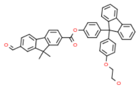	S	S	[44]
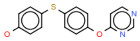	I	I	[37]
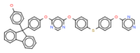	S	S	[37]
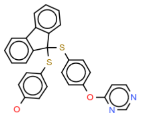	S	S	[37]
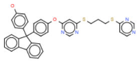	S	S	[37]
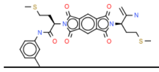	PS	PS	[10]

Continued on next page

Table S11 – *Continued from previous page*

MONOMER	*Experimental*	*Predicted*	**Ref**
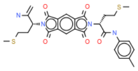	PS	PS	[10]
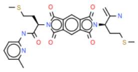	PS	PS	[10]
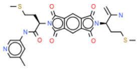	PS	PS	[10]
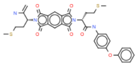	PS	PS	[10]
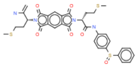	PS	PS	[10]
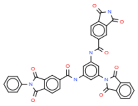	S	S	[3]
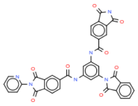	S	S	[3]
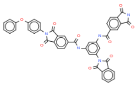	PS	S	[3]
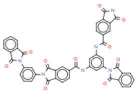	S	S	[3]
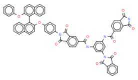	S	S	[3]
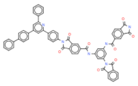	S	S	[3]
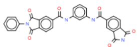	S	S	[3]
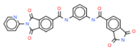	S	S	[3]
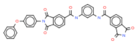	PS	S	[3]
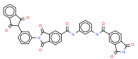	S	S	[3]
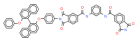	S	S	[3]
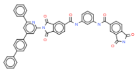	S	S	[3]
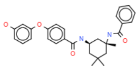	PS	PS	[66]
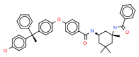	PS	S	[66]
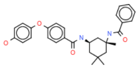	PS	PS	[66]
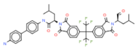	PS	PS	[47]
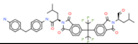	PS	PS	[47]

Continued on next page

Table S11 – *Continued from previous page*

**MONOMER**	*Experimental*	*Predicted*	**Ref**
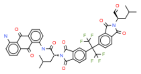	PS	PS	[47]
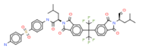	PS	PS	[47]
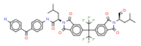	PS	PS	[47]
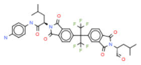	PS	PS	[47]
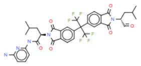	PS	PS	[47]
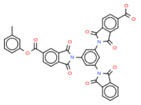	PS	PS	[2]
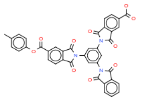	PS	PS	[2]
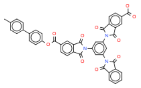	PS	PS	[2]
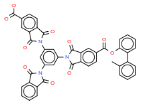	PS	PS	[2]
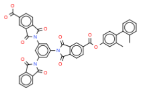	PS	PS	[2]
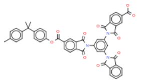	PS	PS	[2]
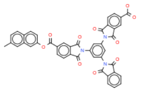	PS	PS	[2]
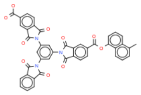	PS	PS	[2]
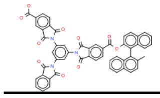	PS	PS	[2]

Table S12: The table lists the experimental and predicted solubility classes (S - soluble, PS - partially soluble/swelling/soluble on heating, I - insoluble) for different polymers in DMSO. Predictions for the RF model are reported.

MONOMER	*Experimental*	*Predicted*	Ref
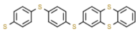	I	I	[51]
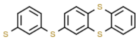	I	I	[51]
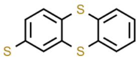	I	I	[51]
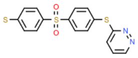	S	S	[63]
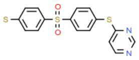	S	S	[63]
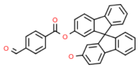	PS	PS	[45]
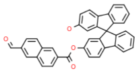	PS	PS	[45]
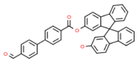	PS	PS	[45]
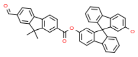	PS	PS	[45]
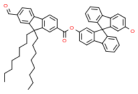	PS	PS	[45]
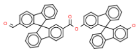	PS	S	[45]
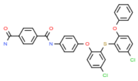	S	S	[15]
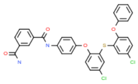	S	S	[15]
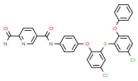	S	S	[15]
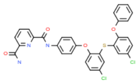	S	S	[15]
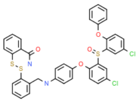	S	S	[15]
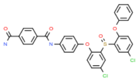	S	S	[15]
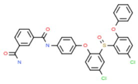	S	S	[15]
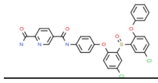	S	S	[15]

Continued on next page

Table S12 – *Continued from previous page*

MONOMER	*Experimental*	*Predicted*	**Ref**
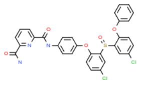	S	S	[15]
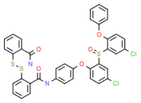	S	S	[15]
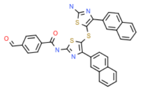	S	S	[16]
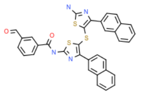	S	S	[16]
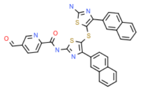	S	S	[16]
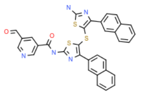	S	S	[16]
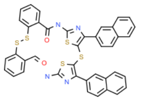	S	S	[16]
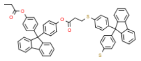	S	S	[38]
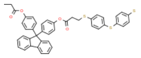	PS	PS	[38]
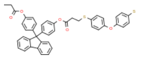	S	S	[38]
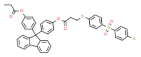	S	S	[38]
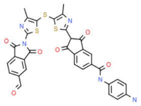	PS	S	[48]
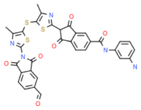	PS	S	[48]
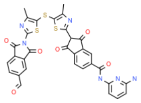	S	S	[48]
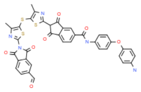	S	S	[48]
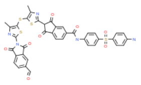	PS	S	[48]
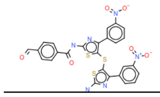	S	S	[18]

Continued on next page

MONOMER	*Experimental*	*Predicted*	Ref
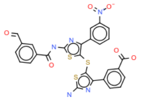	S	S	[18]
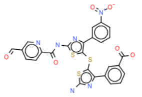	S	S	[18]
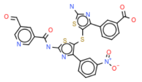	S	S	[18]
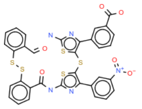	S	S	[18]
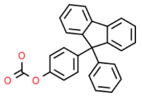	S	S	[46]
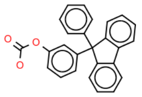	S	S	[46]
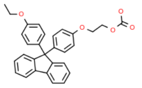	PS	PS	[46]
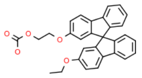	PS	S	[46]
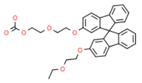	PS	PS	[46]
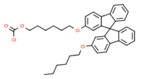	PS	PS	[46]
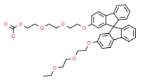	PS	PS	[46]
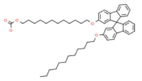	PS	PS	[46]
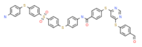	S	S	[65]
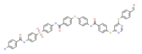	S	S	[65]
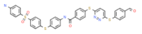	S	S	[65]
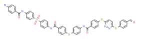	S	S	[65]
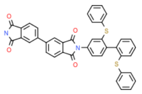	PS	PS	[52]
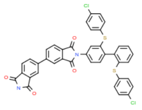	PS	I	[52]
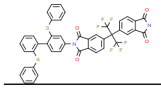	I	PS	[52]

Continued on next page

Table S12 – *Continued from previous page*

MONOMER	*Experimental*	*Predicted*	**Ref**
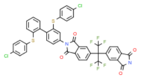	I	I	[52]
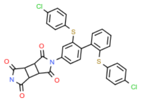	I	I	[52]
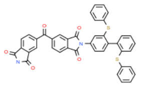	I	I	[52]
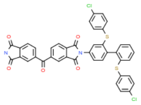	I	I	[52]
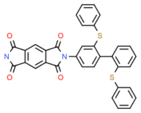	I	I	[52]
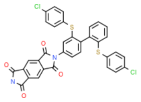	I	I	[52]
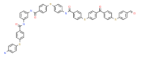	PS	PS	[64]
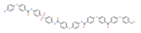	PS	PS	[64]
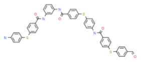	PS	PS	[64]
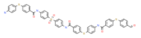	PS	PS	[64]
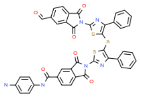	PS	PS	[19]
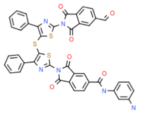	S	S	[19]
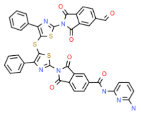	S	S	[19]
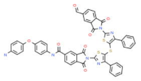	S	S	[19]
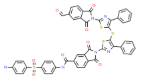	S	S	[19]
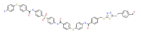	S	PS	[35]
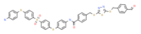	S	S	[35]
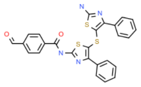	S	S	[17]
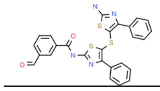	S	S	[17]

Continued on next page

Table S12 – *Continued from previous page*

MONOMER	*Experimental*	*Predicted*	Ref
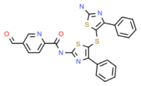	S	S	[17]
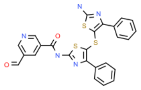	S	S	[17]
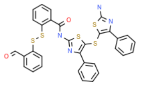	S	S	[17]
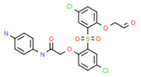	S	S	[20]
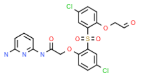	S	S	[20]
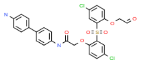	S	S	[20]
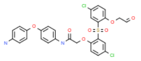	S	S	[20]
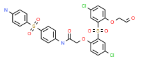	S	S	[20]
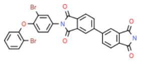	I	S	[5]
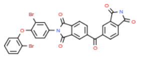	S	S	[5]
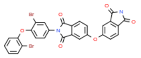	S	S	[5]
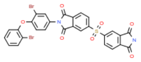	S	S	[5]
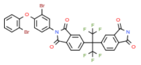	S	S	[5]
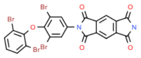	S	S	[5]
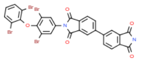	I	I	[5]
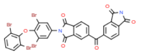	S	S	[5]
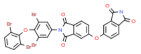	S	S	[5]
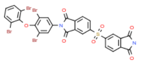	S	S	[5]
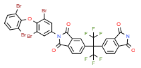	S	S	[5]
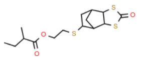	S	S	[42]
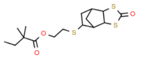	S	S	[42]
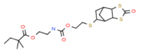	S	S	[42]
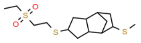	S	S	[42]
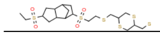	S	S	[50]

Continued on next page

Table S12 – *Continued from previous page*

**MONOMER**	*Experimental*	*Predicted*	**Ref**
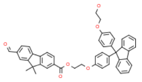	I	I	[50]
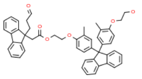	S	S	[44]
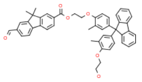	I	I	[44]
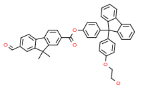	S	I	[44]
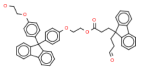	S	S	[44]
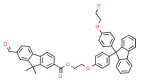	I	I	[44]
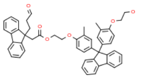	S	S	[44]
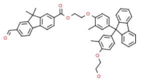	I	I	[44]
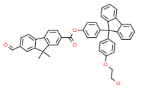	S	I	[44]
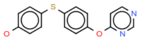	I	S	[44]
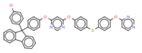	I	PS	[37]
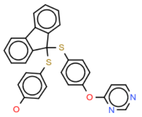	I	I	[37]
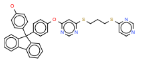	PS	I	[37]
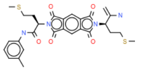	PS	PS	[37]
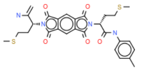	PS	PS	[10]
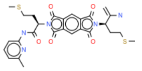	PS	PS	[10]
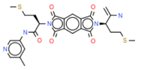	PS	PS	[10]
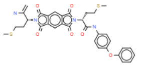	PS	PS	[10]
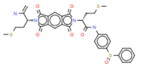	PS	PS	[10]
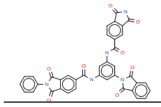	S	PS	[10]

Continued on next page

Table S12 – *Continued from previous page*

MONOMER	*Experimental*	*Predicted*	**Ref**
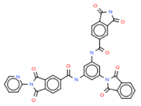	PS	PS	[3]
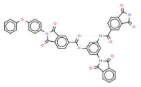	PS	PS	[3]
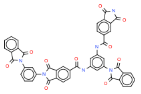	S	PS	[3]
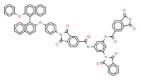	S	S	[3]
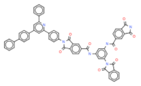	S	S	[3]
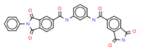	PS	PS	[3]
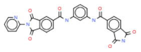	PS	PS	[3]
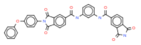	PS	PS	[3]
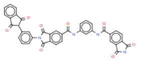	PS	PS	[3]
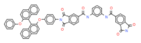	S	S	[3]
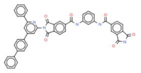	S	PS	[3]
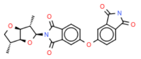	S	S	[3]
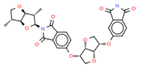	S	PS	[56]
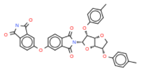	PS	PS	[56]
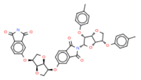	S	S	[56]
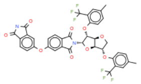	S	S	[56]
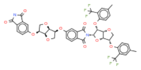	S	S	[56]
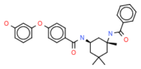	PS	PS	[56]
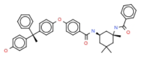	PS	PS	[66]
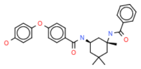	PS	PS	[66]
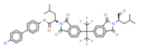	PS	PS	[66]
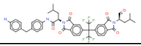	PS	PS	[47]

Continued on next page

Table S12 – *Continued from previous page*

**MONOMER**	*Experimental*	*Predicted*	**Ref**
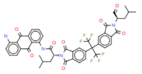	PS	PS	[47]
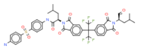	PS	S	[47]
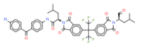	PS	PS	[47]
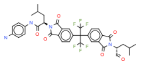	PS	PS	[47]
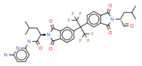	PS	PS	[47]
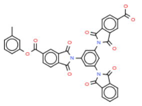	PS	PS	[47]
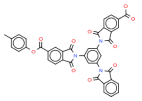	PS	PS	[2]
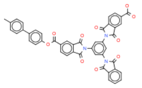	PS	PS	[2]
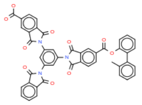	PS	PS	[2]
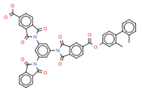	PS	PS	[2]
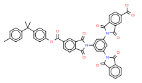	PS	PS	[2]
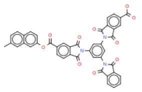	PS	PS	[2]
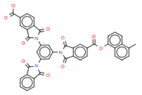	PS	PS	[2]
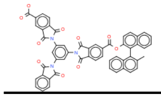	PS	PS	[2]

Table S13: The table lists the experimental and predicted refractive indices measured at given wavelengths for different polymers. Refractive indices for polymers at wavelengths other than 589 nm were taken from multiple references [51, 21, 16, 8, 57, 55, 19, 35, 42, 24, 54]. Here, the the refractive indices are predicted using the polarizabilities derived from DFT calculations and densities predicted using a QSPR model.

MONOMER	n_{DFT}	λ	n_{pred}
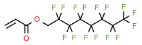	1.34	589	1.27
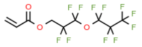	1.35	589	1.27
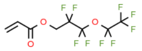	1.35	589	1.29
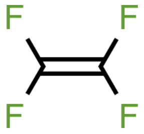	1.35	589	1.21
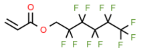	1.36	589	1.28
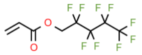	1.36	589	1.29
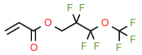	1.36	589	1.30
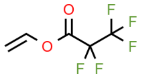	1.36	589	1.30
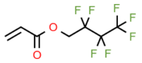	1.37	589	1.31
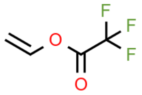	1.38	589	1.34
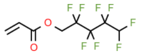	1.38	589	1.31
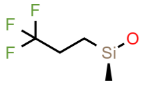	1.38	589	1.41
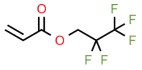	1.39	589	1.34
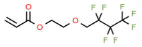	1.39	589	1.35
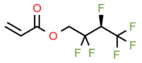	1.39	589	1.33
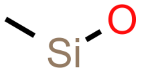	1.40	589	1.53
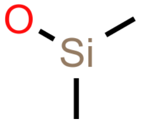	1.40	589	1.42
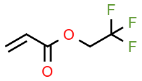	1.41	589	1.38
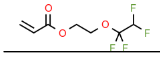	1.41	589	1.38

Continued on next page

Table S13 – *Continued from previous page*

MONOMER	n_{DFT}	λ	n_{pred}
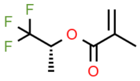	1.42	589	1.44
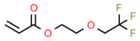	1.42	589	1.42
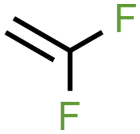	1.42	589	1.36
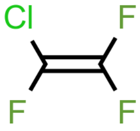	1.43	589	1.26
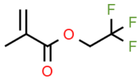	1.44	589	1.41
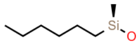	1.44	589	1.47
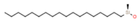	1.44	589	1.51
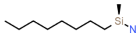	1.45	589	1.58
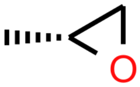	1.45	589	1.55
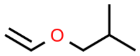	1.45	589	1.53
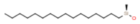	1.45	589	1.52
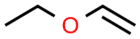	1.45	589	1.51
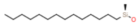	1.46	589	1.51
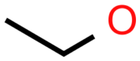	1.46	589	1.48
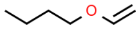	1.46	589	1.59
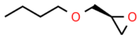	1.46	589	1.56
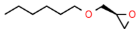	1.46	589	1.59
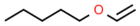	1.46	589	1.60
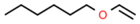	1.46	589	1.61
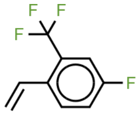	1.46	589	1.51
	1.46	589	1.62
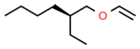	1.46	589	1.54
	1.46	589	1.61
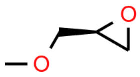	1.46	589	1.48
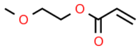	1.46	589	1.52
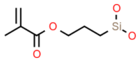	1.46	589	1.48
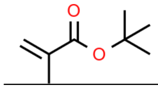	1.46	589	1.51

Continued on next page

Table S13 – *Continued from previous page*

MONOMER	n_{DFT}	λ	n_{pred}
	1.46	589	1.61
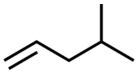	1.47	589	1.54
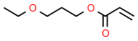	1.47	589	1.56
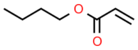	1.47	589	1.54
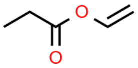	1.47	589	1.49
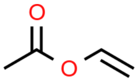	1.47	589	1.49
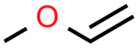	1.47	589	1.48
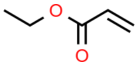	1.47	589	1.52
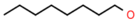	1.47	589	1.55
	1.47	589	1.59
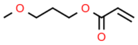	1.47	589	1.51
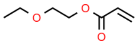	1.47	589	1.52
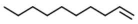	1.47	589	1.59
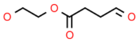	1.47	589	1.40
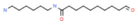	1.48	589	1.58
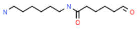	1.48	589	1.55
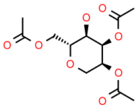	1.48	589	1.44
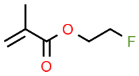	1.48	589	1.50
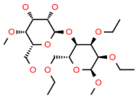	1.48	589	1.47
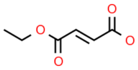	1.48	589	1.48
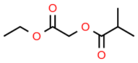	1.49	589	1.41
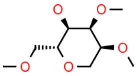	1.50	589	1.46
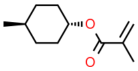	1.50	589	1.49
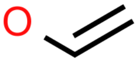	1.50	589	1.50
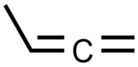	1.50	589	1.70
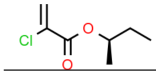	1.50	589	1.53

Continued on next page

Table S13 – *Continued from previous page*

MONOMER	n_{DFT}	λ	n_{pred}
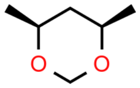	1.50	589	1.48
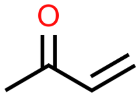	1.50	589	1.56
	1.50	589	1.54
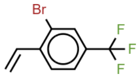	1.50	589	1.61
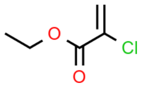	1.50	589	1.52
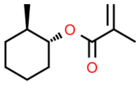	1.50	589	1.49
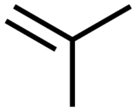	1.51	589	1.56
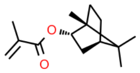	1.51	589	1.51
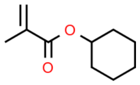	1.51	589	1.47
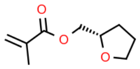	1.51	589	1.48
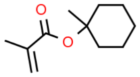	1.51	589	1.50
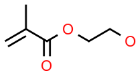	1.51	589	1.49
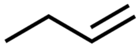	1.51	589	1.56
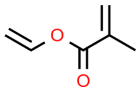	1.51	589	1.55
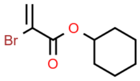	1.54	589	1.58
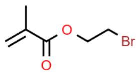	1.54	589	1.43
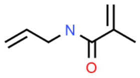	1.55	589	1.61
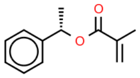	1.55	589	1.55
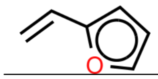	1.55	589	1.60

Continued on next page

Table S13 – *Continued from previous page*

MONOMER	n_{DFT}	λ	n_{pred}
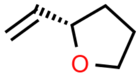	1.55	589	1.53
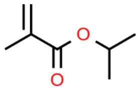	1.55	589	1.48
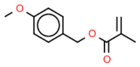	1.55	589	1.55
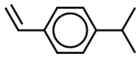	1.55	589	1.69
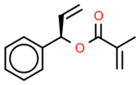	1.56	589	1.63
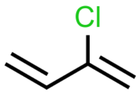	1.56	589	1.65
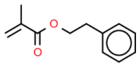	1.56	589	1.62
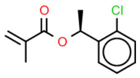	1.56	589	1.59
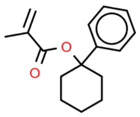	1.56	589	1.55
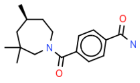	1.57	589	1.63
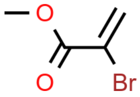	1.57	589	1.46
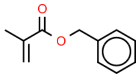	1.57	589	1.61
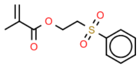	1.57	589	1.56
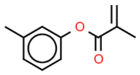	1.57	589	1.62
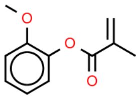	1.57	589	1.59
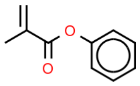	1.57	589	1.60
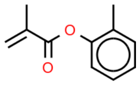	1.57	589	1.60
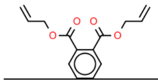	1.57	589	1.59

Continued on next page

Table S13 – *Continued from previous page*

MONOMER	n_{DFT}	λ	n_{pred}
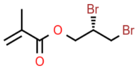	1.57	589	1.52
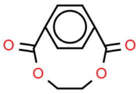	1.58	589	1.51
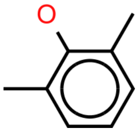	1.58	589	1.56
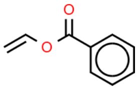	1.58	589	1.64
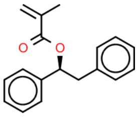	1.58	589	1.64
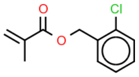	1.58	589	1.60
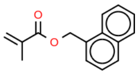	1.63	589	1.69
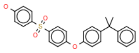	1.63	589	1.68
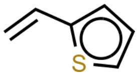	1.64	589	1.72
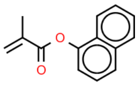	1.64	589	1.69
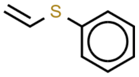	1.66	589	1.73
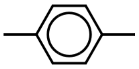	1.67	589	1.57
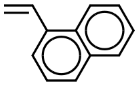	1.68	589	1.81
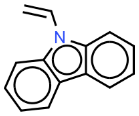	1.68	589	1.81
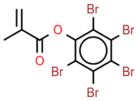	1.71	589	1.52
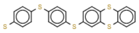	1.79	633	1.75
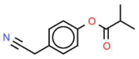	1.51	589	1.60
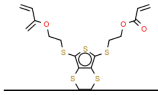	1.64	637	1.65

Continued on next page

Table S13 – *Continued from previous page*

**MONOMER**	n_{DFT}	λ	n_{pred}
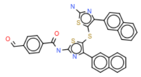	1.76	633	1.85
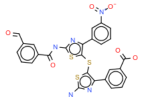	1.75	633	1.78
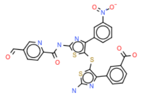	1.76	633	1.81
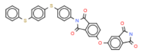	1.71	633	1.80
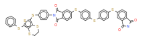	1.76	633	1.81
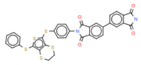	1.75	633	1.81
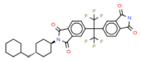	1.58	1324	1.62
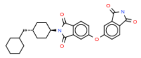	1.51	1324	1.61
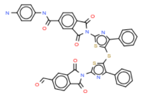	1.74	633	1.78
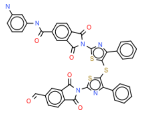	1.74	633	1.78
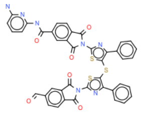	1.74	633	1.78
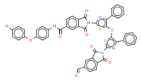	1.74	633	1.75
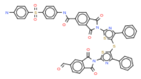	1.75	633	1.74
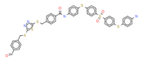	1.73	633	1.80
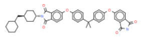	1.57	1324	1.63
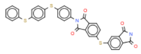	1.70	1310	1.77
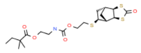	1.59	589	1.58
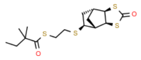	1.64	589	1.58
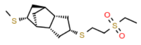	1.61	589	1.61
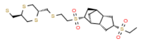	1.62	589	1.55
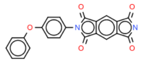	1.65	1324	1.62
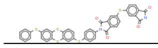	1.72	1310	1.77

Continued on next page

Table S13 – *Continued from previous page*

**MONOMER**	n_{DFT}	λ	n_{pred}
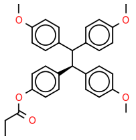	1.60	633	1.66
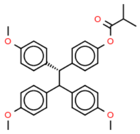	1.60	633	1.66
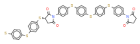	1.71	633	1.75
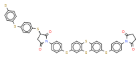	1.73	633	1.77
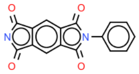	1.69	1320	1.58
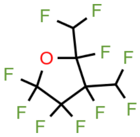	1.52	589	1.33
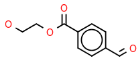	1.58	589	1.64
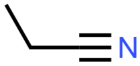	1.52	589	1.56
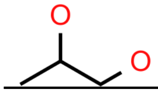	1.45	589	1.39

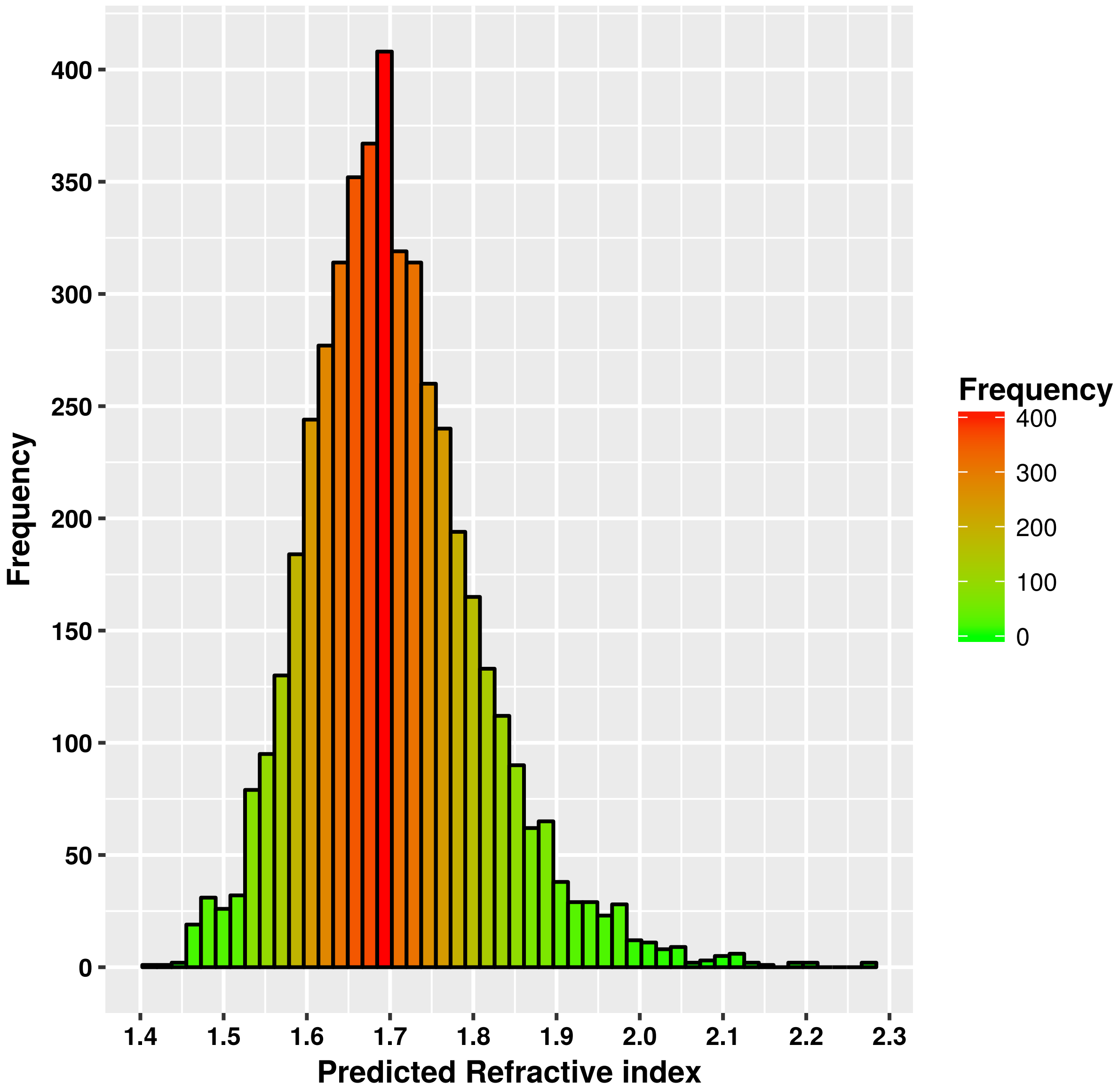

Figure S1: Plot shows the histogram of the predicted refractive indices for the different monomers emerging from the de novo runs.

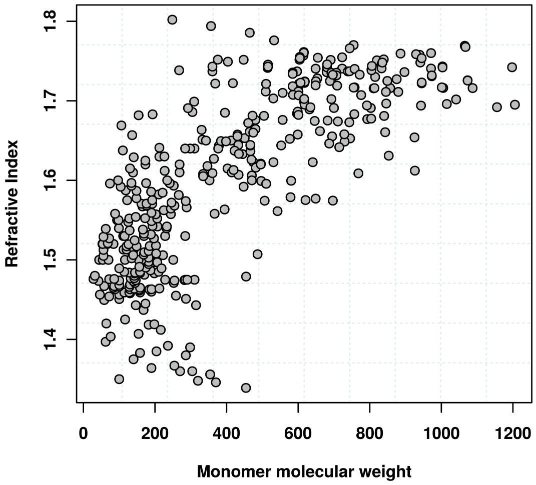

Figure S2: Plot shows the scatter plot of the molecular weights vs the experimental refractive indices (taken from literature) of various polymers.

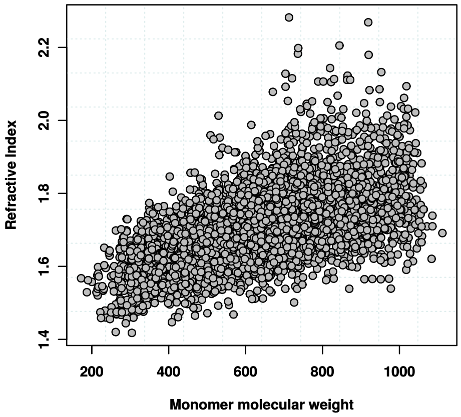

Figure S3: Plot shows the scatter plot of the molecular weights vs the predicted refractive indices of different monomers emerging from the de novo runs.

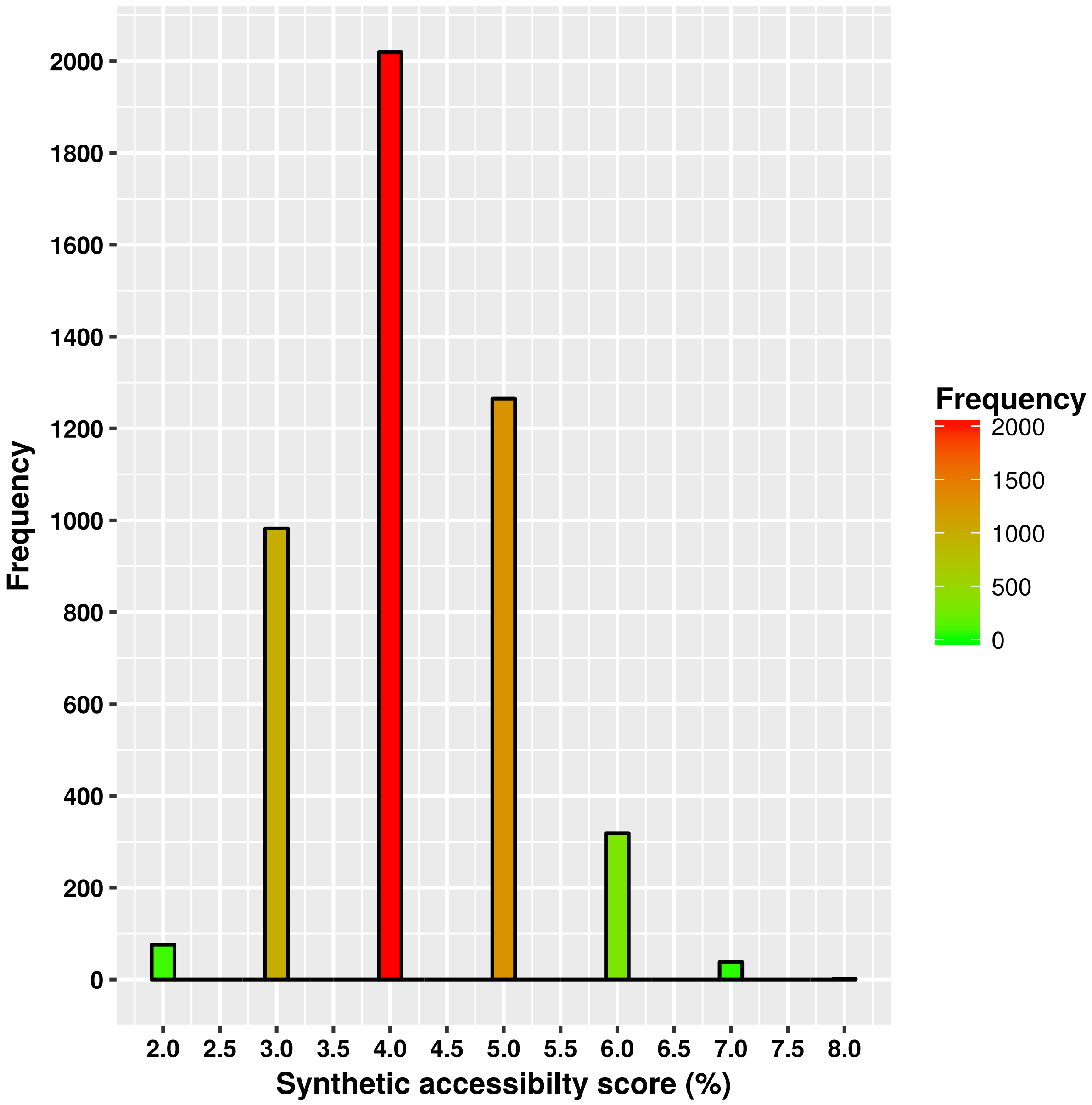

Figure S4: Plot shows the histogram of the synthetic accessibility scores for the different monomers emerging from the de novo *runs.*

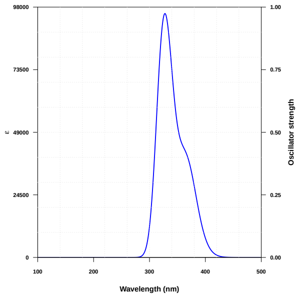

(a) M0001

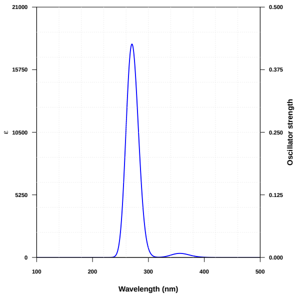

(b) M0002

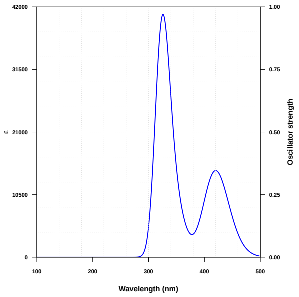

(c) M0003

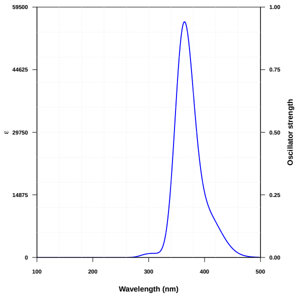

(d) M0004

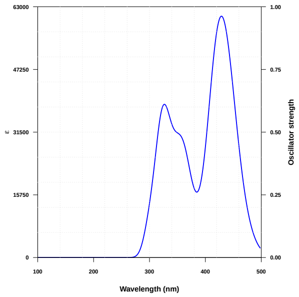

(e) M0005

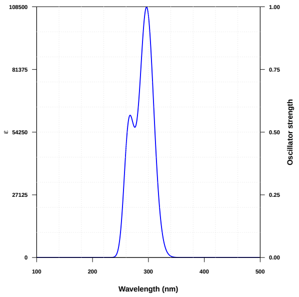

(f) M0006

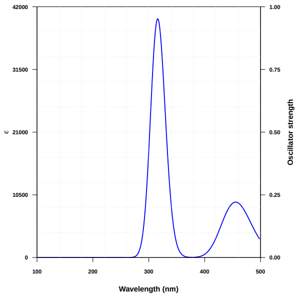

(g) M0007

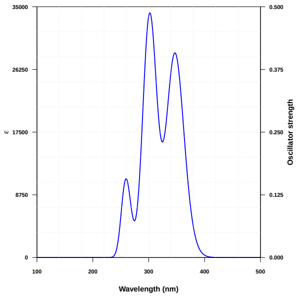

(h) M0008

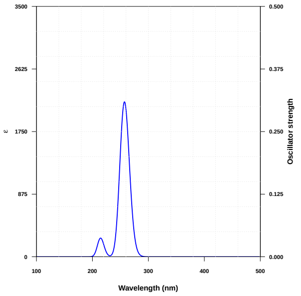

(i) M0009

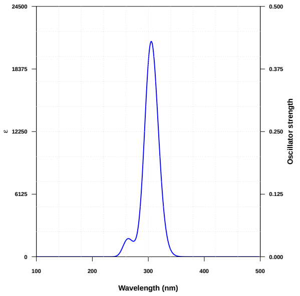

(j) M0010

Figure S5: Calculated UV-VIS spectra for different polymers (see main article).

Table S14: The table lists the experimental (n_{exp}) and predicted refractive indices (n_{pred}) for different polymers. The n were estimated using DFT-based polarizability values at 589 nm and reported experimental densities.

MONOMER	n_{exp}	ρ_{qspr}	ρ_{exp}	n_{pred}
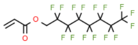	1.34	1.40	1.42	1.27
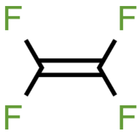	1.35	1.37	0.91	1.14
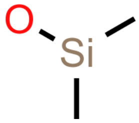	1.40	0.96	0.89	1.39
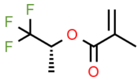	1.42	1.38	0.88	1.27
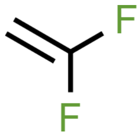	1.42	1.44	1.13	1.27
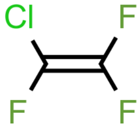	1.43	1.36	1.66	1.32
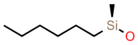	1.44	0.94	1.60	1.93
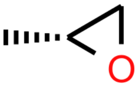	1.45	1.25	1.39	1.62
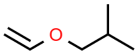	1.45	1.03	1.25	1.67
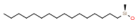	1.45	0.97	1.26	1.73
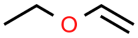	1.45	1.03	1.17	1.59
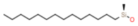	1.46	0.95	1.34	1.79
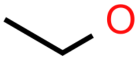	1.46	1.11	1.02	1.44
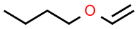	1.46	1.13	1.03	1.53
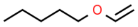	1.46	1.13	1.04	1.70
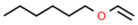	1.46	1.13	1.13	1.61
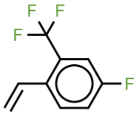	1.46	1.38	1.17	1.42
	1.46	1.13	1.15	1.63
	1.46	1.10	1.12	1.62
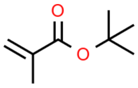	1.46	1.09	1.21	1.58
	1.46	1.10	1.10	1.62
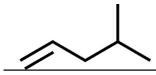	1.47	0.93	1.11	1.68

Continued on next page

Table S14 – *Continued from previous page*

MONOMER	n_{exp}	ρ_{qspr}	ρ_{exp}	n_{pred}
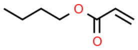	1.47	1.15	1.05	1.48
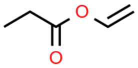	1.47	1.13	1.18	1.51
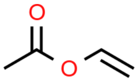	1.47	1.20	1.08	1.44
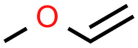	1.47	1.03	1.06	1.50
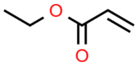	1.47	1.18	1.01	1.43
	1.47	0.99	0.93	1.54
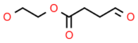	1.47	1.10	1.10	1.39
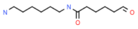	1.48	1.14	0.93	1.43
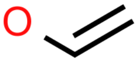	1.50	1.13	1.30	1.40
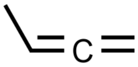	1.50	1.07	1.04	1.68
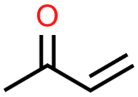	1.50	1.15	0.93	1.43
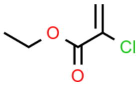	1.50	1.37	0.91	1.33
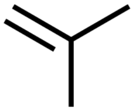	1.51	0.94	0.88	1.52
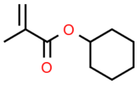	1.51	1.01	0.89	1.41
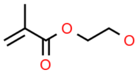	1.51	1.18	0.85	1.34
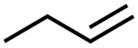	1.51	0.93	0.91	1.54
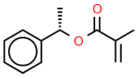	1.55	1.09	1.03	1.51
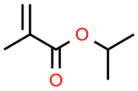	1.55	1.04	1.07	1.49
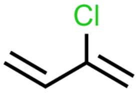	1.56	1.31	1.24	1.61
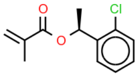	1.56	1.26	1.92	2.04
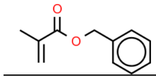	1.57	1.19	1.25	1.65

Continued on next page

Table S14 – *Continued from previous page*

MONOMER	n_{exp}	ρ_{qspr}	ρ_{exp}	n_{pred}
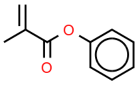	1.57	1.18	1.45	1.78
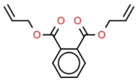	1.57	1.23	1.05	1.49
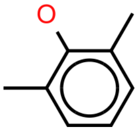	1.58	1.07	2.00	2.38
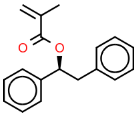	1.58	1.19	1.43	1.82
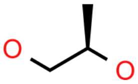	1.45	1.16	1.01	1.39
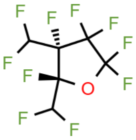	1.34	1.45	2.03	1.33
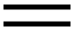	1.48	0.91	0.85	1.76
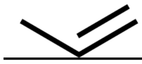	1.47	0.91	0.85	1.88

Table S15: Cases where large deviations between QSPR and DFT estimates for n are observed.

Monomer	n_{qspr}	n_{DFT}
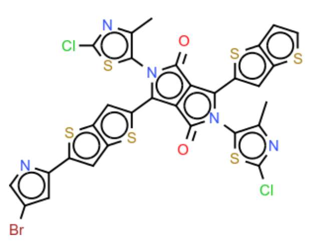	2.14±0.15	5.79
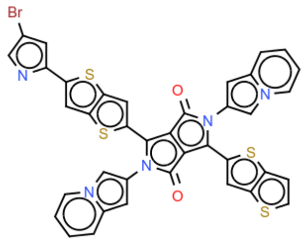	1.99±0.12	5.22
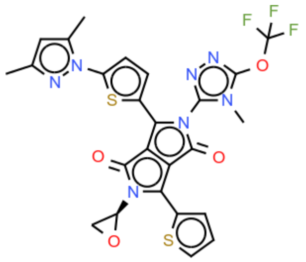	1.89±0.08	2.37
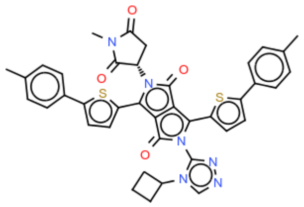	1.94±0.08	2.78
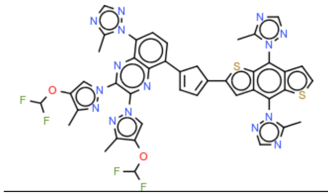	1.93±0.09	2.38

References

[1] O. Aptula, Aynur, R. Kühne, R.-U. Ebert, T. Cronin, Mark, T. Netzeva, and G. Schüürmann. Modeling discrimination between antibacterial and non-antibacterial activity based on 3d molecular descriptors. *Mol. Inf.*, 22(1):113–128, 2003.

[2] H. Behniafar, B. Akhlaghinia, and S. Habibian. Synthesis and characterization of new soluble and thermally stable poly(ester-imide)s derived from n-[3,5-bis(n-trimellitoyl)phenyl]phthalimide and various bisphenols. *European Polymer Journal*, 41(5):1071–1078, 2005.

[3] H. Behniafar and S. Haghighat. Preparation and properties of aromatic poly(amide-imide)s derived from n-[3,5-bis(3,4-dicarboxybenzamido)phenyl]phthalimide dianhydride. *European Polymer Journal*, 42(12):3236–3247, 2006.

[4] J. Bicerano. *Prediction of Polymer Properties*. CRC Press, 3 edition, 2002.

[5] J.-C. Chen, Y.-T. Liu, C.-M. Leu, H.-Y. Liao, W.-C. Lee, and T.-M. Lee. Synthesis and properties of organosoluble polyimides derived from 2,2′-dibromo- and 2,2′,6,6′-tetrabromo-4,4′-oxydianilines. *Journal of Applied Polymer Science*, 117(2):1144–1155, 2010.

[6] P. R. Duchowicz, S. E. Fioressi, D. E. Bacelo, L. M. Saavedra, A. P. Toropova, and A. A. Toropov. QSPR studies on refractive indices of structurally heterogeneous polymers. *Chemometrics and Intelligent Laboratory Systems*, 140:86–91, 2015.

[7] N. Fukuzaki, T. Higashihara, S. Ando, and M. Ueda. Synthesis and characterization of highly refractive polyimides derived from thiophene-containing aromatic diamines and aromatic dianhydrides. *Macromolecules*, 43(4):1836–1843, 2010.

[8] J. gang Liu, Y. Nakamura, Y. Shibasaki, S. Ando, and M. Ueda. Synthesis and characterization of high refractive index polyimides derived from 4,4′-(p-phenylenedisulfanyl)dianiline and various aromatic tetracarboxylic dianhydrides. *Polymer Journal*, 39(6):543–550, 2007.

[9] J. gang Liu, Y. Nakamura, Y. Suzuki, Y. Shibasaki, S. Ando, and M. Ueda. Highly refractive and transparent polyimides derived from 4,4‘-[m-sulfonylbis(phenylenesulfanyl)]diphthalic anhydride and various sulfur-containing aromatic diamines. *Macromolecules*, 40(22):7902–7909, 2007.

[10] A. R. Hajipour, S. Zahmatkesh, A. Zarei, L. Khazdooz, and A. E. Ruoho. Synthesis and characterization of novel optically active poly(amide–imide)s via direct amidation. *European Polymer Journal*, 41(10):2290–2296, 2005.

[11] I. Hamerton, B. J. Howlin, and G. Kamyszek. Predicting glass transition temperatures of polyarylethersulphones using qspr methods. *PLOS ONE*, 7(6):1–8, 2012.

[12] S. Helmstetter, T. Badur, and N. Hampp. High-refractive quinolinone-based polymers for ophthalmic devices. *Journal of Polymer Research*, 23(12), 2016.

[13] X. Huang, W. Huang, L. Fu, and D. Yan. Synthesis and characterization of thioether-containing polyimides with high refractive indices. *Journal of Polymer Research*, 19(2), 2012.

[14] S. P. P. Inc. Refractive index of polymers by index. http://scientificpolymer.com/technical-library/refractive-index-of-polymers-by-index/. Accessed: 2017-02-11.

[15] A. Javadi, E. Abouzari-Lotf, S. Mehdipour-Ataei, M. Zakeri, M. M. Nasef, A. Ahmad, and A. Ripin. High refractive index materials: A structural property comparison of sulfide- and sulfoxide-containing polyamides. *Journal of Polymer Science Part A: Polymer Chemistry*, 53(24):2867–2877, 2015.

[16] A. Javadi, Z. Najjar, S. Bahadori, V. Vatanpour, A. Malek, E. Abouzari-Lotf, and A. Shockravi. High refractive index and low-birefringence polyamides containing thiazole and naphthalene units. *RSC Adv.*, 5(111):91670–91682, 2015.

[17] A. Javadi, A. Shockravi, M. Kamali, A. Rafieimanesh, and A. M. Malek. Solution processable polyamides containing thiazole units and thioether linkages with high optical transparency, high refractive index, and low birefringence. *Journal of Polymer Science Part A: Polymer Chemistry*, 51(16):3505–3515, 2013.

[18] A. Javadi, A. Shockravi, M. Koohgard, A. Malek, F. A. Shourkaei, and S. Ando. Nitro-substituted polyamides: A new class of transparent and highly refractive materials. *European Polymer Journal*, 66:328–341, 2015.

[19] A. Javadi, A. Shockravi, A. Rafieimanesh, A. Malek, and S. Ando. Synthesis and structure-property relationships of novel thiazole-containing poly(amide imide)s with high refractive indices and low birefringences. *Polymer International*, 64(4):486–495, 2014.

[20] H. Kiani, M. M. Nasef, A. Javadi, E. Abouzari-Lotf, and F. Nemati. Highly refractive, transparent, and solution processable polyamides based on a noncoplanar ortho-substituted sulfonyl-bridged diacid monomer containing chlorine side groups. *Journal of Polymer Research*, 20(9), 2013.

[21] H. Kim, H. Yeo, M. Goh, B.-C. Ku, J. R. Hahn, and N.-H. You. Preparation of UV-curable acryl resin for high refractive index based on 1,5-bis(2-acryloylenethyl)-3,4-ethylenedithiothiophene. *European Polymer Journal*, 75:303–309, 2016.

[22] J.-S. Kim, B.-S. Cho, J.-O. Kweon, and S.-T. Noh. Preparation and properties of UV-curable di-functional sulfur-containing thioacrylate and thiourethane acrylate monomers with high refractive indices. *Progress in Organic Coatings*, 77(11):1695–1700, 2014.

[23] D. V. Krevelen, R. by, and K. T. Nijenhuis, editors. *Properties of Polymers (Fourth Edition)*. Elsevier, Amsterdam, fourth edition edition, 2009.

[24] Z. Li, J.-G. Liu, and S.-Y. Yang. Synthesis and characterization of thioether and pyridine-bridged aromatic polyimides with high refractive indices and high glass transition temperatures. *High Performance Polymers*, 22(4):468–482, 2010.

[25] J.-g. Liu, Y. Nakamura, Y. Shibasaki, S. Ando, and M. Ueda. High refractive index polyimides derived from 2,7-bis(4-aminophenylenesulfanyl)thianthrene and aromatic dianhydrides. *Macromolecules*, 40(13):4614–4620, 2007.

[26] J.-G. Liu, Y. Nakamura, Y. Shibasaki, S. Ando, and M. Ueda. Synthesis and characterization of highly refractive polyimides from 4,4′-thiobis[(p-phenylenesulfanyl)aniline] and various aromatic tetracarboxylic dianhydrides. *Journal of Polymer Science Part A: Polymer Chemistry*, 45(23):5606–5617, 2007.

[27] J.-G. Liu, Y. Nakamura, C. A. Terraza, Y. Shibasaki, S. Ando, and M. Ueda. Highly refractive polyimides derived from 2,8-bis(p-aminophenylenesulfanyl)dibenzothiophene and aromatic dianhydrides. *Macromolecular Chemistry and Physics*, 209(2):195–203, 2008.

[28] W. Liu and C. Cao. Artificial neural network prediction of glass transition temperature of polymers. *Colloid and Polymer Science*, 287(7):811–818, 2009.

[29] W. Liu, P. Yi, and Z. Tang. QSPR models for various properties of polymethacrylates based on quantum chemical descriptors. *QSAR & Combinatorial Science*, 25(10):936–943, 2006.

[30] E. K. Macdonald, J. C. Lacey, I. Ogura, and M. P. Shaver. Aromatic polyphosphonates as high refractive index polymers. *European Polymer Journal*, 87:14–23, 2017.

[31] S. Mallakpour and E. Kowsari. Thermally stable and optically active poly(amide-imide)s derived from 4,4'–(hexafluoroisopropylidene)-n,n'-bis-(phthaloyl-l-methionine) diacid chloride and various aromatic diamines: Synthesis and characterization. *Polymer Bulletin*, 57(2):169–178, 2006.

[32] S. Mallakpour and M. H. Shahmohammadi. Microwave-promoted rapid synthesis of new optically active poly(amide imide)s derived fromN,n?-(pyromellitoyl)-bis-l-isoleucine diacid chloride and aromatic diamines. *Journal of Applied Polymer Science*, 92(2):951–959, 2004.

[33] S. E. Mallakpour, A.-R. Hajipour, and S. Khoee. Rapid synthesis of optically active poly(amide–imide)s by direct polycondensation of aromatic dicarboxylic acid with aromatic diamines. *European Polymer Journal*, 38(10):2011–2016, 2002.

[34] J. E. Mark. *The Polymer Data Handbook*. Oxford University Press, 2 edition, 2009.

[35] Z. min Li, G. Zhang, D. sheng Li, and J. Yang. Polyamides containing thiadiazole units: Synthesis and optical properties. *Chinese Journal of Polymer Science*, 32(3):292–304, 2014.

[36] G. ming Yan, G. Zhang, H. hao Ren, Y. Li, and J. Yang. Synthesis and characterization of semiaromatic polyamides with dicyclohexane units. *RSC Adv.*, 6(80):76490–76497, 2016.

[37] K. Nakabayashi, T. Imai, M.-C. Fu, S. Ando, T. Higashihara, and M. Ueda. Synthesis and characterization of poly(phenylene thioether)s containing pyrimidine units exhibiting high transparency, high refractive indices, and low birefringence. *J. Mater. Chem. C*, 3(27):7081–7087, 2015.

[38] K. Nakabayashi, T. Imai, M.-C. Fu, S. Ando, T. Higashihara, and M. Ueda. Poly(phenylene thioether)s with fluorene-based cardo structure toward high transparency, high refractive index, and low birefringence. *Macromolecules*, 49(16):5849–5856, 2016.

[39] Y. Nakagawa, T. Ogura, T. Higashihara, and M. Ueda. Optically transparent sulfur-containing semi-alicyclic polyimide with high refractive index. *Chemistry Letters*, 39(4):392–393, 2010.

[40] Y. Nakagawa, Y. Suzuki, T. Higashihara, S. Ando, and M. Ueda. Synthesis of highly refractive poly(phenylene thioether) derived from 2,4-dichloro-6-alkylthio-1,3,5-triazines and aromatic dithiols. *Macromolecules*, 44(23):9180–9186, 2011.

[41] Y. Nakagawa, Y. Suzuki, T. Higashihara, S. Ando, and M. Ueda. Synthesis of highly refractive poly(phenylene thioether)s containing a binaphthyl or diphenylfluorene unit. *Polymer Chemistry*, 3(9):2531, 2012.

[42] R. Okutsu, S. Ando, and M. Ueda. Sulfur-containing poly(meth)acrylates with high refractive indices and high abbe's numbers. *Chemistry of Materials*, 20(12):4017–4023, 2008.

[43] R. Okutsu, Y. Suzuki, S. Ando, and M. Ueda. Poly(thioether sulfone) with high refractive index and high abbe's number. *Macromolecules*, 41(16):6165–6168, 2008.

[44] S. Seesukphronrarak, S. Kawasaki, K. Kobori, and T. Takata. Fluorene-rich high performance polyesters: Synthesis and characterization of 9,9-fluorenylidene and 2,7-fluorenylene-based polyesters with excellent optical property. *Journal of Polymer Science Part A: Polymer Chemistry*, 46(7):2549–2556, 2008.

[45] R. Seto, T. Kojima, K. Hosokawa, Y. Koyama, G. ichi Konishi, and T. Takata. Synthesis and property of 9,9′-spirobifluorene-containing aromatic polyesters as optical polymers with high refractive index and low birefringence. *Polymer*, 51(21):4744–4749, 2010.

[46] R. Seto, T. Sato, T. Kojima, K. Hosokawa, Y. Koyama, G.-I. Konishi, and T. Takata. 9,9′-spirobifluorene-containing polycarbonates: Transparent polymers with high refractive index and low birefringence. *Journal of Polymer Science Part A: Polymer Chemistry*, 48(16):3658–3667, 2010.

[47] A.-R. H. Shadpour E Mallakpour and S. Khoee. Synthesis and characterization of novel optically active poly(amide-imide)s. *Polymer International*, 48(11):11331140, 1999.

[48] A. Shockravi, A. Javadi, M. Kamali, and S. Hajavi. Highly refractive and organo-soluble poly(amide imide)s based on 5,5′-thiobis(2-amino-4-methyl-thiazole): Synthesis and characterization. *Journal of Applied Polymer Science*, 125(2):1521–1529, 2012.

[49] Y. Suzuki, J. gang Liu, Y. Nakamura, Y. Shibasaki, S. Ando, and M. Ueda. Synthesis of highly refractive and transparent polyimides derived from 4,4′-[p-sulfonylbis(phenylenesulfanyl)]diphthalic anhydride and various sulfur-containing aromatic diamines. *Polymer Journal*, 40(5):414–420, 2008.

[50] Y. Suzuki, T. Higashihara, S. Ando, and M. Ueda. Synthesis and characterization of high refractive index and high abbe's number poly(thioether sulfone)s based on tricyclo[5.2.1.02,6]decane moiety. *Macromolecules*, 45(8):3402–3408, 2012.

[51] Y. Suzuki, K. Murakami, S. Ando, T. Higashihara, and M. Ueda. Synthesis and characterization of thianthrene-based poly(phenylene sulfide)s with high refractive index over 1.8. *Journal of Materials Chemistry*, 21(39):15727, 2011.

[52] P. K. Tapaswi, M.-C. Choi, K.-M. Jeong, S. Ando, and C.-S. Ha. Transparent aromatic polyimides derived from thiophenyl-substituted benzidines with high refractive index and small birefringence. *Macromolecules*, 48(11):3462–3474, 2015.

[53] C. A. Terraza, J.-G. Liu, Y. Nakamura, Y. Shibasaki, S. Ando, and M. Ueda. Synthesis and properties of highly refractive polyimides derived from fluorene-bridged sulfur-containing dianhydrides and diamines. *Journal of Polymer Science Part A: Polymer Chemistry*, 46(4):1510–1520, 2007.

[54] Y. Tojo, Y. Arakwa, J. Watanabe, and G. ichi Konishi. Synthesis of high refractive index and low-birefringence acrylate polymers with a tetraphenylethane skeleton in the side chain. *Polymer Chemistry*, 4(13):3807, 2013.

[55] J. Wakita, H. Sekino, K. Sakai, Y. Urano, and S. Ando. Molecular design, synthesis, and properties of highly fluorescent polyimides. *The Journal of Physical Chemistry B*, 113(46):15212–15224, 2009.

[56] G. Yang, R. Zhang, H. Huang, L. Liu, L. Wang, and Y. Chen. Synthesis of novel biobased polyimides derived from isomannide with good optical transparency, solubility and thermal stability. *RSC Adv.*, 5(83):67574–67582, 2015.

[57] H. Yeo, J. Lee, M. Goh, B.-C. Ku, H. Sohn, M. Ueda, and N.-H. You. Synthesis and characterization of high refractive index polyimides derived from 2,5- bis (4-aminophenylenesulfanyl)-3,4-ethylenedithiothiophene and aromatic dianhydrides. *Journal of Polymer Science Part A: Polymer Chemistry*, 53(7):944–950, 2015.

[58] N.-H. You, N. Fukuzaki, Y. Suzuki, Y. Nakamura, T. Higashihara, S. Ando, and M. Ueda. Synthesis of high-refractive index polyimide containing selenophene unit. *Journal of Polymer Science Part A: Polymer Chemistry*, 47(17):4428–4434, 2009.

[59] N.-H. You, T. Higashihara, S. Ando, and M. Ueda. Highly refractive polymer resin derived from sulfur-containing aromatic acrylate. *Journal of Polymer Science Part A: Polymer Chemistry*, 48(12):2604–2609, 2010.

[60] N.-H. You, T. Higashihara, S. Yasuo, S. Ando, and M. Ueda. Synthesis of sulfur-containing poly(thioester)s with high refractive indices and high abbe numbers. *Polymer Chemistry*, 1(4):480, 2010.

[61] N.-H. You, Y. Suzuki, D. Yorifuji, S. Ando, and M. Ueda. Synthesis of high refractive index polyimides derived from 1,6-bis(p-aminophenylsulfanyl)-3,4,8,9-tetrahydro-2,5,7,10-tetrathiaanthracene and aromatic dianhydrides. *Macromolecules*, 41(17):6361–6366, 2008.

[62] X. L. Yu, W. H. Yu, and X. Y. Wang. DFT-based quantum theoretic QSPR studies of the glass transition temperatures of polyacrylates. *Journal of Structural Chemistry*, 50(5):821–826, 2009.

[63] G. Zhang, H. hao Ren, D. sheng Li, S. ru Long, and J. Yang. Synthesis of highly refractive and transparent poly(arylene sulfide sulfone) based on 4,6-dichloropyrimidine and 3,6-dichloropyridazine. *Polymer*, 54(2):601–606, 2013.

[64] G. Zhang, G. shun Huang, X. jun Wang, S. ru Long, and J. Yang. Synthesis of high refractive index polyamides containing thioether unit. *Journal of Polymer Research*, 18(6):1261–1268, 2010.

[65] G. Zhang, D. ting Bai, D. sheng Li, S. ru Long, X. jun Wang, and J. Yang. Synthesis and properties of polyamides derived from 4,6-bis(4-chloroformylphenylthio)pyrimidine and 3,6-bis(4-chloroformylphenylthio)pyridazine. *Polymer International*, 62(9):1358–1367, 2013.

[66] G. Zhang, Y. xuan Zhou, Y. Li, X.-J. Wang, S.-R. Long, and J. Yang. Investigation of the synthesis and properties of isophorone and ether units based semi-aromatic polyamides. *RSC Adv.*, 5(62):49958–49967, 2015.
